# Identification of HCC Subtypes With Different Prognosis and Metabolic Patterns Based on Mitophagy

**DOI:** 10.3389/fcell.2021.799507

**Published:** 2021-12-16

**Authors:** Yao Wang, Zhen Wang, Jingjing Sun, Yeben Qian

**Affiliations:** ^1^ Department of General Surgery, The First Affiliated Hospital of Anhui Medical University, Hefei, China; ^2^ Department of General Surgery, Feixi County People’s Hospital, Hefei, China

**Keywords:** HCC, mitophagy, metabolism, ICGC data portal, the cancer genome atlas program

## Abstract

**Background:** Mitophagy is correlated with tumor initiation and development of malignancy. However, HCC heterogeneity with reference to mitophagy has yet not been systematically explored.

**Materials and Methods:** Mitophagy-related, glycolysis-related, and cholesterol biosynthesis-related gene sets were obtained from the Reactome database. Mitophagy-related and metabolism-related subtypes were identified using the ConsensusClusterPlus algorithm. Univariate Cox regression was analysis was performed to identify prognosis-related mitophagy regulators. Principal component analysis (PCA) was used to create composite measures of the prognosis-related mitophagy regulators (mitophagyscore). Individuals with a mitophagyscore higher or lower than the median value were classified in high- or low-risk groups. Kaplan-Meier survival and ROC curve analyses were utilized to evaluate the prognostic value of the mitophagyscore. The nomogram and calibration curves were plotted using the“rms” R package. The package “limma” was used for differential gene expression analysis. Differentially expressed genes (DEGs) between high- and low-risk groups were used as queries in the CMap database. R package “pRRophetic” and Genomics of Drug Sensitivity in Cancer (GDSC) database were used to determine the sensitivity of 21 previously reported anti-HCC drugs.

**Results:** Three distinct HCC subtypes with different mitophagic accumulation (low, high, and intermediate mitophagy subtypes) were identified. High mitophagy subtype had the worst outcome and highest glycolysis level. The lowest degree of hypoxia and highest cholesterol biosynthesis was observed in the low mitophagy subtype; oncogenic dedifferentiation level in the intermediate mitophagy subtype was the lowest. Mitophagyscore could serve as a novel prognostic indicator for HCC.High-risk patients had a poorer prognosis (log-rank test, *p* < 0.001). The area under the ROC curve for mitophagyscore in 1-year survival was 0.77 in the TCGA cohort and 0.75 in the ICGC cohort. Nine candidate small molecules which were potential drugs for HCC treatment were identified from the CMap database. A decline in the sensitivity towards 21 anti-HCC drugs was observed in low-risk patients by GDSC database. We also identified a novel key gene, SPP1, which was highly associated with different mitophagic subtypes.

**Conclusion:** Based on bioinformatic analyses, we systematically examined the HCC heterogeneity with reference to mitophagy and observed three distinct HCC subtypes having different prognoses and metabolic patterns.

## Introduction

Hepatocellular carcinoma (HCC), a highly heterogeneous solid malignancy, is the third leading cause of cancer-related deaths, worldwide. The overall 5-years survival rate for HCC is 18%, and it is the second deadliest malignancy after pancreatic carcinoma (Beal et al., 2017). Tumor heterogeneities, including microenvironmental discrepancies and morphological heterogeneity, are frequently reported in HCC and play a major role in tumor progression and resistance to treatment. (Li and Wang, 2016), (Borgia et al., 2021), (Atwa et al., 2021)

A special form of autophagy, mitophagy, is a mechanism for mitochondrial quality control. Damaged, defective, or unfunctional mitochondria are identified by the mitophagic machinery and subsequently degraded in the lysosome (Vara-Perez et al., 2019).The PINK1/Parkin pathway is among the most investigated pathway in mitophagy. Ordinarily, the serine/threonine PTEN-induced putative kinase 1 (PINK1) is transported to the inner mitochondrial membranes by the translocase of the outer membrane (TOM) and the translocase of the inner membrane (TIM) complexes. The presenilin-associated rhomboid-like (PARL) proteins cleave PINK1 protein and target it for degradation to the mitochondrial proteasome. Therefore, the intracellular level of PINK1 is relatively low. Mitochondrial depolarization stops the translocation of PINK1. PARL upon phosphorylation by PDK2 loses its ability to cleave PINK1. (Jin et al., 2010), (Lopez Domenech et al., 2018), (Wang et al., 2011) PINK1 accumulation promotes the E3 ubiquitin ligase activity of Parkin by the phosphorylation of serine 65 residue of the ubiquitin-like domain of Parkin. The impaired mitochondria are isolated when critical mitochondrial proteins such as MFN1, MFN2, and Miro1, are ubiquitinated by Parkin. (Glauser et al., 2011), (Chen and Dorn, 2013) In addition, phosphorylation of ubiquitin chains by PINK1 further enhances Parkin recruitment and activation (Durcan and Fon, 2015). Subsequently, p62 and OPTN (autophagy cargo adaptors) recognize the polyubiquitylation of mitochondrial proteins and ultimately the autophagic machinery degrades the complex formed by the interaction of mitochondrial proteins and LC3. (Wong and Holzbaur, 2014), (Geisler et al., 2010)

Hepatocytes each contain approximately 1,000 mitochondria, which constitute about 18% of cell by volume (Koh et al., 2018).Previous studies have reported that Mitochondria are a major source of reactive oxygen species (ROS) production. Meanwhile, immoderate ROS lead to DNA, protein, and lipids lesion that tightly associated to the pathogenesis of cancers. By the way, Over 90% of HCCs are intimately linked to hepatic injury and inflammation. Dysfunctional mitochondria allows the release of ROS and mitochondrial DNA (mtDNA) into the cytosol.The condition can activate the major innate immune response and result in HCC initiation and progression. (Schieber and Chandel, 2014), (Severi et al., 2010) Therefore, mitophagy plays an essential role in preventsing HCC tumorigenesis which is extremely important for relieving intracellular oxidative stress by removing damaged mitochondria.

However, uncontrolled proliferation of tumor cells need mitophagy to ensure the normal mitochondrial homeostasis, dysfunction of which will disrupt metabolism and increase oxidative stress, inducing tumor cell apoptosis (Ferro et al., 2020). Furthermore, mitophagy mediated through PINK1 lead to tumor suppressor p53 inactivation in mitochondria, which is considered to be a significant role to maintain the HCC stem cell (CSCs) quantity (Liu et al., 2017). In summary, mitophagy prevents HCC tumorigenesis by suppressing dysfunctional mitochondria accumulation, cellular oxidative stress, genome instability and inflammation. When a tumor mass forms, conversely, mitophagy is hyperactivated to meet cancer cells metabolism demand and facilitate HCC progression.

Therefore, mitophagy might become a promising but challenging therapeutic direction for HCC in the future and exploring tumor heterogeneity of mitophagy accumulation is of great significance for HCC prevention and treatment.

## Materials and Methods

### Data Acquisition and Processing

Data sets of transcriptomic sequencing and clinical details were downloaded from the TCGA and ICGC databases.

After excluding the cases having a follow-up time of less than 30 days, a total of 571 cases (342 cases from TCGA-LIHC and 229 cases from ICGC-LIRI-JP databases) were included in this study. TCGA-LIHC cohort was designated as the training set, and the ICGC-LIRI-JP cohort was designated as the test set.

Reactome database was used to obtain data for three mitophagy-related signaling pathways, including mitophagy (R-HSA-5205647), pink1 prkn mediated mitophagy (R-HSA-5205685) and receptor mediated mitophagy (R-HSA-8934903). Based on the union of these gene sets, 28 regulators of mitophagy were identified (Supplementary Table S1). Two additional gene sets, for cholesterol biosynthesis (R-HSA-191273) and glycolysis (R-HSA-70171), were also downloaded from the Reactome database (Supplementary Table S1).

Malta et al. developed a new machine learning algorithm named one-class logistic regression (OCLR) to evaluate the transcriptome and epigenetic signatures. OCLR-based transcriptomic or epigenetic feature set was generalized to TCGA database to estimate the stemness index, with the mRNA expression-based stemness index (mRNAsi) reflecting gene expression, and the epigenetically regulated (EREG)-mRNAsi reflecting epigenetically regulated mRNAsi. The stemness index contributes to elucidation of the dedifferentiation of tumor cells, and higher index values were closely associated with the progression of multiple types of cancers (Malta et al., 2018). The mRNAsi scores for the TCGA-LIHC samples were obtained from previous studies (Malta et al., 2018). mRNAsi score is a novel gene expression-based stemness index for assessing the degree of oncogenic de-differentiation; the value ranges from (0,1).

### Identification of Mitophagy Subtypes

Unsupervised hierarchical clustering was performed using the “ConsensusClusterPlus” package in R to cluster the mitophagy regulators. The parameter settings were as follows: reps = 50, pItem = 0.8, pFeature = 1, and distance = Euclidean (Wilkerson and Hayes, 2010). Mitophagy subtypes were obtained. For a total of 97 genes from the cholesterol biosynthesis and glycolysis gene sets, unsupervised hierarchical clustering was performed with the above settings and different metabolic classifications were obtained.

We also calculated the enrichment scores for each sample in the cholesterol biosynthesis and glycolysis gene sets by the single sample Gene Set Enrichment Analysis (ssGSEA) algorithm (Supplementary Table S2) (Hänzelmann et al., 2013).

### Construction and Validation of the Prognostic Mitophagy Regulator-Based Signature

Univariate Cox analysis was used to distinguish between prognosis-related mitophagy regulators. The overlapping prognostic mitophagy regulators (*p* < 0.05) in TCGA-LIHC and ICGC-LIRI-JP cohorts were selected for further analyses. Principal component analysis (PCA) was performed to establish the mitophagy-related gene signature. The sum of the principal components 1 and 2 gave the mitophagy regulator signature scores (mitophagyscore). The approach focused on the signature scores in the set with the largest block of well correlated (or anticorrelated) genes, while down-weighing the contributions from genes that were not associated with other set members. The mitophagyscore was identified by performing a method similar to GGI. (Sotiriou et al., 2006), (Zeng et al., 2019)

We calculated the mitophagyscore for each patient and divided them into the high-risk and low-risk groups based on the median in their respective cohorts. K-M analysis and log-rank test were used to evaluate the prognostic value of the mitophagyscore. Based on tumor stage and mitophagyscore we plotted the nomogram for predicting survival outcomes using the “rms” package in R (Huang et al., 2020a). Calibration plots were plotted to compare the predictive efficacy of nomograms for the 1‐, 3‐, and 5‐years OS probability.

### Screen for Candidate Small Molecule Drugs

Differentially expressed genes (DEGs) between the high-risk and low-risk groups were identified using the“Limma” package in R (parameters: |fold change| >2 and FDR-adjusted *p*-value < 0.05) (Xu et al., 2020). A Venn diagram was drawn to depict the intersection of DEGs in the TCGA-LIHC and ICGC-LIRI-JP cohorts; these overlapping DEGs were selected for further downstream analyses. Functional enrichment analysis for the intersecting DEGs was performed using the“clusterProfiler” package in R (Liu et al., 2020). Enrichment analysis results of the top 20 enriched KEGG and GO terms with smallest adjusted *p*-value are displayed in dotplot. Then we rerun the enrichment analysis by Metascape database (http://metascape.org) to verify each top 20 enriched KEGG and GO terms. The intersection of DEGs was used as the input for the Connectivity map (CMap) database (https://portals.broadinstitute.org/cmap/). The selection criteria were as follows: *p*-value<0.05, enrichment < −0.8 (Shen et al., 2019).

### Drug Sensitivity Prediction

Drug-response prediction was assessed using the“pRRophetic” package in R, where the half-maximum inhibitory concentration (IC50) of each patient was estimated using Ridge’s regression, and the accuracy of the prediction was estimated by 10-fold cross-validation, based on the Genomics of Drug Sensitivity in Cancer (GDSC) database (Ding et al., 2021).

### Identification of DEGs Among Different Mitophagy Subtypes

We obtained multiple distinct mitophagy subtypes and gene differential expression analysis was performed using the“limma” package in R for pairwise analyses to evaluate specific DEGs between the subtypes. The screening criteria were set as follows: | log2FC |> 1 and adjusted *p* < 0.05. This process was performed for both TCGA-LIHC and ICGC-LIRI-JP cohorts simultaneously. The intersecting DEGs from the two cohorts were considered as candidate DEGs which were highly correlated with the mitophagy subtypes. The difference in expression of candidate DEGs between tumor and normal tissues was estimated using the GEPIA database. The expression of candidate DEGs in different tumor stages and their effect on HCC prognosis were assessed using the GEPIA database (Tang et al., 2017).

The HPA database was used to obtain protein expression levels of candidate DEGs based on immunohistochemistry (IHC) staining and IHC image data downloaded from the HPA database (Song et al., 2020).

### Statistical Analysis

Statistical tests were carried out by R software for statistical computing (R version 4.0.4). Significance was estimated via the nonparametric Wilcoxon test in comparisons between two groups, while via Kruskal-Wallis test in multiple comparisons. Categorical data were tested by the chi-square test and the chi-square test for trends. K-M analysis for OS and Progression-free survival (PFS) was performed between different subgroups, followed by log-rank test. Operating characteristic curve (ROC) for 1-year survival was established for evaluation of the predictive efficacy of the mitophagyscore. Correlation analyses were performed using Spearman’s correlation. A statistical *p* value of 0.05 was considered indicative of significance. Enrichment analysis of all Reactome pathways performed by GSEA (https://software.broadinstitute.org/gsea/index.jsp).Under the premise of NOM p-val less than 0.05, only the top ten pathways with highest absolute values of the normalized Enrichment Score (NES) are presented as the most enriched items.

## Results

### Landscape of Mitophagy Regulators in Liver HCC

**FIGURE 1:**
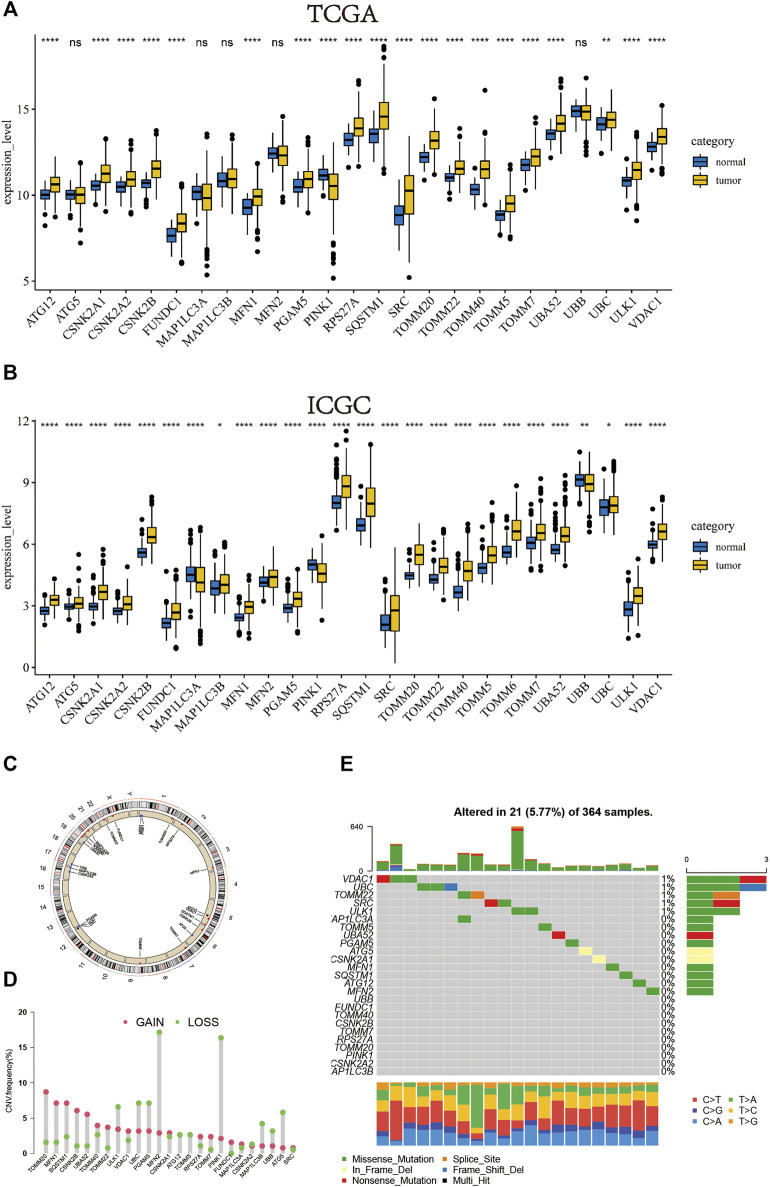
In TCGA cohort, the boxplot of the expression level of the mitophagy regulators in normal and HCC samples **(B)** In ICGC cohort, the boxplot of the expression level of the mitophagy regulators in normal and HCC samples **(C)** The location of CNV alteration of mitophagy regulators on chromosomes using TCGA cohort **(D)** The CNV variation frequency of mitophagy regulators in TCGA cohort. The height of the column represented the alteration frequency. The deletion frequency, green dot; The amplification frequency, red dot **(E)** The mutation frequency of mitophagy regulators in 364 patients with HCC from TCGA-LIHC cohort. Each column represented individual patients. The upper barplot showed TMB, The number on the right indicated the mutation frequency in each regulator. The right barplot showed the proportion of each variant type. The stacked barplot below showed fraction of conversions in each sample. The asterisks represented the statistical *p* value (*p < 0.05; **p < 0.01; ***p < 0.001; ****p < 0.0001).

We separately investigated the levels of expression of mitophagy regulators in TCGA-LIHC and ICGC-LIRI-JP cohorts (Figure 1A, B). Genes detected in less than two cohorts were excluded and we obtained a total of 26 mitophagy regulators in this study. However, the expression of TOMM6 was zero in more than 90% of samples in TCGA cohort.Therefore, TOMM6 were not included for futher analysis of TCGA cohort (Zoni et al., 2019). Among the 26 genes, only PINK1, MAPILC3A and UBB were significantly downregulated in tumor samples as compared to normal samples and other genes were significantly upregulated in tumor samples. The locations of CNV alterations in mitophagy regulators on chromosomes are shown in Figure 1C. Compared with normal liver tissues, PINK1 and MFN2 showed a distinctly higher proportion of copy number alterations and 13 mitophagy regulators showed a higher proportion of gain in CNVs (e.g. TOMM20 and MLN1) (Figure 1D). Mitophagy regulators were rarely mutated; the frequency was 5.77%. The results indicated that VDAC1 and UBC exhibited the highest mutation frequency.

### Description of Mitophagy Subtypes in Liver HCC

**FIGURE 2:**
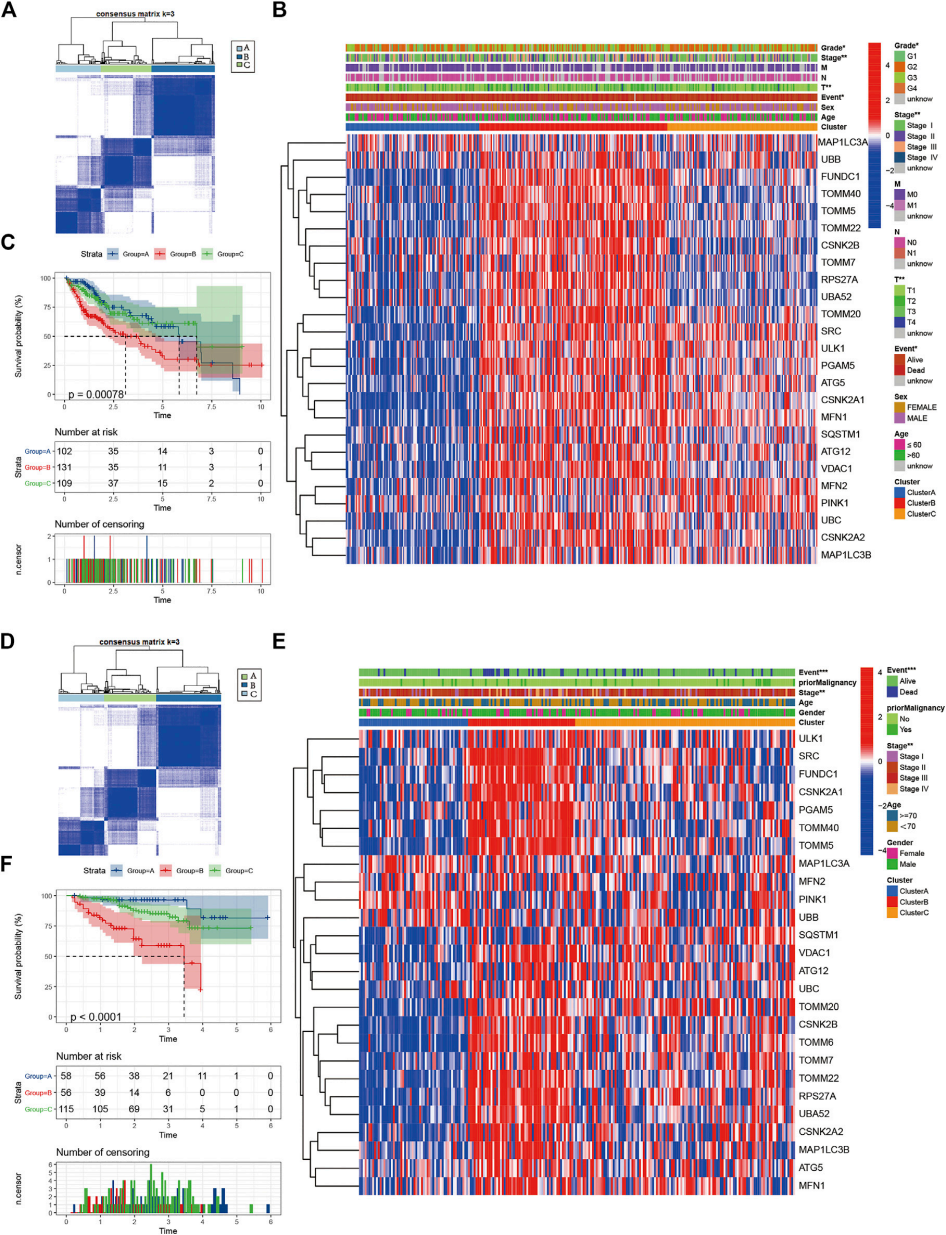
Identify different mitophagy subtypes **(A)** Consensus matrices of the TCGA cohort for k = 3 **(B)** Unsupervised clustering of overlapping mitophagy regulators in TCGA cohorts to classify patients into different subtypes (cluster A-C). Grade, stage, gender, survival status and age were utilized as patient annotations **(C)** Survival analyses for the three clusters in TCGA cohort including 102 cases in cluster A, 131 cases in cluster B, and 109 cases in cluster C. Kaplan-Meier curves with Log-rank *p* value < 0.001 showed a significant survival difference among three clusters. The cluster B showed significantly worse OS than the other two clusters **(D)** Consensus matrices of the ICGC cohort for k = 3 **(E)** Unsupervised clustering of overlapping mitophagy regulators in ICGC cohorts to classify patients into different subtypes (cluster A-C). Stage, gender, survival status and age were utilized as patient annotations **(F)** Survival analyses for the three clusters in ICGC cohort including 58 cases in cluster A, 56 cases in cluster B, and 115 cases in cluster C. Kaplan-Meier curves with Log-rank *p* value < 0.001 showed a significant survival difference among three clusters. The cluster B showed significantly worse OS than the other two clusters.

**FIGURE 3:**
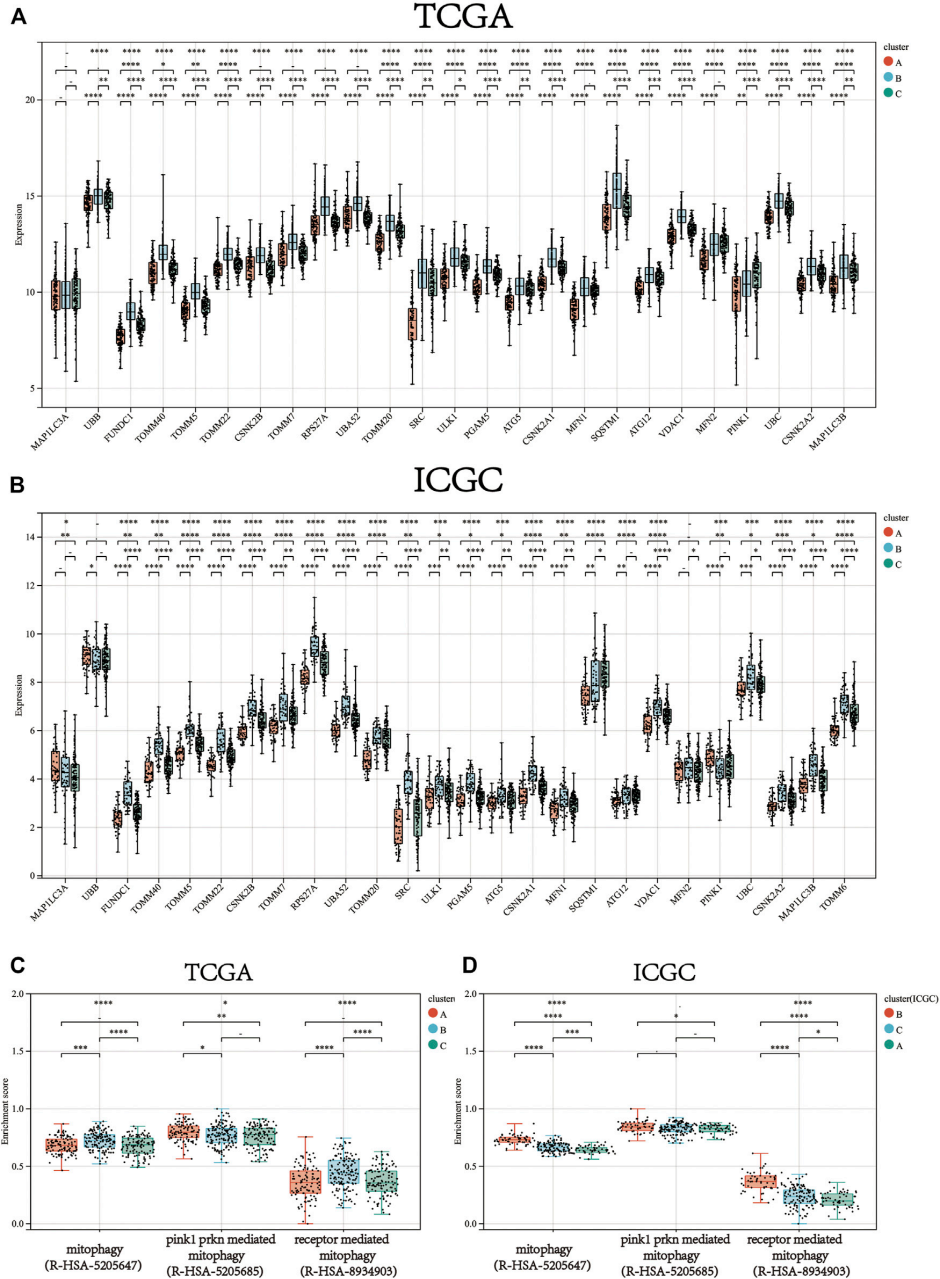
The gene expression of each mitophagy regulators between the different mitophagy subtypes in TCGA LIHC **(A)** and ICGC LIRI-JP **(B)**.The enrichment score of three mitophagy-relayed gene sets between the different mitophagy subtypes in TCGA LIHC **(C)** and ICGC LIRI-JP **(D)**. The upper and lower ends of the boxes represented interquartile range of values. The lines in the boxes represented median value, and black dots showed outliers. The asterisks represented the statistical *p* value (*p < 0.05; **p < 0.01; ***p < 0.001; ****p < 0.0001).

Consistent clustering of 26 mitophagy regulators was established using the “ConsensusClusterPlus” package in R and all tumor samples were classified into three subtypes (cluster A, cluster B, and cluster C) (Figure 2A). The gene expression pattern of mitophagy regulators is shown using a heatmap of hierarchical clustering (Figure 2B). Kaplan-Meier survival analyses were performed for the three clusters (Figure 2C). The analysis procedure was identical for both TCGA-LIHC and ICGC-LIRI-JP cohorts (Figure 2D–F). A vast majority of the mitophagy regulators had their highest expression levels in cluster B and the lowest in cluster A (Figure 3A, B). ssGSEA was employed to impute the enrichment scores of mitophagy-related pathways.Neither in the TCGA-LIHC nor the ICGC-LIRI-JP cohorts, the enrichment scores of mitophagy pathway and receptor mediated mitophagy pathway were significantly highest in cluster B (Figure 3C, D).

We then defined the samples as low mitophagy (cluster A), high mitophagy (cluster B), and intermediate mitophagy (cluster C) subtypes. Patients with high mitophagy subtype had the worst prognoses.

**FIGURE 4:**
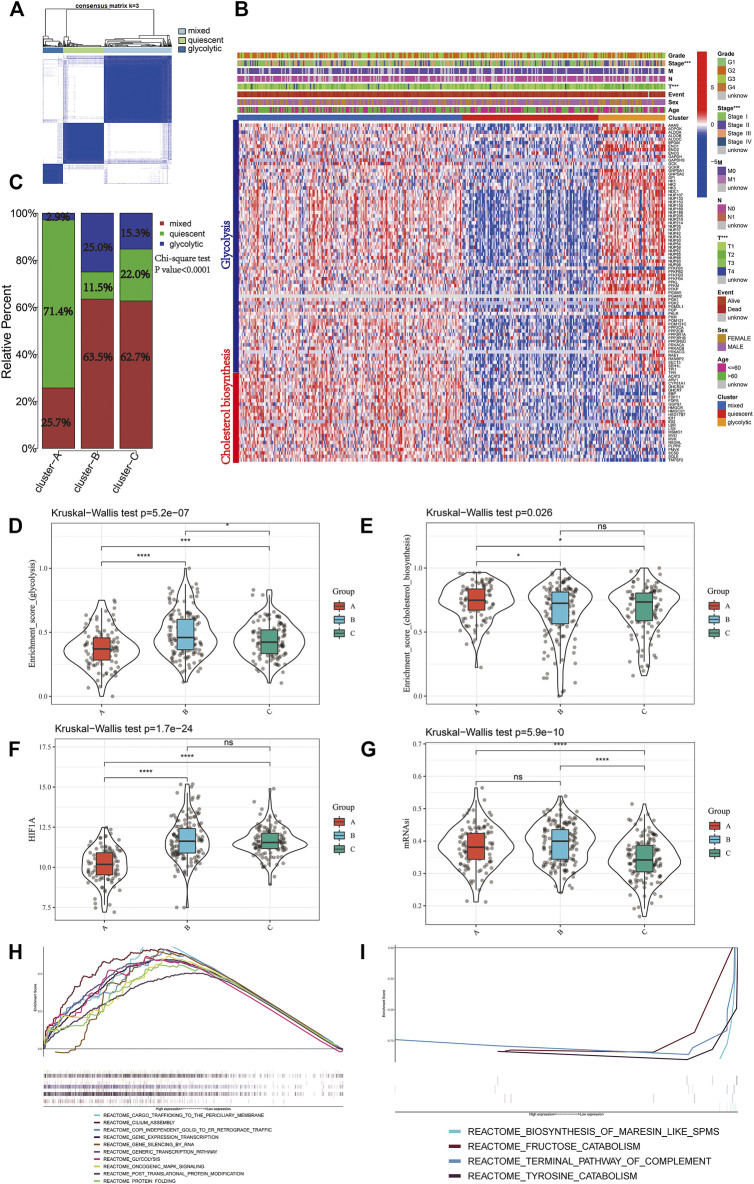
Identify different metabolic subtypes **(A)** Consensus matrices of the TCGA cohort for k = 3 **(B)** Unsupervised clustering of total 97 genes from the cholesterol biosynthesis and glycolysis gene sets in TCGA cohorts to classify patients into different subtypes (mixed, quiescent, and glycolytic). Grade, stage, gender, survival status and age were utilized as patient annotations **(C)** The proportion of metabolic subtypes in the three mitophagy subtypes. Mixed subtype, red; quiescent subtype, green; glycolytic subtype, blue. The enrichment score of glycolysis **(D)** and cholesterol biosynthesis **(E)** gene sets between the different mitophagy subtypes in TCGA LIHC **(F)** Differences in HIF1A expression level among three mitophagy subtypes in TCGA LIHC cohort **(G)** Differences in mRNAsi score among three mitophagy subtypes in TCGA LIHC cohort. The KruskalWallis test was used to compare the statistical difference between three subtypes. GSEA results enriched in cluster A **(H)** and cluster B **(I)**. The asterisks represented the statistical *p* value (*p < 0.05; **p < 0.01; ***p < 0.001; ****p < 0.0001).

A total of 97 genes from cholesterol biosynthesis and glycolysis gene sets were used for unsupervised hierarchical clustering and three metabolic classifications were obtained in the TCGA-LIHC cohort (mixed, quiescent, and glycolytic; Figure 4A). The gene expression patterns for cholesterol biosynthesis and glycolysis genes in the TCGA-LIHC cohort are shown in Figure 4B. Cluster A, the low mitophagy subtype, showed a particularly prominent advantage in quiescent metabolic classification (71.4%) and was inferior in glycolytic classification (2.9%). In contrast, there was a significantly greater proportion of mixed metabolic classification and a smaller proportion of quiescent classification in clusters B and C as compared to cluster A (Chi-square test *p*-value<0.0001; Figure 4C); a smaller proportion of mixed metabolic classification and a larger proportion of quiescent classification was found in cluster B as compared to cluster C (Chi-square test *p* value = 0.023; Figure 4C).

Kruskal-Wallis test showed significant differences in the enrichment scores for cholesterol biosynthesis and glycolysis genes between the mitophagy subtypes. Cluster A had the lowest median enrichment score for glycolysis genes and highest enrichment score for cholesterol biosynthesis genes, while cluster B had the highest median enrichment score for glycolysis genes (Figure 4D, E). This indicated that the mitophagy subtypes were strongly associated with the metabolic differences in liver HCC.

We compared the gene expression of HIF1A, a hypoxia marker, between different mitophagy subtypes, and observed a significant downregulation in cluster A (Figure 4F).

The median mRNAsi score was the highest in cluster C and there were no significant mRNAsi score differences between clusters A and B. (Figure 4G).

To examine the change in biological functions upon the increase in mitophagic accumulation, we performed GSEA for clusters A and B. Ten most enriched terms in cluster B are shown in Figure 4H; four other terms were enriched in cluster A (Figure 4I) including the terminal pathway of the complement system, biosynthesis of maresin-like SPMs, fructose catabolism, and tyrosine catabolism pathways.

### Prognostic Role of Mitophagy Regulators in HCC

Univariate analysis of mitophagy regulators for OS indicated that there were 18 potential prognostic mitophagy regulators in the TCGA-LIHC cohort and 15 in the ICGC-LIRI-JP cohort (Supplementary Figure S1A, B). Ultimately, 12 mitophagy regulators were identified as predictors for poor prognosis in both cohorts, including PGAM5, TOMM22, TOMM5, MFN1, CSNK2A2, VDAC1, TOMM40, FUNDC1, CSNK2A1, CSNK2B, RPS27A, and SRC were included in subsequent analyses.

PCA analysis reduced the above 12 mitophagy regulators into two principal components, PC1 and PC2. We summated the values of PC1 and PC2 for each patient and calculated the mitophagyscore.

**FIGURE 5:**
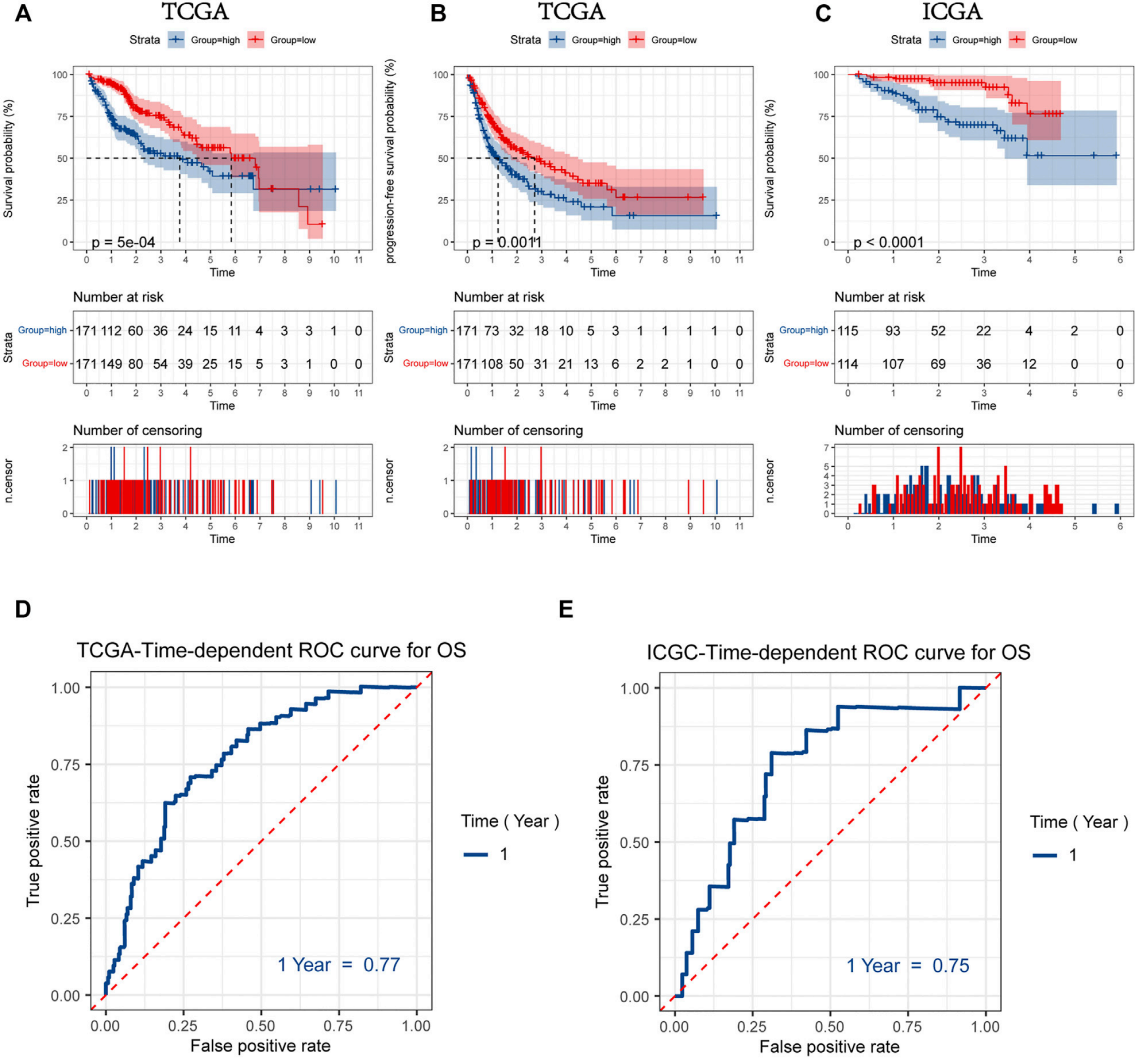
Kaplan-Meier plots for overall survival (OS) **(A)** and progression-free survival (PFS) **(B)** of HCC patients with a high or low mitophagyscore in TCGA LIHC cohort **(C)** Kaplan-Meier plots for overall survival (OS) of HCC patients with a high or low mitophagyscore in ICGC cohort. ROC curves for predicting 1-year OS by mitophagyscore TCGA LIHC cohort **(D)** and ICGC cohort **(E)**.

Patients were divided into two groups according to the median values for mitophagyscore (median value in TCGA = 0.060; median value in ICGC = -10.353). Patients with mitophagyscores below the median were classified in the low-risk group (TCGA:n = 171; ICGC:n = 114) and those with higher mitophagyscores values were classified into the high-risk group (TCGA:n = 171; ICGC:n = 115). In the TCGA cohort, the low-risk group showed a significant advantage in OS (Figure 5A; HR = 1.88, 95%CI = 1.31–2.70, *p* < 0.001) and PFS (Figure 5B; HR = 1.61, 95%CI = 1.21–2.19, *p* = 0.0011). The median OS (3.7 years) and the median PFS (1.2 years) in the high-risk group were shorter than those in the low-risk group (median OS: 5.8 years; median PFS: 2.5 years). A similar result was observed in the ICGC-LIRI-JP cohort, that the high-risk group had a worse prognosis as compared to the low-risk group (Figure 5C; HR = 4.35, 95%CI = 2.07–9.17, *p* < 0.001).

The ROC curve was plotted to estimate the predictive ability of mitophagyscore. The area under the ROC curve for OS at 1-year was 0.77 in the TCGA-LIHC cohort and 0.75 in the ICGC-LIRI-JP cohort (Figure 5D, E).

**FIGURE 6:**
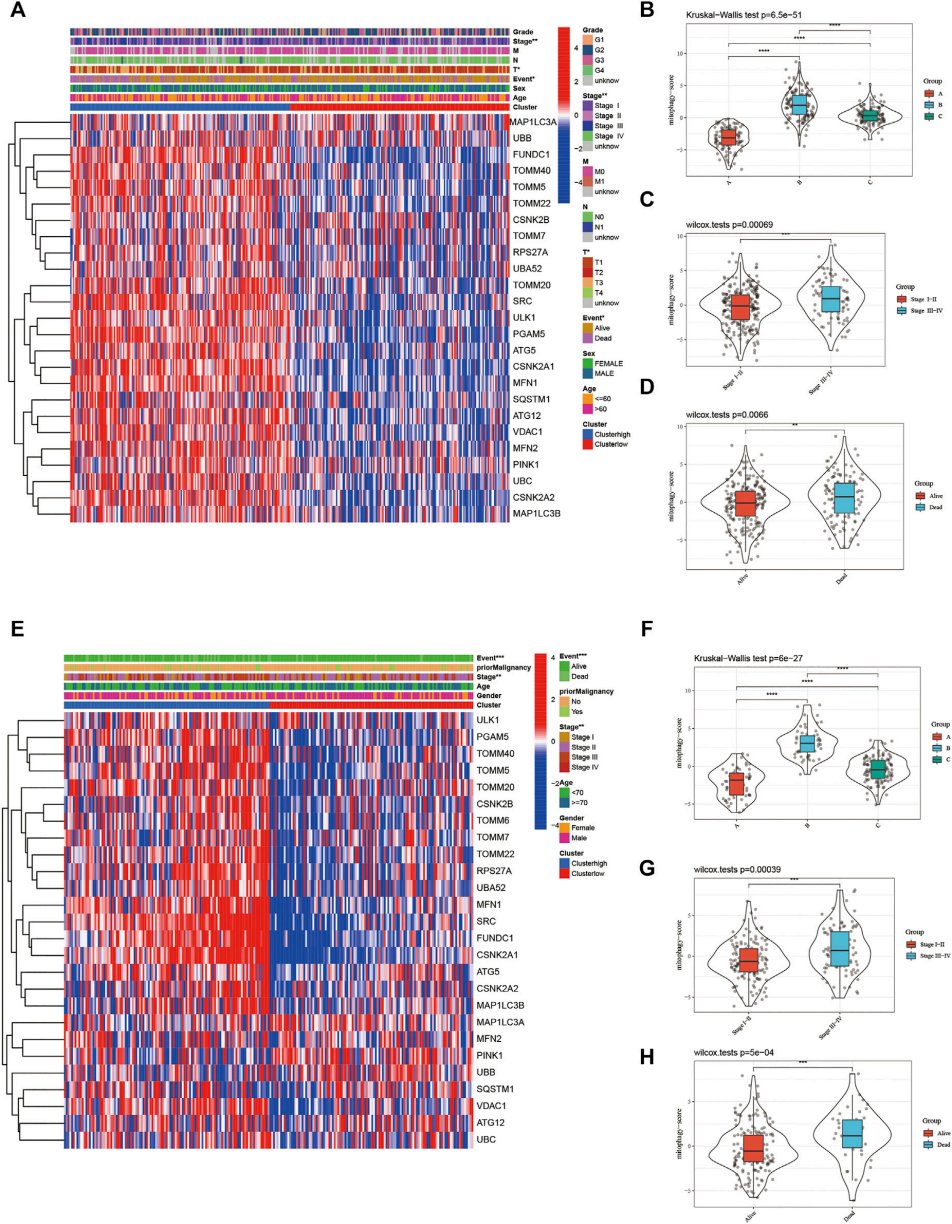
**(A)** The heatmap of gene expression in the high-risk group versus low-risk group for the mitophagy regulators in TCGA LIHC cohort. Violin plot of different mitophagyscores between different mitophagy subtypes **(B)**, stages (**C)** and survival status **(D)** of TCGA LIHC cohort **(E)** The heatmap of gene expression in the high-risk group versus low-risk group for the mitophagy regulators in ICGC cohort. Violin plot of different mitophagyscores between different mitophagy subtypes **(F)**, stages **(G)** and survival status **(H)** of ICGC cohort. The asterisks represented the statistical *p* value (*p < 0.05; **p < 0.01; ***p < 0.001; ****p < 0.0001).

The heat map in Figure 6A represents the relative expression of mitophagy regulators; the vast majority of them showed increased expression in the high-risk group. The mitophagyscore in the high mitophagy group (cluster B) was the highest, followed by that in the intermediate subtype (cluster C), and the low mitophagy (cluster A) group (Figure 6B). The mitophagyscore in the early stages of HCC was significantly higher as compared to the advanced tumor stage (Figure 6C). Mitophagyscores of patients who died during the follow-up were significantly higher as compared to those who were alive (Figure 6D). In addition, identical findings were obtained in the ICGC-LIRI-JP cohort (Figure 6E–H).

Furthermore, we stratified both the TCGA-LIHC and ICGC-LIRI-JP cohorts based on clinical features and performed K-M survival analysis to determine whether the prognostic value of the mitophagyscores was independent of other clinical characteristics. The result indicated that mitophagyscores had satisfactory prognostic prediction efficacy in different groups stratified by clinical features (Supplementary Figure S2).

**FIGURE 7:**
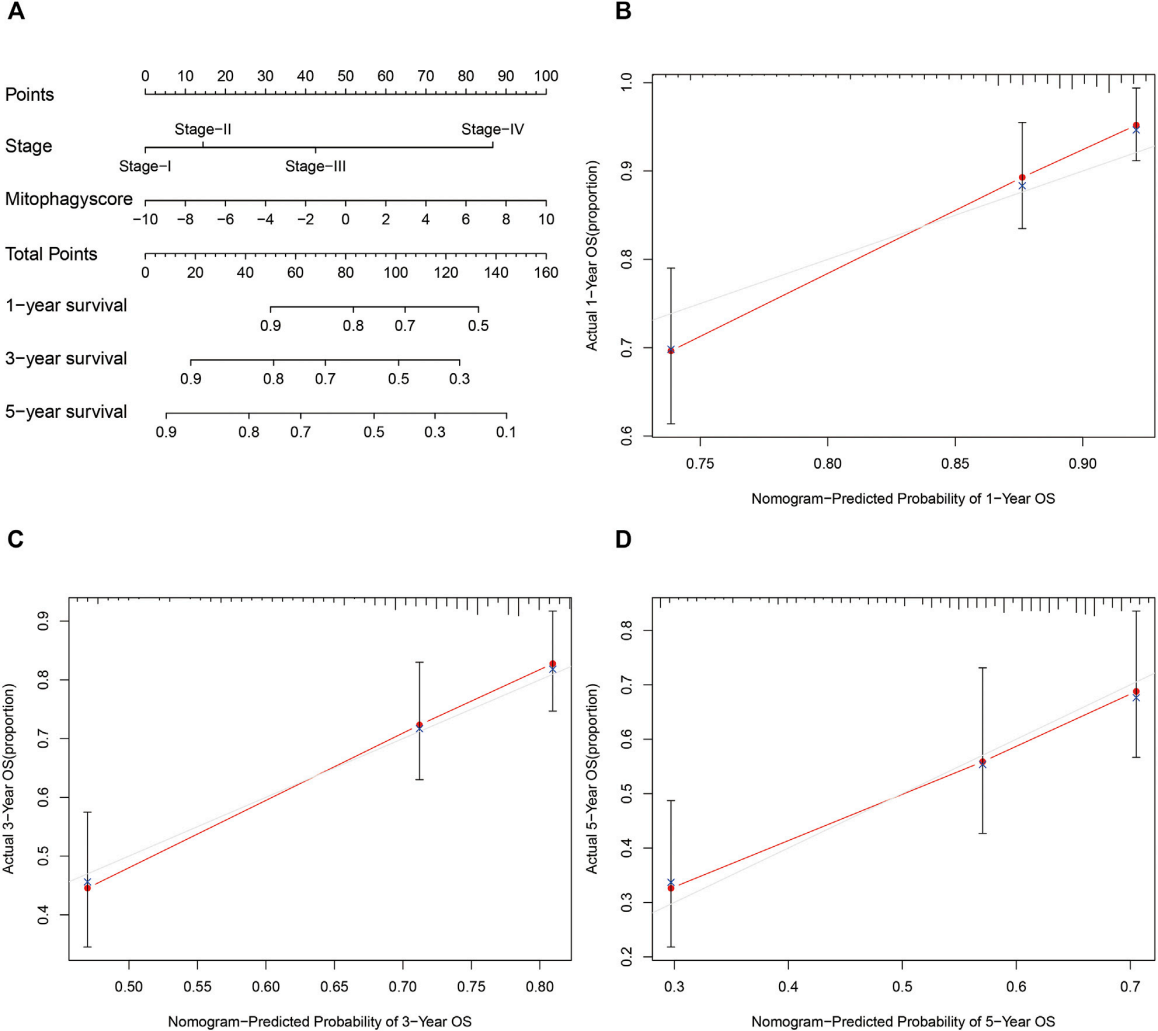
Developed nomogram to predict the probability of survival in HCC patients **(A)** Nomogram built with tumor stage and mitophagyscore incorporated estimating 1-, 3-,5-years OS for HCC patients in the TCGA LIHC cohort. The calibration curves describing the consistency between predicted and observed 1-**(B)**, 3- **(C)**,5-**(D)** year OS at different time points in the TCGA LIHC cohort. The estimated survival was plotted on the *x*-axes, and the actual outcomes were plotted on the *y*-axes. The gray 45-degree dotted line represents an ideal calibration mode.

The prognostic nomogram was then constructed based on the stage and mitophagyscores to facilitate further clinical prognostic prediction (Figure 7A). The calibration curve for prediction of survival at 1-year (Figure 7B), 3-years (Figure 7C), and 5-years (Figure 7D) in the TCGA-LIHC cohort validated the predictive accuracy of the nomogram.

### Therapeutic Potential of Mitophagyscore in HCC

Using the “Limma” package in R, a total of 197 upregulated genes and 64 downregulated genes were identified in the high-risk group (Supplementary Figure S3A, B). The significant enrichment of biology processes was assessed using the “clusterProfiler” package in R; 20 GO terms and KEGG pathways with the smallest *p*-values are shown (Supplementary Figure S3E–F). In addition, each top 20 enriched KEGG and GO terms werer validated on Metascape database and the results of the two methods were consistent. The details of functional enrichment analysis are provided in Supplementary Table S3.

CMap analysis was utilized to explore small molecular compounds having the potential to reverse the clinical manifestations in the high-risk group, such as the poor prognosis. A total of nine drugs met our selection criteria (Table 1). Notably, DL-thiorphan with an enrichment score of -0.94 showed extraordinary therapeutic potential.

**TABLE 1:** small molecular compounds having the potential to reverse the clinical manifestations in the high-risk group.

Cmap name	Mean	Enrichment	*p*	Specificity
DL−thiorphan	−0.788	−0.941	0.00742	0.0171
sanguinarine	−0.724	−0.884	0.02696	0.05
chrysin	−0.749	−0.858	0.00571	0.0152
blebbistatin	−0.7	−0.853	0.04298	0.0811
metyrapone	−0.727	−0.834	0.00137	0.0053
medrysone	−0.757	−0.826	0.00006	0.0054
verteporfin	−0.731	−0.822	0.0113	0.0465
apigenin	−0.8	−0.82	0.00199	0.0272
meticrane	−0.756	−0.804	0.00064	0

**FIGURE 8:**
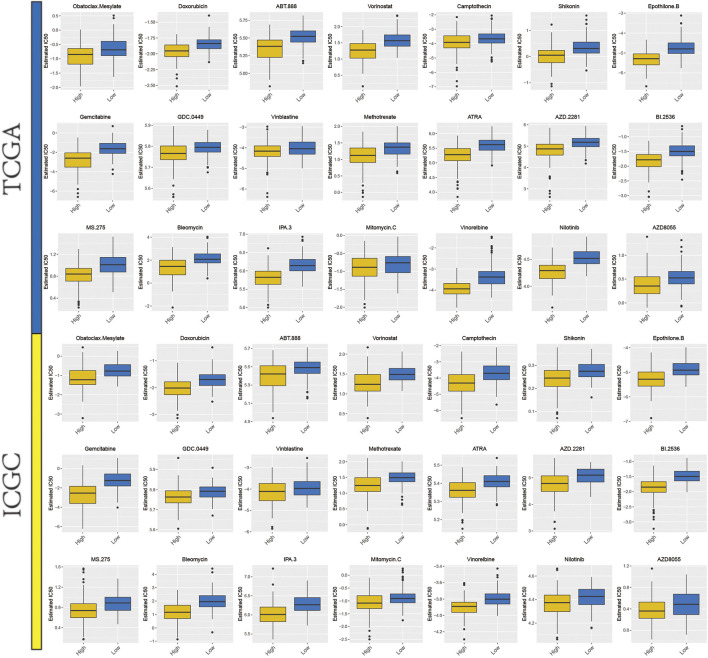
A total of 21 potential therapeutic drugs in HCC with differential IC50 between high- and low-mitophagyscore groups.

From previous studies, we collated 21 drugs with reported and experimentally verified the therapeutic potential for HCC (Supplementary Table S4). The “pRRophetic” algorithm was used to predict the sensitivity in high- or low-risk groups to the above-mentioned 21 drugs. Higher estimated IC50 values were obtained in the low-risk group as compared to the high-risk group and this result indicated that higher mitophagyscore could predict increased sensitivity towards these therapeutic drugs in HCC patients (Figure 8).

### Candidate DEGs Highly Correlate With the Mitophagy Subtypes

Differential expression of SPP1 was identified as an important factor for diversity in mitophagy subtypes (Supplementary Figure S4). GEPIA-generated box plot was used to compare the tissue-based expression patterns of SPP1 between HCC (TCGA tumor) and paired normal adjacent tissue samples (TCGA normal + GTEx normal). The expression level of SPP1 was significantly high in tumors (*p* < 0.05) (Supplementary Figure S5A). The relative expression level of SPP1 was significantly lower in stage I tumor tissues (Supplementary Figure S5B). A high expression of SPP1 (>median value) indicated a worse prognosis of HCC patients (Supplementary Figure S5C). Additionally, IHC staining data from the HPA database indicated that SPP1 had a moderate signal in HCC tissues (Supplementary Figure S5D) but was not detected in normal liver tissues (Supplementary Figure S5E); it was mainly localized in the Golgi apparatus. Spearman’s correlation analysis suggested that SPP1 was significantly positively correlated with.

Several mitophagy regulators (*ρ* = 0.43; *p* < 0.0001; Supplementary Figure S5F) and mitophagyscore (*ρ* = 0.43; *p* < 0.0001; Supplementary Figure S5G). The lowest expression of SPP1 was found in cluster A, while the highest was in cluster B (Supplementary Figure S5H). Three mitophagy-related signaling pathways (R-HSA-5205647.4, R-HSA-5205685.3, and R-HSA-8934903.3) were upregulated in A high SPP1 (>median value) expression (Supplementary Figure S5I).

## Discussion

Mitophagy in inflammation, innate immunity, and tumor progression owing to the interplay between mitophagy regulators, has gained widespread attention from researchers. (Zhou et al., 2011), (Sliter et al., 2018) The majority of previous studies focus on individual regulators; the overall characterization of integrated roles of multiple mitophagy regulators has not yet been comprehensively elucidated. Recognizing the distinct mitophagy alteration patterns in HCC will deepen the insight for tumorigenesis and cancer progression and shed light for innovation on strategies for treatment and prognosis of HCC.

In our study, we investigated the mRNA expression levels of mitophagy regulators between normal and HCC samples, and found that the alterations of CNV could be the prominent factors resulting in perturbations on some mitophagy regulators expression,particularly on PINK1. Compared to normal tissues, PINK1 with the high-frequency of copy number losses showed significantly lower expression in HCC tissues. PINK1 was reported as the critical initiator of mitophagy (Lazarou et al., 2015). Therefore, such parallel relationships between CNV and gene expression in PINK1 may have contributed to the difference in the extent of mitophagy between HCC and normal tissues. In addition, three distinct mitophagy alteration patterns were identified based on mitophagy regulators that may indicate the difference in mitophagy accumulation. Furthermore, the results demonstrated that high mitophagy accumulation was an indicator of poor prognosis. Comparatively, low mitophagy accumulation showed better prognoses in patients, particularly in the ICGC-LIRI-JP cohort.

Mitophagy, a specific autophagy type, guarantees the selective destruction of damaged or dysfunctional mitochondria. Because of the indispensable role of mitochondria in vital metabolic processes and bioenergetic functions, it is not unexpected that mitophagy is inextricably related to the metabolism of cancer cells to meet their bioenergetic needs. In pursuit of the mechanisms by which the high mitophagy subtype showed the worst prognoses, we turned our attention to the distinct metabolic patterns. Higher HIF1A expression level as compared to low mitophagy subtype indicated that in high mitophagy and intermediate subtype, tumors experienced greater hypoxic environments. Previous studies report that FUNDC1, a mitophagy regulator, interacts with LC3 through its typical LC3-binding motif Y (18)xxL (21), and mutation in this region impairs its interaction with LC3 and the subsequent induction of mitophagy under hypoxia; this may be responsible for high mitophagy accumulation. Hypoxia leads to glycolysis and lactate production. Lactate, generated during glycolysis, decreases the tumor environmental pH and an acidic pH distinctly impedes the function of normal immune cells, such as T-cell and tumor-infiltrating lymphocytes. (Lardner, 2001), (Calcinotto et al., 2012) Furthermore, the lactate-mediated enhancement of tumor cell motility is reported not only in single-cell motion but also in enforced bulk migration (Goetze et al., 2011). Hyaluronan, synthesized by tumor-associated fibroblasts (TAF), increases around the carcinoma regions at high lactate concentrations and encourages the growth and motility of cancer cells. (Stern, 2008), (Walenta and Mueller-Klieser, 2004) Taken together, these findings may at least partially explain the facilitation of tumor progression and the worse prognosis in the high mitophagy subtype as compared to the low mitophagy subtype. Furthermore, by comparing the intermediate and high mitophagy subtypes, we found a particularly large difference in the mRNAsi score, a quantification for cancer stem cells (CSCs) characteristics. As tumor-initiating cells, the CSCs have the remarkable ability of self-renewal and give rise to cells of the same phenotype or tumor cell progenitors (Lapidot et al., 1994). CSCs are tightly linked with cancer drug resistance and poor prognosis. The lower mRNAsi score in the intermediate subtype may be one of the contributors to better prognoses of patients in this group. (Zhu et al., 2018), (Pei et al., 2020)

Cancer cells require high levels of cholesterol for membrane biogenesis and in general, cholesterol metabolism promotes cancer cell proliferation, migration and invasion (Huang et al., 2020b).In our study, cholesterol biosynthesis pathway is the most enriched in low mitophagy subtype. This is hinting at the possibility of an interaction between cholesterol metabolismr and mitophagy. Research by Vicente et al. indicates that intracellular cholesterol enrichment downregulate mitophagy induced by amyloid beta (Aβ). Moreover, cholesterol accumulation in mouse models of SREBF2 (a cholesterol-related transcription factor) over-expression reduces mitophagosome formation by cousing cytosolic aggregation of the mitophagy adaptor OPTN (optineurin) (Roca-Agujetas et al., 2021). This is partly in agreement with our results.

Based on 12 mitophagy regulators, a risk score model (mitophagyscore) was constructed to estimate the accumulation of mitophagy; this was found to be a reliable prognostic indicator of HCC. The prognostic predictive ability of the mitophagyscore was stable across subgroups stratified by age, stage, T stage, and grade. To gain further insight into the clinical applicability and utility of mitophagyscore, the prognostic nomogram, a feasible tool to predict the probability of survival at 1-, 3-, and 5-years in patients with HCC was constructed. The CMap database and GDSC database allowed for an in-depth exploration of the association between the mitophagyscore and clinical treatment. Therapeutic small molecules that can reverse the mitophagy phenotype were obtained from the CMap database; these could provide new treatment opportunities for HCC. Thus, further experiments are needed to verify these findings. Additionally, we found that sensitivity towards 21 anti-tumor drugs was closely related to the mitophagyscore and this opened up promising perspectives for its potential clinical applicability, such as in the optimization of personalized therapy. Therefore, mitophagyscore not only functions as an independent prognostic tool to predict prognosis, but also triggers further thinking about the relationship between mitophagy and therapeutic agents for HCC. However, all the above results are based on bioinformatic mining of TCGA and ICGC databases, and more molecular biology-based experiments are required for their validation.

Due to the Spearman correlation analysis and the differential SPP1 expression across mitophagy subtypes, SPP1 may be an underlying reason for the mitophagy subtype specificity in HCC. Moreover, SPP1 may be a potential biomarker for the diagnosis and prognosis of HCC. SPP1 has previously been reported in the regulation of UPR- and ER stress-induced autophagy through intracellular S1P homeostasis (Lépine et al., 2011). However, the significance of SPP1 in association with mitophagy remains unclear. Our present study offered a fresh perspective on the mitophagic heterogeneity underlying HCC and identified a key gene, SPP1, which may contribute to the formation of three different mitophagy subtypes. The findings laid the groundwork for further exploration of mitophagy heterogeneity in HCC. However, further experimentation is required to verify these findings.

Our research provides new ideas and materials for the personalized clinical treatment plans for patients with HCC, although some limitations of this study should be acknowledged. Firstly, our study only included a bioinformatics analysis, lacking the validation of solid clinical specimens. Additionally, the research was conducted with a retrospective design rather than a prospective one. However, the present study was conducted in two independent cohorts, therefore, the result is still reliable and acceptable. Thus, future studies with prospective clinical trials and mechanistic exploration are warranted to further validate the present result.

## Conclusion

In conclusion, using 26 mitophagy regulators, unsupervised clustering was performed to examine the mitophagy heterogeneity in HCC. The differential expression patterns of mitophagy regulators showed three distinct subtypes in HCC with extremely different mitophagy accumulation levels. Furthermore, different mitophagy subtypes showed distinct metabolic patterns and prognoses. The high mitophagy subtype had the poorest prognosis and highest glycolytic metabolism. Additionally, we constructed and validated a novel mitophagy-associated risk score system, which may provide a potential prognostic predictor for HCC. Moreover, based on the Cmap database, our results provided a range of small molecule compounds, which may ultimately pave the way for the implementation of targeted risk model-related treatments for HCC patients. We also demonstrated the difference in sensitivity towards potential anti-HCC drugs across the clusters and the findings were corroborated by previous research on mitophagy-associated risk phenotypes.

## Data Availability

Publicly available datasets were analyzed in this study. This data can be found in The Cancer Genome Atlas (TCGA) database (https://portal.gdc.cancer.gov/), Gene Expression Omnibus (GEO) database (https://www.ncbi.nlm.nih.gov/geo/) and International Cancer Genome Consortium (ICGC) data portal (https://icgc.org/).

## References

[B1] Atwa S. M. Odenthal M. M. El Tayebi H. (2021). Genetic Heterogeneity, Therapeutic Hurdle Confronting Sorafenib and Immune Checkpoint Inhibitors in Hepatocellular Carcinoma. Cancers 13, 4343. 10.3390/cancers13174343 34503153 PMC8430643

[B2] Beal E. W. Tumin D. Kabir A. Moris D. Zhang X.-F. Chakedis J. (2017). Trends in the Mortality of Hepatocellular Carcinoma in the United States. J. Gastrointest. Surg. 21, 2033–2038. 10.1007/s11605-017-3526-7 28785936

[B3] Borgia M. Dal Bo M. Toffoli G. (2021). Role of Virus-Related Chronic Inflammation and Mechanisms of Cancer Immune-Suppression in Pathogenesis and Progression of Hepatocellular Carcinoma. Cancers 13, 4387. 10.3390/cancers13174387 34503196 PMC8431318

[B4] Calcinotto A. Filipazzi P. Grioni M. Iero M. De Milito A. Ricupito A. (2012). Modulation of Microenvironment Acidity Reverses Anergy in Human and Murine Tumor-Infiltrating T Lymphocytes. Cancer Res. 72, 2746–2756. 10.1158/0008-5472.CAN-11-1272 22593198

[B5] Chen Y. Dorn G. W. 2nd (2013). PINK1-phosphorylated Mitofusin 2 Is a Parkin Receptor for Culling Damaged Mitochondria. Science 340, 471–475. 10.1126/science.1231031 23620051 PMC3774525

[B6] Ding C. Shan Z. Li M. Chen H. Li X. Jin Z. (2021). Characterization of the Fatty Acid Metabolism in Colorectal Cancer to Guide Clinical Therapy. Mol. Ther. Oncolytics 20, 532–544. 10.1016/j.omto.2021.02.010 33738339 PMC7941088

[B7] Durcan T. M. Fon E. A. (2015). The Three 'P's of Mitophagy: PARKIN, PINK1, and post-translational Modifications. Genes Dev. 29 (10), 989–999. 10.1101/gad.262758.115 25995186 PMC4441056

[B8] Ferro F. Servais S. Besson P. Roger S. Dumas J.-F. Brisson L. (2020). Autophagy and Mitophagy in Cancer Metabolic Remodelling. Semin. Cel Dev. Biol. 98, 129–138. 10.1016/j.semcdb.2019.05.029 31154012

[B9] Geisler S. Holmström K. M. Skujat D. Fiesel F. C. Rothfuss O. C. Kahle P. J. (2010). PINK1/Parkin-mediated Mitophagy Is Dependent on VDAC1 and p62/SQSTM1. Nat. Cel Biol 12 (2), 119–131. 10.1038/ncb2012 20098416

[B10] Glauser L. Sonnay S. Stafa K. Moore D. J. (2011). Parkin Promotes the Ubiquitination and Degradation of the Mitochondrial Fusion Factor Mitofusin 1. J. Neurochem. 118 (4), 636–645. 10.1111/j.1471-4159.2011.07318.x 21615408

[B11] Goetze K. Walenta S. Ksiazkiewicz M. Kunz-Schughart L. A. Mueller-Klieser W. (2011). Lactate Enhances Motility of Tumor Cells and Inhibits Monocyte Migration and Cytokine Release. Int. J. Oncol. 39 (2), 453–463. 10.3892/ijo.2011.1055 21617859

[B12] Hänzelmann S. Castelo R. Guinney J. (2013). GSVA: Gene Set Variation Analysis for Microarray and RNA-Seq Data. BMC bioinformatics 14. 10.1186/1471-2105-14-7 PMC3618321 23323831

[B13] Huang B. Song B.-l. Xu C. (2020). Cholesterol Metabolism in Cancer: Mechanisms and Therapeutic Opportunities. Nat. Metab. 2 (2), 132–141. 10.1038/s42255-020-0174-0 32694690

[B14] Huang Z. Tong Y. Tian H. Zhao C. (2020). Establishment of a Prognostic Nomogram for Lung Adenocarcinoma with Brain Metastases. World Neurosurg. 141, e700–e709. 10.1016/j.wneu.2020.05.273 32531436

[B15] Jin S. M. Lazarou M. Wang C. Kane L. A. Narendra D. P. Youle R. J. (2010). Mitochondrial Membrane Potential Regulates PINK1 Import and Proteolytic Destabilization by PARL. J. Cel. Biol. 191 (5), 933–942. 10.1083/jcb.201008084 PMC2995166 21115803

[B16] Koh H. Park G.-S. Shin S.-M. Park C. E. Kim S. Han S. J. (2018). Mitochondrial Mutations in Cholestatic Liver Disease with Biliary Atresia. Sci. Rep. 8, 1. 10.1038/s41598-017-18958-8 29343773 PMC5772057

[B17] Lapidot T. Sirard C. Vormoor J. Murdoch B. Hoang T. Caceres-Cortes J. (1994). A Cell Initiating Human Acute Myeloid Leukaemia after Transplantation into SCID Mice. Nature 367, 645–648. 10.1038/367645a0 7509044

[B18] Lardner A. (2001). The Effects of Extracellular pH on Immune Function. J. Leukoc. Biol. 69 (4), 522–530. 11310837

[B19] Lazarou M. Sliter D. A. Kane L. A. Sarraf S. A. Wang C. Burman J. L. (2015). The Ubiquitin Kinase PINK1 Recruits Autophagy Receptors to Induce Mitophagy. Nature 524, 309–314. 10.1038/nature14893 26266977 PMC5018156

[B20] Lépine S. Allegood J. C. Park M. Dent P. Milstien S. Spiegel S. (2011). Sphingosine-1-phosphate Phosphohydrolase-1 Regulates ER Stress-Induced Autophagy. Cell Death Differ 18 (2), 350–361. 10.1038/cdd.2010.104 20798685 PMC3131882

[B21] Li L. Wang H. (2016). Heterogeneity of Liver Cancer and Personalized Therapy. Cancer Lett. 379 (2), 191–197. 10.1016/j.canlet.2015.07.018 26213370

[B22] Liu J. Feng M. Li S. Nie S. Wang H. Wu S. (2020). Identification of Molecular Markers Associated with the Progression and Prognosis of Endometrial Cancer: a Bioinformatic Study. Cancer Cel Int 20, 59. 10.1186/s12935-020-1140-3 PMC7031962 32099532

[B23] Liu K. Lee J. Kim J. Y. Wang L. Tian Y. Chan S. T. (2017). Mitophagy Controls the Activities of Tumor Suppressor P53 to Regulate Hepatic Cancer Stem Cells. Mol. Cel. 68 (2), 281–292. 10.1016/j.molcel.2017.09.022 PMC5687282 29033320

[B24] Lopez Domenech G. Covill‐Cooke C. Ivankovic D. Halff E. F. Sheehan D. F. Norkett R. (2018). Miro Proteins Coordinate Microtubule‐ and Actin‐dependent Mitochondrial Transport and Distribution. Embo J. 37 (3), 321–336. 10.15252/embj.201696380 29311115 PMC5793800

[B25] Malta T. M. Sokolov A. Gentles A. J. Burzykowski T. Poisson L. Weinstein J. N. (2018). Machine Learning Identifies Stemness Features Associated with Oncogenic Dedifferentiation. Cell 173, 338–e15. 10.1016/j.cell.2018.03.034 29625051 PMC5902191

[B26] Pei J. Wang Y. Li Y. (2020). Identification of Key Genes Controlling Breast Cancer Stem Cell Characteristics via Stemness Indices Analysis. J. Transl Med. 18, 74. 10.1186/s12967-020-02260-9 32050983 PMC7014665

[B27] Roca-Agujetas V. de Dios C. Abadin X. Colell A. (2021). Upregulation of Brain Cholesterol Levels Inhibits Mitophagy in Alzheimer Disease. Autophagy 17 (6), 1555–1557. 10.1080/15548627.2021.1920814 33945386 PMC8204952

[B28] Schieber M. Chandel N. S. (2014). ROS Function in Redox Signaling and Oxidative Stress. Curr. Biol. 24, R453–R462. 10.1016/j.cub.2014.03.034 24845678 PMC4055301

[B29] Severi T. van Malenstein H. Verslype C. van Pelt J. F. (2010). Tumor Initiation and Progression in Hepatocellular Carcinoma: Risk Factors, Classification, and Therapeutic Targets. Acta Pharmacol. Sin 31, 1409–1420. 10.1038/aps.2010.142 20953207 PMC4003336

[B30] Shen Y. Liu J. Zhang L. Dong S. Zhang J. Liu Y. (2019). Identification of Potential Biomarkers and Survival Analysis for Head and Neck Squamous Cell Carcinoma Using Bioinformatics Strategy: A Study Based on TCGA and GEO Datasets. Biomed. Research International 2019, 1–14. 10.1155/2019/7376034 PMC6702813 31485443

[B31] Sliter D. A. Martinez J. Hao L. Chen X. Sun N. Fischer T. D. (2018). Parkin and PINK1 Mitigate STING-Induced Inflammation. Nature 561, 258–262. 10.1038/s41586-018-0448-9 30135585 PMC7362342

[B32] Song X. Du R. Gui H. Zhou M. Zhong W. Mao C. (2020). Identification of Potential Hub Genes Related to the Progression and Prognosis of Hepatocellular Carcinoma through Integrated Bioinformatics Analysis. Oncol. Rep. 43 (1), 133–146. 10.3892/or.2019.7400 31746405 PMC6908929

[B33] Sotiriou C. Wirapati P. Loi S. Harris A. Fox S. Smeds J. (2006). Gene Expression Profiling in Breast Cancer: Understanding the Molecular Basis of Histologic Grade to Improve Prognosis. J. Natl. Cancer Inst. 98 (4), 262–272. 10.1093/jnci/djj052 16478745

[B34] Stern R. (2008). Hyaluronidases in Cancer Biology. Semin. Cancer Biol. 18, 275–280. 10.1016/j.semcancer.2008.03.017 18485730

[B35] Tang Z. Li C. Kang B. Gao G. Li C. Zhang Z. (2017). GEPIA: a Web Server for Cancer and normal Gene Expression Profiling and Interactive Analyses. Nucleic Acids Res. 45, W98–W102. 10.1093/nar/gkx247 28407145 PMC5570223

[B36] Vara-Perez M. Felipe-Abrio B. Agostinis P. (2019). Mitophagy in Cancer: A Tale of Adaptation. Cells 8, 493. 10.3390/cells8050493 PMC6562743 31121959

[B37] Walenta S. Mueller-Klieser W. F. (2004). Lactate: Mirror and Motor of Tumor Malignancy. Semin. Radiat. Oncol. 14 (3), 267–274. 10.1016/j.semradonc.2004.04.004 15254870

[B38] Wang X. Winter D. Ashrafi G. Schlehe J. Wong Y. L. Selkoe D. (2011). PINK1 and Parkin Target Miro for Phosphorylation and Degradation to Arrest Mitochondrial Motility. Cell 147, 893–906. 10.1016/j.cell.2011.10.018 22078885 PMC3261796

[B39] Wilkerson M. D. Hayes D. N. (2010). ConsensusClusterPlus: a Class Discovery Tool with Confidence Assessments and Item Tracking. Bioinformatics (Oxford, England) 26, 1572–1573. 10.1093/bioinformatics/btq170 PMC2881355 20427518

[B40] Wong Y. C. Holzbaur E. L. F. (2014). Optineurin Is an Autophagy Receptor for Damaged Mitochondria in Parkin-Mediated Mitophagy that Is Disrupted by an ALS-Linked Mutation. Proc. Natl. Acad. Sci. USA 111, E4439–E4448. 10.1073/pnas.1405752111 25294927 PMC4210283

[B41] Xu M. Li Y. Li W. Zhao Q. Zhang Q. Le K. (2020). Immune and Stroma Related Genes in Breast Cancer: A Comprehensive Analysis of Tumor Microenvironment Based on the Cancer Genome Atlas (TCGA) Database. Front. Med. 7, 64. 10.3389/fmed.2020.00064 PMC7066229 32195260

[B42] Zeng D. Li M. Zhou R. Zhang J. Sun H. Shi M. (2019). Tumor Microenvironment Characterization in Gastric Cancer Identifies Prognostic and Immunotherapeutically Relevant Gene Signatures. Cancer Immunol. Res. 7 (5), 737–750. 10.1158/2326-6066.CIR-18-0436 30842092

[B43] Zhou R. Yazdi A. S. Menu P. Tschopp J. (2011). A Role for Mitochondria in NLRP3 Inflammasome Activation. Nature 469, 221–225. 10.1038/nature09663 21124315

[B44] Zhu C. Pan Y. Ma S. Cao K. Zhou S. Zhao A. (2018). Therapeutic Approaches Targeting Cancer Stem Cells. J. Can. Res. Ther. 14 (7), 1469–1475. 10.4103/jcrt.JCRT_976_17 30589025

[B45] Zoni E. Minoli M. Bovet C. Wehrhan A. Piscuoglio S. Ng C. K. Y. (2019). Preoperative Plasma Fatty Acid Metabolites Inform Risk of Prostate Cancer Progression and May Be Used for Personalized Patient Stratification. BMC cancer 19, 1. 10.1186/s12885-019-6418-2 31842810 PMC6916032

