# Supplementary figures and images for: Identification of HCC Subtypes With Different Prognosis and Metabolic Patterns Based on Mitophagy
Source: Front Cell Dev Biol. 2021 Dec 16;9:799507. doi: 10.3389/fcell.2021.799507 (PMC8716756; doi:10.3389/fcell.2021.799507)

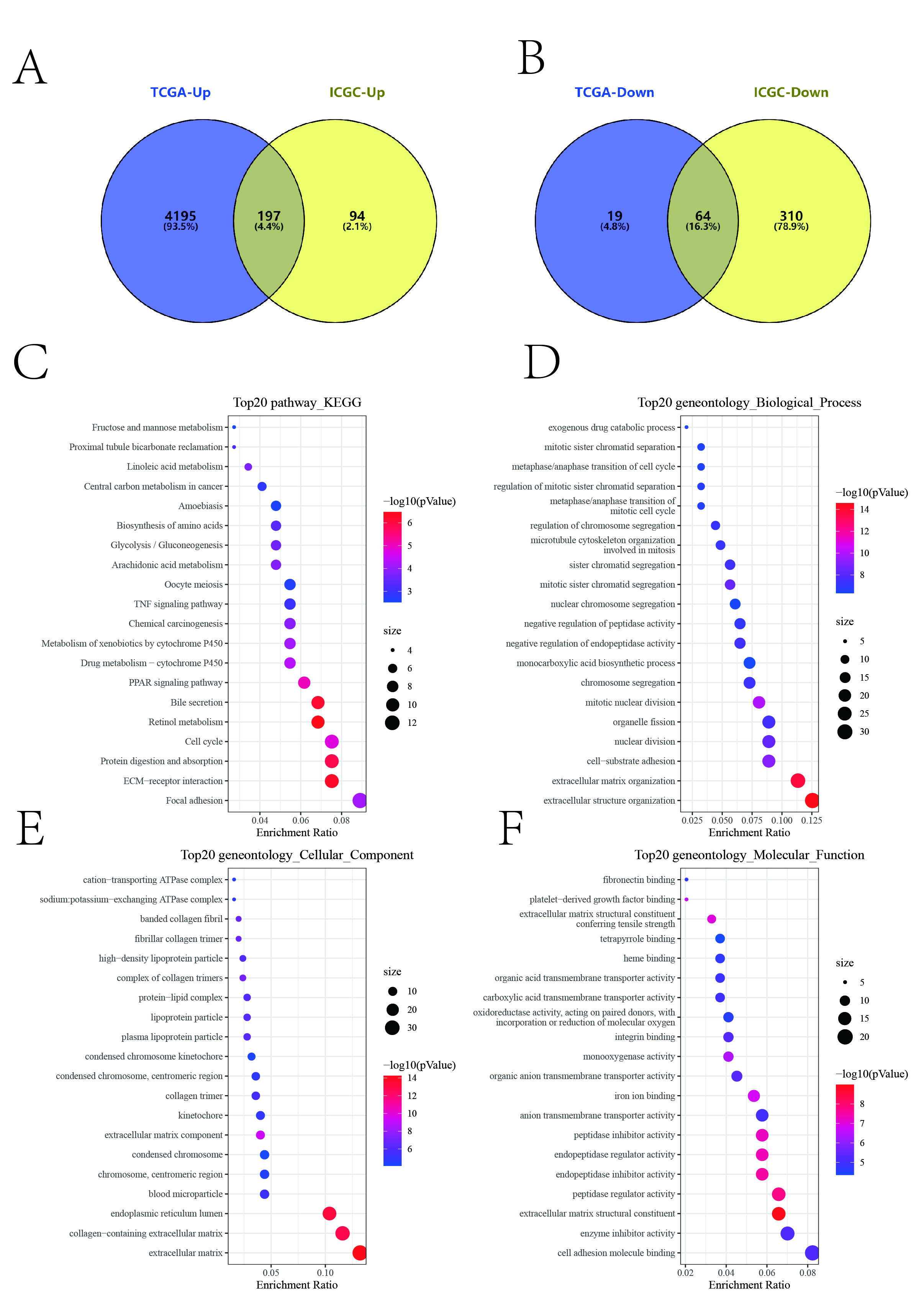

Supplement: Supplementary file 1 [file Image3.JPEG]

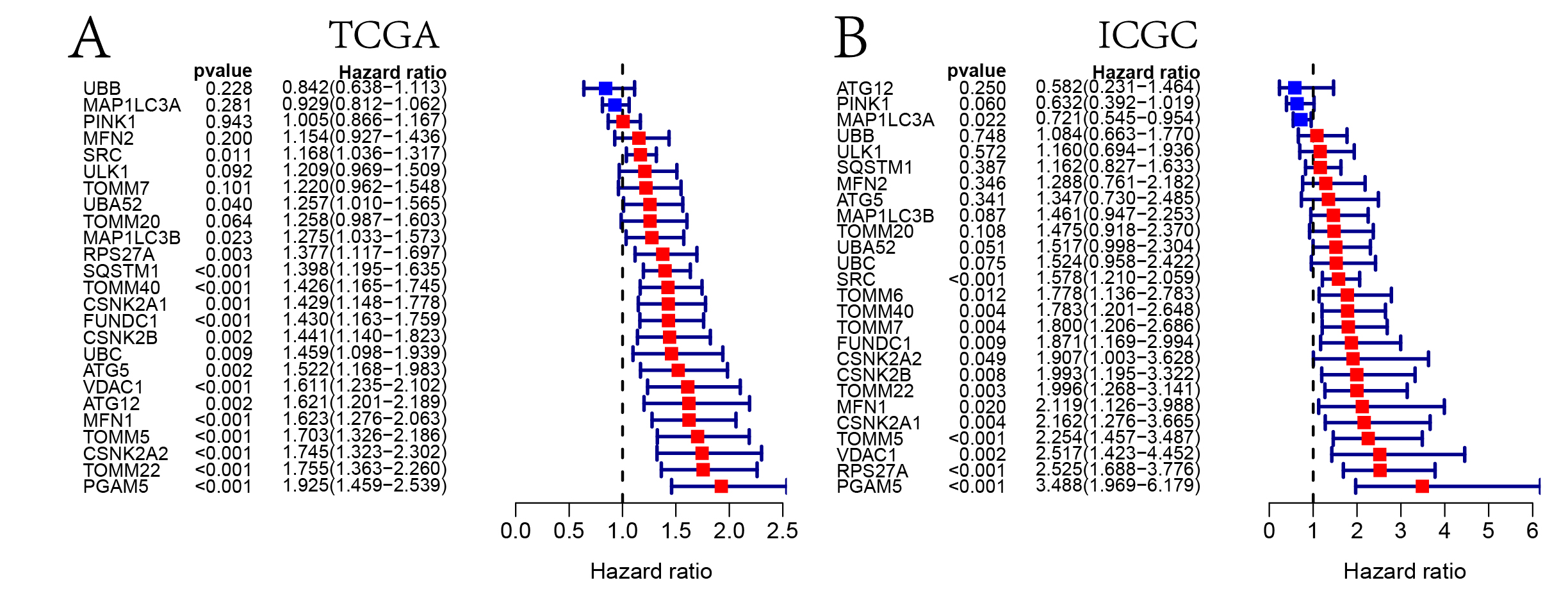

Supplement: Supplementary file 3 [file Image1.JPEG]

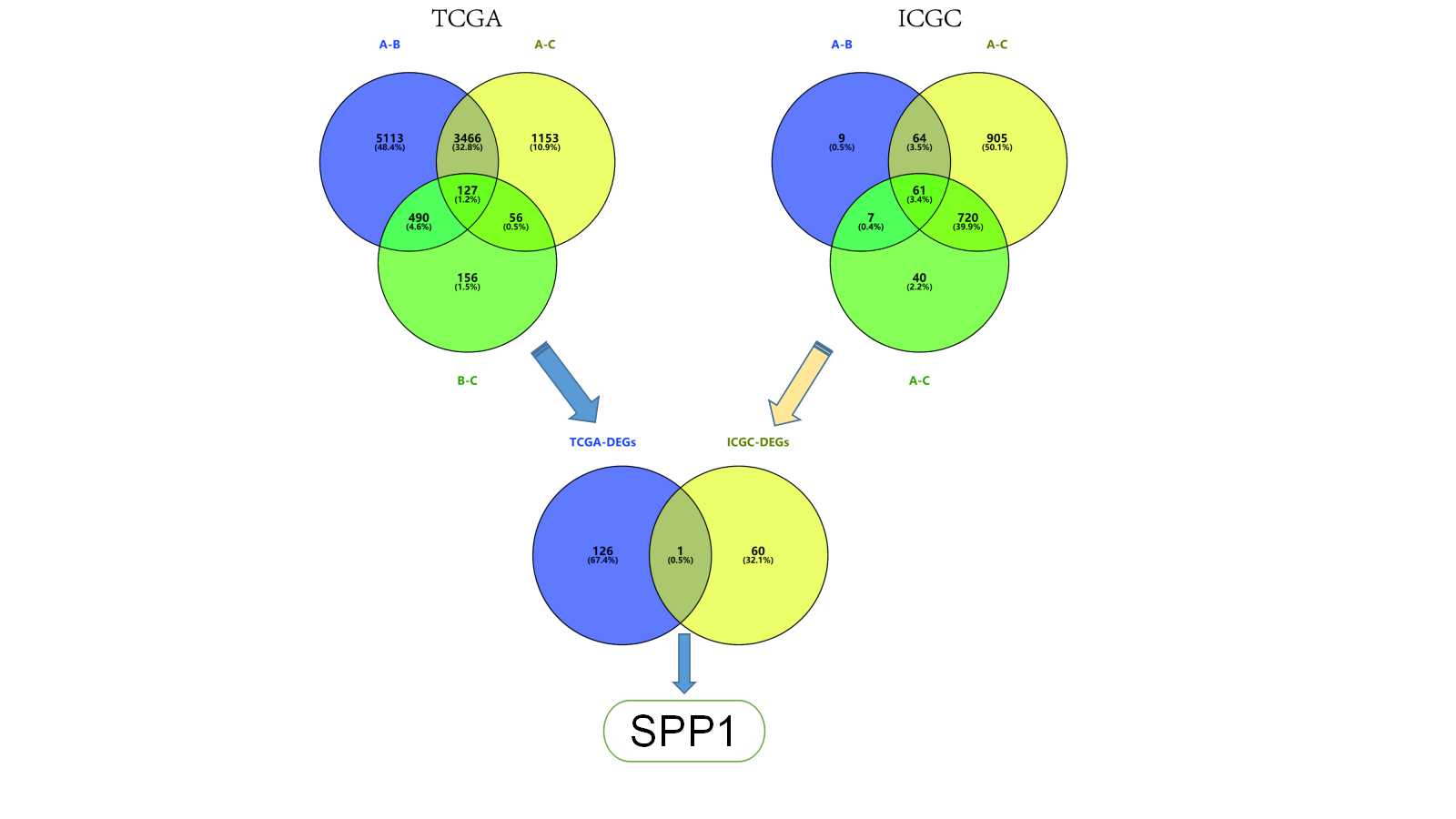

Supplement: Supplementary file 4 [file Image4.JPEG]

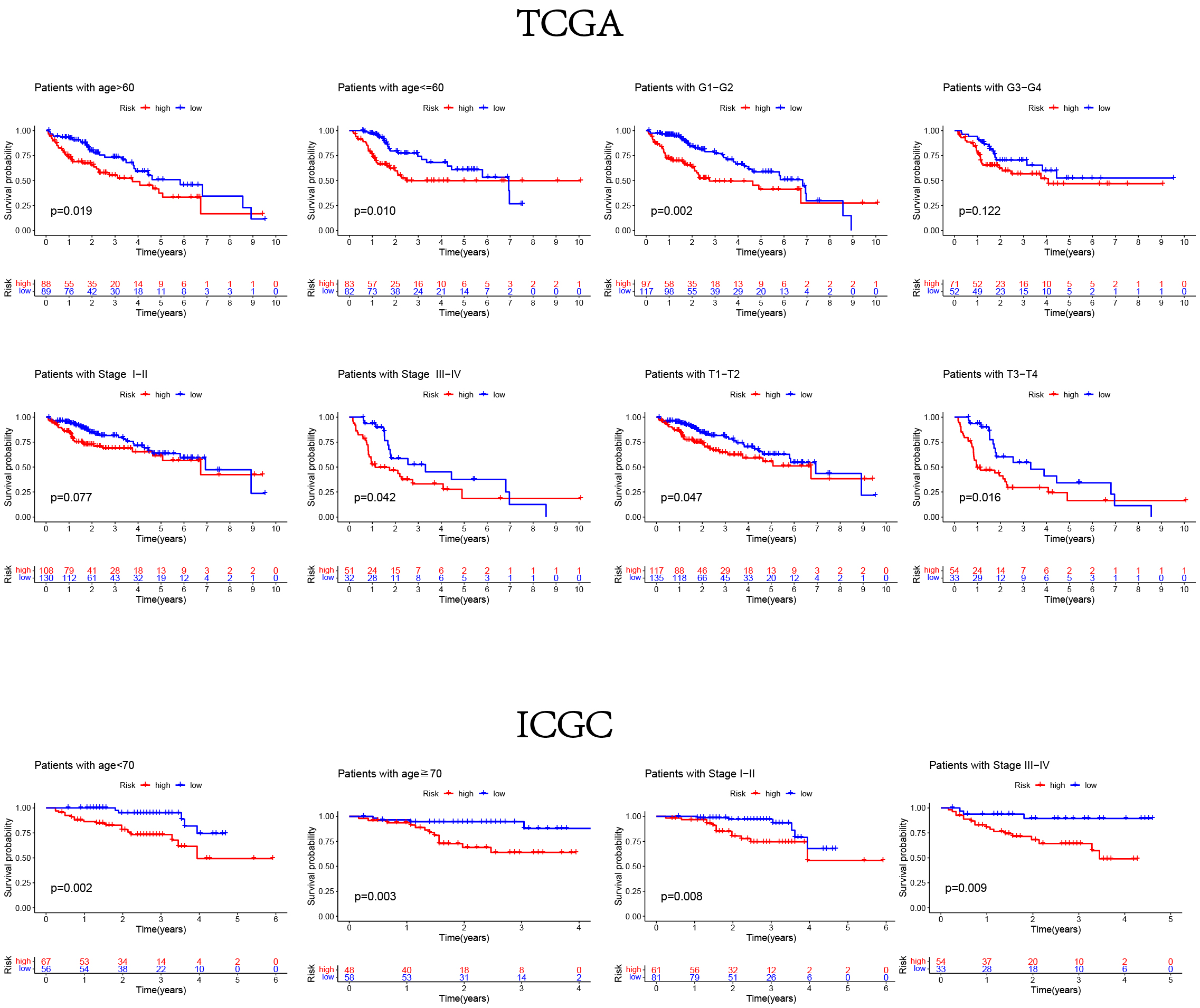

Supplement: Supplementary file 5 [file Image2.JPEG]

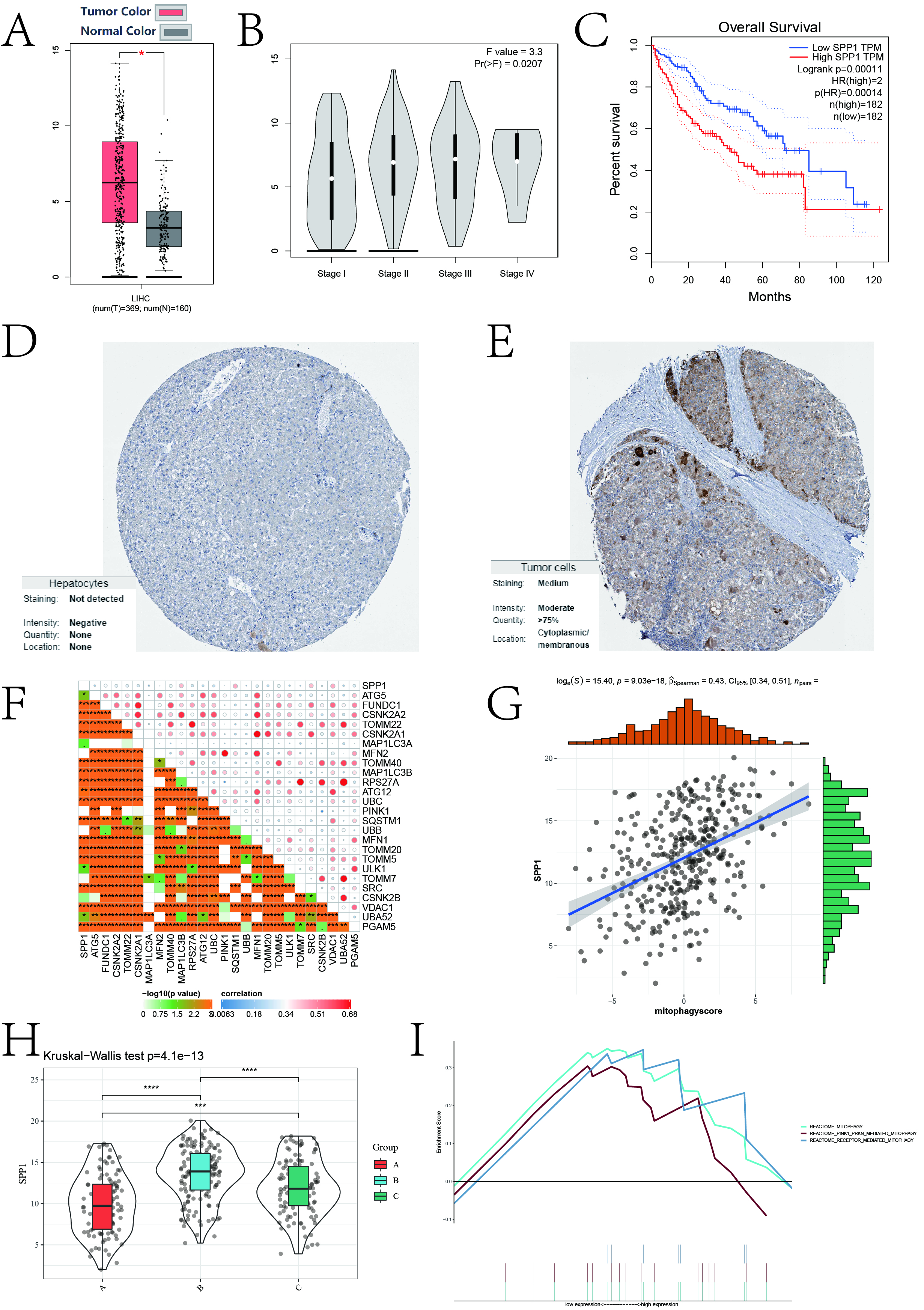

Supplement: Supplementary file 6 [file Image5.JPEG]
