# Supplementary material for: Identification of HCC Subtypes With Different Prognosis and Metabolic Patterns Based on Mitophagy
Source: Front Cell Dev Biol. 2021 Dec 16;9:799507. doi: 10.3389/fcell.2021.799507 (PMC8716756; doi:10.3389/fcell.2021.799507)
Supplement: Supplementary file 2 [file Table1.DOCX]

Supplementary Table 1： Signaling pathway associated with mitophagy,cholesterol biosynthesis and glycolysis.

| **Exact source** | **Brief description** | **Gene** |
| --- | --- | --- |
| R-HSA-5205647 | REACTOME MITOPHAGY | ATG12 ATG5 CSNK2A1 CSNK2A2 CSNK2B FUNDC1 MAP1LC3A MAP1LC3B MFN1 MFN2 MTERF3 PGAM5 PINK1 PRKN RPS27A SQSTM1 SRC TOMM20 TOMM22 TOMM40 TOMM5 TOMM6 TOMM7 TOMM70 UBA52 UBB UBC ULK1 VDAC1 |
| R-HSA-5205685 | REACTOME PINK1 PRKN MEDIATED MITOPHAGY | ATG12 ATG5 MAP1LC3A MAP1LC3B MFN1 MFN2 MTERF3 PINK1 PRKN RPS27A SQSTM1 TOMM20 TOMM22 TOMM40 TOMM5 TOMM6 TOMM7 TOMM70 UBA52 UBB UBC VDAC1 |
| R-HSA-8934903 | REACTOME RECEPTOR MEDIATED MITOPHAGY | ATG12 ATG5 CSNK2A1 CSNK2A2 CSNK2B FUNDC1 MAP1LC3A MAP1LC3B PGAM5 SRC ULK1 |
| R-HSA-191273 | REACTOME CHOLESTEROL BIOSYNTHESIS | ACAT2 ARV1 CYP51A1 DHCR24 DHCR7 EBP FDFT1 FDPS GGPS1 HMGCR HMGCS1 HSD17B7 IDI1 IDI2 LBR LSS MSMO1 MVD MVK NSDHL PLPP6 PMVK SC5D SQLE TM7SF2 |
| R-HSA-70171 | REACTOME GLYCOLYSIS | AAAS ADPGK ALDOA ALDOB ALDOC BPGM ENO1 ENO2 ENO3 GAPDH GAPDHS GCK GCKR GNPDA1 GNPDA2 GPI HK1 HK2 HK3 NDC1 NUP107 NUP133 NUP153 NUP155 NUP160 NUP188 NUP205 NUP210 NUP214 NUP35 NUP37 NUP42 NUP43 NUP50 NUP54 NUP58 NUP62 NUP85 NUP88 NUP93 NUP98 PFKFB1 PFKFB2 PFKFB3 PFKFB4 PFKL PFKM PFKP PGAM1 PGAM2 PGK1 PGK2 PGM2L1 PGP PKLR PKM POM121 POM121C PPP2CA PPP2CB PPP2R1A PPP2R1B PPP2R5D PRKACA PRKACB PRKACG RAE1 RANBP2 SEC13 SEH1L TPI1 TPR |
