# Supplementary material for: Identification of HCC Subtypes With Different Prognosis and Metabolic Patterns Based on Mitophagy
Source: Front Cell Dev Biol. 2021 Dec 16;9:799507. doi: 10.3389/fcell.2021.799507 (PMC8716756; doi:10.3389/fcell.2021.799507)
Supplement: Supplementary file 7 [file Table2.DOCX]

Supplementary Table 2： Enrichment scores of mitophagy,cholesterol biosynthesis and glycolysis related gene sets by ssGSEA.

| id | Data Set | cluster(mitophagy) | cluster(metabolism) | mitophagy | pink1 prkn mediated mitophagy | receptor mediated mitophagy | cholesterol biosynthesis | GLYCOLYSIS |
| --- | --- | --- | --- | --- | --- | --- | --- | --- |
| TCGA-G3-A3CI | TCGA | A | A | 0.694318401 | 0.791175631 | 0.373612523 | 0.911179166 | 0.511990746 |
| TCGA-CC-A8HT | TCGA | B | A | 0.744510577 | 0.769065633 | 0.584263294 | 0.721844103 | 0.742051036 |
| TCGA-XR-A8TD | TCGA | C | A | 0.702429114 | 0.83688854 | 0.410559987 | 0.746318816 | 0.791108747 |
| TCGA-EP-A26S | TCGA | B | A | 0.655137381 | 0.81613801 | 0.24816212 | 0.776407346 | 0.32854774 |
| TCGA-DD-A39V | TCGA | A | B | 0.803474858 | 0.924153464 | 0.487700259 | 0.575481567 | 0.283316334 |
| TCGA-DD-AADP | TCGA | A | A | 0.580250466 | 0.671077353 | 0.168357391 | 0.821506473 | 0.374150281 |
| TCGA-BC-A5W4 | TCGA | A | B | 0.628256331 | 0.78529334 | 0.196034825 | 0.897794099 | 0.295217241 |
| TCGA-RC-A7SK | TCGA | C | B | 0.691399181 | 0.797867382 | 0.376189207 | 0.659561148 | 0.243308765 |
| TCGA-DD-AADA | TCGA | A | B | 0.655737608 | 0.702528191 | 0.472335519 | 0.616128471 | 0.358714186 |
| TCGA-DD-AAEG | TCGA | B | A | 0.708079685 | 0.800290543 | 0.262541311 | 0.786809041 | 0.354942769 |
| TCGA-2Y-A9GZ | TCGA | C | A | 0.579729832 | 0.677418194 | 0.131452716 | 0.758439722 | 0.389627533 |
| TCGA-DD-A1EC | TCGA | B | C | 0.673171492 | 0.721993505 | 0.342852124 | 0.140222399 | 0.461610522 |
| TCGA-DD-AACL | TCGA | A | C | 0.77484852 | 0.782583262 | 0.50729658 | 0.736192365 | 0.75012225 |
| TCGA-BD-A3ER | TCGA | C | B | 0.668965377 | 0.740177596 | 0.352309056 | 0.683966518 | 0.570596803 |
| TCGA-2Y-A9HA | TCGA | B | A | 0.791045005 | 0.902360263 | 0.458959714 | 0.840470449 | 0.540056668 |
| TCGA-2Y-A9H6 | TCGA | C | B | 0.691107121 | 0.763397303 | 0.484577211 | 0.497289107 | 0.325215464 |
| TCGA-DD-AAD1 | TCGA | C | A | 0.594706088 | 0.679274331 | 0.222083232 | 0.774436751 | 0.295792424 |
| TCGA-EP-A3JL | TCGA | B | A | 0.760484165 | 0.814733705 | 0.471674548 | 0.810830909 | 0.335857995 |
| TCGA-DD-AADD | TCGA | C | A | 0.789995475 | 0.817721208 | 0.521959393 | 0.974033798 | 0.623607023 |
| TCGA-DD-AADK | TCGA | B | A | 0.655059808 | 0.700637194 | 0.256489631 | 0.749093813 | 0.278649026 |
| TCGA-G3-AAV1 | TCGA | B | A | 0.78386603 | 0.813664963 | 0.550904153 | 0.707124134 | 0.505216183 |
| TCGA-DD-AAE9 | TCGA | A | B | 0.771763422 | 0.801646331 | 0.50201798 | 0.716396597 | 0.174886072 |
| TCGA-CC-A7IE | TCGA | B | C | 0.810837688 | 0.815258129 | 0.689115583 | 0.629998649 | 0.606897851 |
| TCGA-DD-A1EA | TCGA | C | A | 0.668748701 | 0.777168959 | 0.292767387 | 0.766575543 | 0.486585073 |
| TCGA-DD-A39Z | TCGA | A | B | 0.631644367 | 0.828004643 | 0.020685548 | 0.845383341 | 0.352868724 |
| TCGA-DD-A115 | TCGA | A | B | 0.608794523 | 0.711588001 | 0.210475577 | 0.691364871 | 0.325355938 |
| TCGA-FV-A4ZQ | TCGA | C | A | 0.726907277 | 0.756763474 | 0.428171747 | 0.861092501 | 0.595560178 |
| TCGA-DD-A118 | TCGA | B | A | 0.843826119 | 0.892193137 | 0.618039269 | 0.685070735 | 0.29540422 |
| TCGA-G3-A3CJ | TCGA | A | B | 0.717676042 | 0.888065661 | 0.285416561 | 0.965638338 | 0.311328743 |
| TCGA-DD-A4NH | TCGA | C | C | 0.612745048 | 0.599319413 | 0.52381535 | 0.656570104 | 0.63687143 |
| TCGA-FV-A3R3 | TCGA | C | C | 0.709166802 | 0.770652227 | 0.511633442 | 0.311414771 | 0.289783813 |
| TCGA-DD-AADL | TCGA | C | A | 0.64464645 | 0.75079493 | 0.189607459 | 0.764917611 | 0.542174363 |
| TCGA-DD-A1EL | TCGA | B | A | 0.855035402 | 0.940221634 | 0.48703235 | 0.7273932 | 0.619477264 |
| TCGA-DD-AACZ | TCGA | B | A | 0.874150301 | 0.883128636 | 0.691441098 | 0.792072506 | 0.802037024 |
| TCGA-UB-A7MD | TCGA | C | A | 0.805424111 | 0.911637308 | 0.517614096 | 0.593299897 | 0.403780701 |
| TCGA-RC-A7S9 | TCGA | B | B | 0.618007325 | 0.699392994 | 0.347950059 | 0.598988595 | 0.372608648 |
| TCGA-FV-A496 | TCGA | C | A | 0.717231005 | 0.855180352 | 0.37610809 | 0.892779362 | 0.569880814 |
| TCGA-2Y-A9H9 | TCGA | A | A | 0.639735241 | 0.816194787 | 0.238510508 | 0.889382361 | 0.383167907 |
| TCGA-ED-A7PX | TCGA | B | C | 0.830449789 | 0.809091948 | 0.704328973 | 0.484918045 | 0.483992838 |
| TCGA-CC-A7IG | TCGA | B | C | 0.689913109 | 0.748865481 | 0.345098416 | 0.657695709 | 0.414434038 |
| TCGA-DD-A73G | TCGA | B | B | 0.796099949 | 0.819391362 | 0.673381323 | 0.84095203 | 0.199272254 |
| TCGA-BC-A10Z | TCGA | C | A | 0.652284432 | 0.765014024 | 0.363861897 | 0.89084406 | 0.46748909 |
| TCGA-CC-A7IJ | TCGA | B | C | 0.691036274 | 0.731994125 | 0.442145122 | 0.281709496 | 0.566210724 |
| TCGA-DD-AACE | TCGA | A | A | 0.671390904 | 0.837164701 | 0.291228042 | 0.86474551 | 0.636628209 |
| TCGA-DD-A4NG | TCGA | C | A | 0.71507313 | 0.713217707 | 0.44926288 | 0.674981225 | 0.497050706 |
| TCGA-DD-A3A5 | TCGA | A | A | 0.652268033 | 0.851097189 | 0.265817951 | 0.877733068 | 0.346625269 |
| TCGA-DD-A73F | TCGA | C | A | 0.586853338 | 0.685358787 | 0.176506597 | 0.851162001 | 0.531863587 |
| TCGA-FV-A2QQ | TCGA | B | A | 0.751425231 | 0.817090286 | 0.450205976 | 0.734785738 | 0.402878955 |
| TCGA-UB-A7MF | TCGA | B | A | 0.715640093 | 0.759341925 | 0.496579697 | 0.829908725 | 0.56613333 |
| TCGA-ED-A7XP | TCGA | B | A | 0.729935142 | 0.791843984 | 0.441958323 | 0.516788665 | 0.332350746 |
| TCGA-DD-AADN | TCGA | A | B | 0.769050109 | 0.79600767 | 0.539207265 | 0.839296651 | 0.498549889 |
| TCGA-2Y-A9H4 | TCGA | A | B | 0.810964579 | 0.95510182 | 0.559755453 | 0.801250745 | 0.300908527 |
| TCGA-K7-A5RF | TCGA | A | B | 0.578494673 | 0.703073831 | 0.26572123 | 0.633374334 | 0.422153581 |
| TCGA-DD-AAEA | TCGA | A | B | 0.661312632 | 0.782069515 | 0.409147271 | 0.96379823 | 0.594287989 |
| TCGA-ZS-A9CG | TCGA | C | B | 0.775743349 | 0.886420182 | 0.453878952 | 0.766254007 | 0.188428334 |
| TCGA-WX-AA47 | TCGA | A | B | 0.767987505 | 0.875925398 | 0.500540823 | 0.495445481 | 0.293720754 |
| TCGA-ED-A459 | TCGA | B | C | 0.697954571 | 0.760528129 | 0.392346501 | 0.42384284 | 0.608655826 |
| TCGA-G3-AAUZ | TCGA | A | B | 0.631190908 | 0.753367521 | 0.263473025 | 0.775399867 | 0.228049005 |
| TCGA-BC-A216 | TCGA | C | A | 0.527481217 | 0.576016756 | 0.122099363 | 0.613059013 | 0.498052705 |
| TCGA-ED-A7XO | TCGA | A | A | 0.711272679 | 0.823578707 | 0.407407429 | 0.671400287 | 0.535714242 |
| TCGA-DD-AAEI | TCGA | A | A | 0.71705676 | 0.838528675 | 0.393017389 | 0.891984901 | 0.600912961 |
| TCGA-G3-A5SK | TCGA | A | B | 0.714337098 | 0.868582142 | 0.444099291 | 0.438003526 | 0.17892201 |
| TCGA-G3-A3CH | TCGA | C | A | 0.680613896 | 0.792569966 | 0.32652244 | 0.753204424 | 0.41443432 |
| TCGA-CC-A9FS | TCGA | A | B | 0.576370951 | 0.71734397 | 0.070285497 | 0.515473001 | 0.166141557 |
| TCGA-DD-A4NR | TCGA | B | A | 0.678807173 | 0.659082511 | 0.455585212 | 0.759286196 | 0.648718843 |
| TCGA-DD-AADI | TCGA | B | B | 0.661570889 | 0.718425392 | 0.353907952 | 0.470197665 | 0.1890781 |
| TCGA-CC-A5UC | TCGA | B | A | 0.812940197 | 0.818409113 | 0.583021242 | 0.920480304 | 0.652670367 |
| TCGA-DD-AADJ | TCGA | A | B | 0.788415806 | 0.8628038 | 0.562996012 | 0.86548355 | 0.330431148 |
| TCGA-RC-A7SB | TCGA | C | A | 0.704189046 | 0.880255651 | 0.355719117 | 0.854255286 | 0.398091053 |
| TCGA-DD-AADM | TCGA | A | B | 0.720235819 | 0.909175453 | 0.31567373 | 0.828418439 | 0.3201262 |
| TCGA-DD-A4ND | TCGA | C | A | 0.570712412 | 0.637031713 | 0.28270406 | 0.717160653 | 0.625777785 |
| TCGA-CC-5259 | TCGA | A | B | 0.795453685 | 0.800497206 | 0.755087828 | 0.729481616 | 0.258094922 |
| TCGA-ZS-A9CF | TCGA | C | A | 0.504819259 | 0.55191289 | 0.260403285 | 0.741489107 | 0.361033275 |
| TCGA-DD-AAE6 | TCGA | C | A | 0.787206659 | 0.798938078 | 0.51200726 | 0.816441715 | 0.515766573 |
| TCGA-DD-AACX | TCGA | B | A | 0.730102604 | 0.844797212 | 0.443389171 | 0.706146171 | 0.564124733 |
| TCGA-MR-A520 | TCGA | A | A | 0.742297525 | 0.861460376 | 0.391314565 | 0.959365796 | 0.519219208 |
| TCGA-BC-A110 | TCGA | A | B | 0.718779476 | 0.830768648 | 0.460595083 | 0.611199996 | 0.600120412 |
| TCGA-2Y-A9GT | TCGA | A | B | 0.567876561 | 0.710034178 | 0.248783819 | 0.553120829 | 0.241002807 |
| TCGA-ES-A2HT | TCGA | A | B | 0.753596152 | 0.907521837 | 0.390129803 | 0.771881975 | 0.317835107 |
| TCGA-BC-A10U | TCGA | C | C | 0.677118124 | 0.760571847 | 0.339888845 | 0.617297256 | 0.642079215 |
| TCGA-DD-A1EF | TCGA | C | B | 0.78854604 | 0.778148176 | 0.598600133 | 0.752969899 | 0.497311488 |
| TCGA-BC-A8YO | TCGA | B | A | 0.60952396 | 0.721862951 | 0.159837579 | 0.543012881 | 0.781529944 |
| TCGA-XR-A8TG | TCGA | C | A | 0.579354485 | 0.656199687 | 0.305429567 | 0.710718689 | 0.337636172 |
| TCGA-DD-AAC8 | TCGA | B | A | 0.779966883 | 0.846961523 | 0.440369932 | 0.813322799 | 0.411830613 |
| TCGA-FV-A4ZP | TCGA | B | B | 0.711517209 | 0.706301448 | 0.502752026 | 0.628932953 | 0.301596784 |
| TCGA-G3-A25T | TCGA | B | C | 0.778945576 | 0.736225675 | 0.649523432 | 0.424932174 | 0.217518417 |
| TCGA-DD-AAED | TCGA | C | B | 0.643015123 | 0.642826083 | 0.451082868 | 0.759058863 | 0.286562457 |
| TCGA-MI-A75I | TCGA | B | A | 0.713731335 | 0.778410932 | 0.291375942 | 0.56624911 | 0.540614108 |
| TCGA-DD-AACT | TCGA | A | B | 0.798318726 | 0.861385465 | 0.574834575 | 0.713520053 | 0.457002739 |
| TCGA-MI-A75C | TCGA | B | B | 0.807623571 | 0.844548514 | 0.507772669 | 0.734644387 | 0.39171848 |
| TCGA-CC-A3M9 | TCGA | B | C | 0.673980043 | 0.716473956 | 0.392312022 | 0.032798471 | 0.845082715 |
| TCGA-GJ-A6C0 | TCGA | B | C | 0.636359365 | 0.708744898 | 0.340609251 | 0.380836372 | 0.877230864 |
| TCGA-2Y-A9GX | TCGA | C | A | 0.617673422 | 0.718780303 | 0.331244482 | 0.542951526 | 0.485915687 |
| TCGA-DD-AAVX | TCGA | A | B | 0.671329369 | 0.811731524 | 0.189306459 | 0.542925371 | 0.381580829 |
| TCGA-5R-AA1C | TCGA | A | B | 0.755680176 | 0.887648183 | 0.385843431 | 0.694852385 | 0.265044123 |
| TCGA-DD-A4NB | TCGA | C | C | 0.709217843 | 0.785475145 | 0.497662741 | 0.447668377 | 0.331473683 |
| TCGA-G3-A25Z | TCGA | B | A | 0.754043719 | 0.880857484 | 0.402577842 | 0.887302893 | 0.468432271 |
| TCGA-BC-4072 | TCGA | B | C | 0.677908308 | 0.723032953 | 0.501134526 | 0.600155704 | 0.402148512 |
| TCGA-DD-A4NJ | TCGA | A | A | 0.464172583 | 0.565246485 | 0 | 0.691393206 | 0.689282351 |
| TCGA-CC-5262 | TCGA | B | A | 0.737293652 | 0.838663084 | 0.41936323 | 0.770225043 | 0.451710152 |
| TCGA-DD-AAVQ | TCGA | B | A | 0.578892274 | 0.631201225 | 0.30551606 | 0.796725978 | 0.342141538 |
| TCGA-BC-A10Q | TCGA | B | C | 0.695575366 | 0.666695759 | 0.464587309 | 0.140604661 | 0.321710038 |
| TCGA-MI-A75H | TCGA | A | B | 0.705427275 | 0.752290195 | 0.384468278 | 0.6904155 | 0.278378733 |
| TCGA-CC-A5UE | TCGA | B | A | 0.802340863 | 0.903924885 | 0.466883658 | 0.784918094 | 0.40780729 |
| TCGA-G3-A5SM | TCGA | B | A | 0.711484221 | 0.810716156 | 0.428724367 | 0.678973824 | 0.367391945 |
| TCGA-YA-A8S7 | TCGA | B | C | 0.758954549 | 0.781775456 | 0.573076995 | 0.337313539 | 0.392998836 |
| TCGA-ED-A5KG | TCGA | C | C | 0.526362794 | 0.573908963 | 0.274721456 | 0.461237399 | 0.623678894 |
| TCGA-FV-A23B | TCGA | B | A | 0.576844555 | 0.624255728 | 0.225738622 | 0.732531126 | 0.427574377 |
| TCGA-DD-A119 | TCGA | B | B | 0.75014001 | 0.85939715 | 0.453399594 | 0.894828302 | 0.29370274 |
| TCGA-DD-AADF | TCGA | A | A | 0.695724348 | 0.837636657 | 0.422364316 | 0.823705382 | 0.406816386 |
| TCGA-DD-A39X | TCGA | B | B | 0.717161277 | 0.745854779 | 0.429518289 | 0.748385574 | 0.221483753 |
| TCGA-DD-A4NS | TCGA | C | A | 0.53571604 | 0.615787154 | 0.195455866 | 0.564949439 | 0.387656942 |
| TCGA-UB-AA0V | TCGA | A | B | 0.633085645 | 0.7562681 | 0.288334983 | 0.603822119 | 0.423027197 |
| TCGA-BW-A5NO | TCGA | A | A | 0.618001732 | 0.738858457 | 0.295808345 | 0.903763444 | 0.453298062 |
| TCGA-DD-AAVY | TCGA | A | B | 0.645703235 | 0.779429335 | 0.144989021 | 0.85840984 | 0.409722316 |
| TCGA-DD-AAE3 | TCGA | A | B | 0.687981248 | 0.854136123 | 0.37459004 | 0.727115105 | 0.382251575 |
| TCGA-CC-A8HV | TCGA | B | A | 0.68508533 | 0.664352226 | 0.550084214 | 0.776558283 | 0.40273555 |
| TCGA-ZP-A9D0 | TCGA | C | B | 0.755406541 | 0.858089012 | 0.52126548 | 0.654958461 | 0.38995274 |
| TCGA-CC-A7IL | TCGA | B | A | 0.742935013 | 0.866876769 | 0.380631626 | 0.913758144 | 0.362232629 |
| TCGA-DD-A4NP | TCGA | C | B | 0.761472706 | 0.86572388 | 0.475954588 | 0.637727284 | 0.209721585 |
| TCGA-2Y-A9GW | TCGA | C | A | 0.68133066 | 0.773374772 | 0.354385802 | 0.638987618 | 0.509205634 |
| TCGA-G3-A3CG | TCGA | A | A | 0.673780777 | 0.79977195 | 0.286241013 | 0.707107022 | 0.467770088 |
| TCGA-DD-AAVV | TCGA | B | C | 0.831293552 | 0.900869038 | 0.569143624 | 0.600236486 | 0.421916287 |
| TCGA-UB-A7ME | TCGA | B | A | 0.599235554 | 0.628439999 | 0.358053458 | 0.833831653 | 0.275655609 |
| TCGA-G3-A5SJ | TCGA | B | C | 0.70292311 | 0.794061908 | 0.28671467 | 0.368666325 | 0.522779644 |
| TCGA-BW-A5NQ | TCGA | B | A | 0.743725504 | 0.851811908 | 0.367760811 | 0.723771016 | 0.623075714 |
| TCGA-RC-A6M5 | TCGA | B | C | 0.75307158 | 0.840616756 | 0.514109909 | 0.444987926 | 0.33408047 |
| TCGA-CC-5260 | TCGA | C | C | 0.547111518 | 0.542643491 | 0.195605803 | 0.231387833 | 0.505934189 |
| TCGA-2Y-A9GY | TCGA | B | C | 0.760923092 | 0.736050186 | 0.591472761 | 0.439494356 | 0.647608006 |
| TCGA-DD-AAVR | TCGA | B | B | 0.783732188 | 0.871926535 | 0.504514461 | 0.578921932 | 0.26289714 |
| TCGA-PD-A5DF | TCGA | C | A | 0.773657067 | 0.830773748 | 0.597400158 | 0.753692142 | 0.551375119 |
| TCGA-G3-A3CK | TCGA | C | A | 0.689901992 | 0.83661222 | 0.262748863 | 0.667385826 | 0.333866873 |
| TCGA-DD-A1EI | TCGA | B | A | 0.717378268 | 0.771244077 | 0.366347574 | 0.726410423 | 0.397214092 |
| TCGA-ZP-A9CV | TCGA | A | B | 0.66552419 | 0.809670999 | 0.277606006 | 0.767041217 | 0.404334545 |
| TCGA-BC-A10W | TCGA | B | A | 0.620765333 | 0.643740804 | 0.289530056 | 0.588508821 | 0.730532632 |
| TCGA-FV-A3R2 | TCGA | B | A | 0.7244492 | 0.828407422 | 0.301518251 | 0.848319428 | 0.378817838 |
| TCGA-FV-A3I1 | TCGA | C | A | 0.628664103 | 0.652342854 | 0.331716591 | 0.83762913 | 0.55806946 |
| TCGA-CC-A5UD | TCGA | B | C | 0.668754883 | 0.714525897 | 0.397169733 | 0.714739045 | 0.523582833 |
| TCGA-DD-AADV | TCGA | B | A | 0.620777857 | 0.732112068 | 0.178667371 | 0.605422393 | 0.39952332 |
| TCGA-ZP-A9D4 | TCGA | C | A | 0.76100769 | 0.828293093 | 0.5767446 | 0.700873166 | 0.337682347 |
| TCGA-DD-A4NA | TCGA | C | C | 0.695743434 | 0.68820223 | 0.544463145 | 0.347890877 | 0.4017386 |
| TCGA-FV-A495 | TCGA | A | B | 0.655103473 | 0.795012464 | 0.315998027 | 0.736419968 | 0.274859195 |
| TCGA-G3-A5SL | TCGA | C | A | 0.722312166 | 0.81957783 | 0.329311908 | 1 | 0.456551705 |
| TCGA-ED-A66Y | TCGA | C | B | 0.605093738 | 0.640723387 | 0.299258498 | 0.355764044 | 0.45605006 |
| TCGA-DD-AAW1 | TCGA | C | B | 0.771998612 | 0.899763039 | 0.410324589 | 0.762628644 | 0.252338787 |
| TCGA-DD-AACG | TCGA | A | B | 0.797417654 | 0.891290276 | 0.573078892 | 0.812633981 | 0.372364539 |
| TCGA-UB-AA0U | TCGA | C | A | 0.518121062 | 0.59372017 | 0.15478107 | 0.701137603 | 0.659646846 |
| TCGA-CC-A9FV | TCGA | C | C | 0.691551021 | 0.816254987 | 0.347894192 | 0.197137886 | 0.195991348 |
| TCGA-DD-A1EJ | TCGA | B | A | 0.750260366 | 0.783547521 | 0.482120794 | 0.885589511 | 0.642937014 |
| TCGA-XR-A8TF | TCGA | B | A | 0.758271333 | 0.841720108 | 0.473159597 | 0.554612079 | 0.442879404 |
| TCGA-DD-AADO | TCGA | B | B | 0.76465737 | 0.822996315 | 0.618223359 | 0.990646629 | 0.475188703 |
| TCGA-DD-AAVZ | TCGA | C | A | 0.621644655 | 0.689073415 | 0.240160091 | 0.778300593 | 0.321952324 |
| TCGA-K7-AAU7 | TCGA | B | C | 0.688657553 | 0.642993682 | 0.567001215 | 0.253315892 | 0.493339936 |
| TCGA-DD-A39Y | TCGA | B | A | 0.890779001 | 0.959718185 | 0.561947811 | 0.776112101 | 0.657755088 |
| TCGA-CC-A9FU | TCGA | B | A | 0.714178099 | 0.69599145 | 0.547084961 | 0.859996253 | 0.625534446 |
| TCGA-DD-A4NV | TCGA | C | A | 0.677360504 | 0.833503949 | 0.332800666 | 0.814484535 | 0.495111289 |
| TCGA-EP-A2KA | TCGA | B | A | 0.74519314 | 0.852521302 | 0.395942875 | 0.660881808 | 0.423020549 |
| TCGA-XR-A8TC | TCGA | B | A | 0.63428013 | 0.644441797 | 0.38137452 | 0.886387614 | 0.458158752 |
| TCGA-UB-A7MB | TCGA | C | A | 0.587822134 | 0.693749036 | 0.153339653 | 0.751302589 | 0.630101069 |
| TCGA-DD-A3A3 | TCGA | A | B | 0.770578321 | 0.91138845 | 0.40037714 | 0.861366745 | 0.186722454 |
| TCGA-BC-A69H | TCGA | B | A | 0.7307844 | 0.73951799 | 0.399422957 | 0.841119601 | 0.58460721 |
| TCGA-CC-A7IK | TCGA | B | A | 0.841235467 | 0.966939576 | 0.506999623 | 0.6439615 | 0.444182824 |
| TCGA-DD-A4NK | TCGA | A | A | 0.641400072 | 0.777283607 | 0.231595227 | 0.914288041 | 0.500121868 |
| TCGA-BC-A10R | TCGA | C | C | 0.801978399 | 0.852631343 | 0.606140929 | 0.317573967 | 0.103822579 |
| TCGA-DD-AADB | TCGA | B | A | 0.766238456 | 0.784435092 | 0.513439832 | 0.819851054 | 0.60145958 |
| TCGA-5R-AAAM | TCGA | A | A | 0.682551899 | 0.801884343 | 0.351765667 | 0.851051118 | 0.485106474 |
| TCGA-K7-A6G5 | TCGA | C | B | 0.770973803 | 0.873542958 | 0.45712895 | 0.745833244 | 0.273549519 |
| TCGA-HP-A5N0 | TCGA | A | A | 0.598873918 | 0.719901007 | 0.224211231 | 0.668245756 | 0.391055876 |
| TCGA-CC-A7IH | TCGA | C | A | 0.67786687 | 0.80419761 | 0.246617765 | 0.852931778 | 0.249866096 |
| TCGA-FV-A2QR | TCGA | C | A | 0.681421689 | 0.746013541 | 0.399266749 | 0.885711073 | 0.578376649 |
| TCGA-DD-AAEK | TCGA | A | B | 0.693141098 | 0.801331864 | 0.414301361 | 0.822357288 | 0.463366488 |
| TCGA-DD-A73D | TCGA | C | A | 0.7615412 | 0.884263927 | 0.341588546 | 0.821323657 | 0.389192123 |
| TCGA-DD-AAD6 | TCGA | A | B | 0.76461393 | 0.778197511 | 0.507277621 | 0.786802693 | 0.620396427 |
| TCGA-G3-AAV6 | TCGA | B | A | 0.620665851 | 0.56164163 | 0.403770069 | 0.821106929 | 0.544710506 |
| TCGA-LG-A6GG | TCGA | B | A | 0.63943589 | 0.773838707 | 0.285038868 | 0.661252324 | 0.306778253 |
| TCGA-ZS-A9CE | TCGA | B | A | 0.642143203 | 0.774839917 | 0.193551838 | 0.787550254 | 0.430733828 |
| TCGA-KR-A7K0 | TCGA | C | B | 0.765545474 | 0.829454166 | 0.561298994 | 0.766391396 | 0.328911306 |
| TCGA-DD-AADC | TCGA | C | A | 0.741813846 | 0.78623496 | 0.419323768 | 0.899846593 | 0.556603603 |
| TCGA-G3-A25Y | TCGA | B | A | 0.65173173 | 0.725952839 | 0.353098693 | 0.683736342 | 0.454189891 |
| TCGA-GJ-A3OU | TCGA | C | C | 0.678897286 | 0.743952454 | 0.374815224 | 0.661960591 | 0.646442463 |
| TCGA-ED-A82E | TCGA | B | C | 0.667425527 | 0.681969348 | 0.345611802 | 0.174134279 | 0.111732492 |
| TCGA-DD-AAD3 | TCGA | C | A | 0.589816152 | 0.715947757 | 0.219278685 | 0.575865325 | 0.522135825 |
| TCGA-ZP-A9CZ | TCGA | B | A | 0.618944093 | 0.67201601 | 0.275520742 | 0.840321492 | 0.561668163 |
| TCGA-DD-AAVS | TCGA | B | A | 0.625527883 | 0.613504565 | 0.320296284 | 0.624217758 | 0.260722057 |
| TCGA-KR-A7K7 | TCGA | B | A | 0.82302508 | 0.802082945 | 0.592523295 | 0.751151015 | 0.358321108 |
| TCGA-DD-AACB | TCGA | C | B | 0.848296954 | 0.906058411 | 0.520559738 | 0.792265142 | 0.424420499 |
| TCGA-T1-A6J8 | TCGA | B | A | 0.713738181 | 0.73463796 | 0.429899923 | 0.707426969 | 0.579274857 |
| TCGA-2Y-A9H1 | TCGA | A | B | 0.728816853 | 0.854685166 | 0.358360802 | 0.808029147 | 0.074818088 |
| TCGA-LG-A9QD | TCGA | A | B | 0.723973468 | 0.843463388 | 0.350186486 | 0.742571087 | 0.296525106 |
| TCGA-DD-AAD8 | TCGA | A | B | 0.591966902 | 0.656947703 | 0.41849692 | 0.495407556 | 0.500204019 |
| TCGA-DD-A1ED | TCGA | C | B | 0.671964961 | 0.799080235 | 0.336456333 | 0.468587743 | 0.269631212 |
| TCGA-ZP-A9CY | TCGA | A | B | 0.606284398 | 0.74941887 | 0.24588152 | 0.661934386 | 0.448053301 |
| TCGA-DD-AACF | TCGA | A | B | 0.633795086 | 0.758623813 | 0.286430731 | 0.545574821 | 0.412632578 |
| TCGA-ED-A627 | TCGA | C | C | 0.6126565 | 0.704963208 | 0.330395915 | 0.400848646 | 0.433817668 |
| TCGA-DD-A73A | TCGA | A | B | 0.672223154 | 0.785251207 | 0.273593106 | 0.75527544 | 0.384897601 |
| TCGA-CC-A8HS | TCGA | B | A | 0.621678259 | 0.58961541 | 0.363110845 | 0.567981489 | 0.541776896 |
| TCGA-WX-AA46 | TCGA | C | B | 0.713375338 | 0.857600077 | 0.363717511 | 0.57118592 | 0.356851574 |
| TCGA-DD-AADW | TCGA | B | A | 0.539440387 | 0.602887281 | 0.167429197 | 0.854937002 | 0.602677393 |
| TCGA-DD-A1EE | TCGA | B | A | 0.666196037 | 0.740482229 | 0.234704663 | 0.786537908 | 0.556334019 |
| TCGA-BC-A112 | TCGA | B | C | 0.772219304 | 0.773843277 | 0.565698937 | 0.469508951 | 0.48053123 |
| TCGA-2Y-A9GV | TCGA | A | A | 0.604281012 | 0.673470487 | 0.364239141 | 0.719254685 | 0.513838792 |
| TCGA-DD-AACO | TCGA | A | B | 0.555375836 | 0.600486084 | 0.363075312 | 0.724047608 | 0.193427013 |
| TCGA-5C-A9VH | TCGA | B | A | 0.637982086 | 0.701369725 | 0.295865258 | 0.71010164 | 0.194712929 |
| TCGA-G3-A7M8 | TCGA | C | B | 0.754475752 | 0.792358248 | 0.466877039 | 0.54395758 | 0.437790053 |
| TCGA-UB-A7MA | TCGA | B | C | 0.723503085 | 0.714815497 | 0.559491366 | 0.104343207 | 0.381254693 |
| TCGA-DD-AAD0 | TCGA | C | A | 0.71579248 | 0.73573298 | 0.501272855 | 0.716388942 | 0.791800248 |
| TCGA-KR-A7K2 | TCGA | A | B | 0.726332702 | 0.841700838 | 0.482268212 | 0.801917491 | 0.122963263 |
| TCGA-K7-A5RG | TCGA | C | A | 0.745937394 | 0.806198143 | 0.603323615 | 0.474742787 | 0.62576239 |
| TCGA-CC-A123 | TCGA | A | B | 0.708822088 | 0.732679994 | 0.528539564 | 0.790651993 | 0.256002168 |
| TCGA-DD-A1EG | TCGA | B | A | 0.772598207 | 0.811407529 | 0.517187536 | 0.812125694 | 0.458524353 |
| TCGA-CC-A8HU | TCGA | B | A | 0.792788298 | 0.782034863 | 0.745273407 | 0.707076346 | 0.778754303 |
| TCGA-BC-A69I | TCGA | A | B | 0.655294932 | 0.758002426 | 0.34801412 | 0.752859937 | 0.215710413 |
| TCGA-DD-AAE2 | TCGA | C | A | 0.608185057 | 0.77463705 | 0.097444823 | 0.577965691 | 0.580312541 |
| TCGA-DD-A1EH | TCGA | B | A | 0.568767947 | 0.637508 | 0.203578622 | 0.697379777 | 0.570343427 |
| TCGA-4R-AA8I | TCGA | C | B | 0.593868008 | 0.68120544 | 0.17155169 | 0.268215036 | 0.397243469 |
| TCGA-DD-AACW | TCGA | B | A | 0.668217567 | 0.684152272 | 0.302256368 | 0.887491381 | 0.498691424 |
| TCGA-RC-A7SF | TCGA | A | B | 0.62086774 | 0.710448491 | 0.216342566 | 0.695200304 | 0.255757363 |
| TCGA-ED-A4XI | TCGA | C | B | 0.632940473 | 0.698470363 | 0.352093199 | 0.607888885 | 0.355783099 |
| TCGA-DD-A1EK | TCGA | A | A | 0.529606465 | 0.637671241 | 0.108204357 | 0.790885139 | 0.736044802 |
| TCGA-G3-AAV5 | TCGA | B | A | 0.837445863 | 0.962400357 | 0.433841842 | 0.744392556 | 0.330375962 |
| TCGA-5R-AA1D | TCGA | A | C | 0.641152311 | 0.773856471 | 0.29173549 | 0.405541816 | 0.31372835 |
| TCGA-EP-A2KC | TCGA | B | A | 0.732384194 | 0.865813137 | 0.356562516 | 0.75520908 | 0.344452144 |
| TCGA-DD-AAW3 | TCGA | A | B | 0.67913334 | 0.789236146 | 0.219227419 | 0.834844436 | 0.354695505 |
| TCGA-DD-AADS | TCGA | A | B | 0.666282691 | 0.766139082 | 0.484299609 | 0.774325479 | 0.181903722 |
| TCGA-DD-AA3A | TCGA | B | C | 0.734589056 | 0.675980841 | 0.712209642 | 0 | 0.490235282 |
| TCGA-DD-AACH | TCGA | B | C | 0.680663662 | 0.661660019 | 0.388516006 | 0.5608094 | 0.480042318 |
| TCGA-DD-A73C | TCGA | C | A | 0.692468215 | 0.853504735 | 0.332682838 | 0.845299923 | 0.517579721 |
| TCGA-ED-A8O5 | TCGA | C | A | 0.634442322 | 0.636648056 | 0.368450916 | 0.617941075 | 0.422105115 |
| TCGA-NI-A8LF | TCGA | C | A | 0.777184017 | 0.857457285 | 0.557831445 | 0.805543319 | 0.301963873 |
| TCGA-DD-A11A | TCGA | C | A | 0.764581181 | 0.882605727 | 0.44776925 | 0.976894382 | 0.467443366 |
| TCGA-DD-AAVP | TCGA | A | B | 0.868387036 | 0.915546436 | 0.620835381 | 0.787643994 | 0.541596182 |
| TCGA-DD-A4NI | TCGA | C | A | 0.619520098 | 0.780106802 | 0.274132046 | 0.578589722 | 0.389588852 |
| TCGA-DD-AAC9 | TCGA | A | B | 0.737333316 | 0.859890043 | 0.412875486 | 0.910156386 | 0.378673008 |
| TCGA-DD-A3A6 | TCGA | A | C | 0.67261549 | 0.767671056 | 0.459708238 | 0.223523476 | 0 |
| TCGA-DD-A39W | TCGA | C | A | 0.777348611 | 0.795092639 | 0.390643864 | 0.95083407 | 0.413346651 |
| TCGA-BC-A10T | TCGA | B | A | 0.635190796 | 0.762335427 | 0.214273432 | 0.899570031 | 0.45199402 |
| TCGA-DD-A4NO | TCGA | C | A | 0.705175142 | 0.842326025 | 0.398665385 | 0.912808173 | 0.249879186 |
| TCGA-EP-A2KB | TCGA | B | A | 0.753958308 | 0.773478224 | 0.550986885 | 0.78385737 | 0.402105044 |
| TCGA-UB-A7MC | TCGA | B | A | 0.812195548 | 0.886800795 | 0.568716686 | 0.758911646 | 0.465729681 |
| TCGA-2Y-A9H7 | TCGA | B | A | 0.676033331 | 0.700370385 | 0.284710909 | 0.911554119 | 0.438148936 |
| TCGA-DD-AADY | TCGA | A | B | 0.635153457 | 0.77224041 | 0.157661803 | 0.605728981 | 0.407929894 |
| TCGA-DD-A3A7 | TCGA | A | B | 0.751056451 | 0.783184407 | 0.589424615 | 0.590672453 | 0.370306478 |
| TCGA-DD-AACQ | TCGA | C | A | 0.663741344 | 0.732035577 | 0.316530312 | 0.671654968 | 0.450897148 |
| TCGA-DD-A4NN | TCGA | B | A | 0.649489882 | 0.636739325 | 0.423181865 | 0.793636265 | 0.429839252 |
| TCGA-DD-AACK | TCGA | B | A | 0.774224516 | 0.847894202 | 0.461470941 | 0.919805462 | 0.573507287 |
| TCGA-DD-AADQ | TCGA | B | B | 0.717497676 | 0.765066519 | 0.434284777 | 0.581936936 | 0.245764828 |
| TCGA-BC-A10S | TCGA | B | B | 0.714728188 | 0.838095801 | 0.350266574 | 0.533720762 | 0.328113473 |
| TCGA-GJ-A9DB | TCGA | C | A | 0.563965231 | 0.692042269 | 0.174820348 | 0.776625641 | 0.519465482 |
| TCGA-BD-A2L6 | TCGA | C | A | 0.630113775 | 0.770135836 | 0.317964206 | 0.669029189 | 0.410557821 |
| TCGA-NI-A4U2 | TCGA | B | A | 0.853081934 | 1 | 0.607459463 | 0.558674989 | 0.305239006 |
| TCGA-ZP-A9D2 | TCGA | B | C | 0.662115916 | 0.726471356 | 0.367721897 | 0.312281033 | 0.437591302 |
| TCGA-DD-AAW2 | TCGA | A | B | 0.63567748 | 0.733561288 | 0.153205363 | 0.722589191 | 0.304485325 |
| TCGA-XR-A8TE | TCGA | C | A | 0.809797229 | 0.847672096 | 0.627126729 | 0.82322468 | 0.390228517 |
| TCGA-DD-A11B | TCGA | C | A | 0.66311209 | 0.746471824 | 0.392675046 | 0.645452147 | 0.408380639 |
| TCGA-O8-A75V | TCGA | B | A | 0.720318209 | 0.813985733 | 0.357345923 | 0.833975419 | 0.364704315 |
| TCGA-BW-A5NP | TCGA | B | A | 0.65108746 | 0.682412674 | 0.347696698 | 0.83015183 | 0.619084675 |
| TCGA-2Y-A9GU | TCGA | A | B | 0.736835489 | 0.864403144 | 0.387055643 | 0.581247132 | 0.466623691 |
| TCGA-G3-AAV2 | TCGA | C | B | 0.756472376 | 0.856214867 | 0.465869753 | 0.679615053 | 0.215876859 |
| TCGA-DD-AAVU | TCGA | A | B | 0.754600773 | 0.910057298 | 0.334185397 | 0.686101088 | 0.217666363 |
| TCGA-DD-AACP | TCGA | B | A | 0.781954032 | 0.75247364 | 0.558337912 | 0.814112504 | 0.83929381 |
| TCGA-CC-A3MB | TCGA | B | A | 0.776773515 | 0.792096198 | 0.543443335 | 0.759856479 | 0.630213581 |
| TCGA-RC-A7SH | TCGA | C | A | 0.644809085 | 0.642900672 | 0.474078563 | 0.802681167 | 0.308124658 |
| TCGA-DD-AAE4 | TCGA | A | B | 0.644296116 | 0.779622292 | 0.362736674 | 0.701878738 | 0.324200059 |
| TCGA-G3-A25X | TCGA | B | C | 0.753624336 | 0.813909823 | 0.46717412 | 0.51224622 | 0.384400926 |
| TCGA-G3-A7M5 | TCGA | A | A | 0.70641711 | 0.783998683 | 0.434970266 | 0.911630715 | 0.404605512 |
| TCGA-G3-A5SI | TCGA | A | B | 0.5860942 | 0.691195932 | 0.255054991 | 0.550422466 | 0.423022591 |
| TCGA-RC-A6M4 | TCGA | B | A | 0.666312174 | 0.788496282 | 0.204579565 | 0.963419745 | 0.454260181 |
| TCGA-WQ-A9G7 | TCGA | B | B | 0.741728251 | 0.728789536 | 0.609255222 | 0.701870099 | 0.447165401 |
| TCGA-CC-A1HT | TCGA | B | C | 0.78412444 | 0.764610338 | 0.657213595 | 0.289801619 | 0.563102246 |
| TCGA-G3-A25V | TCGA | C | B | 0.768314467 | 0.861013487 | 0.463752801 | 0.543883228 | 0.412130598 |
| TCGA-G3-A25S | TCGA | B | B | 0.813737493 | 0.843416569 | 0.604041315 | 0.623980718 | 0.308103101 |
| TCGA-DD-AACJ | TCGA | B | A | 0.845250077 | 0.936989945 | 0.594762089 | 0.921493923 | 0.353862256 |
| TCGA-DD-A3A9 | TCGA | B | B | 0.819694003 | 0.885159667 | 0.70526554 | 0.736663363 | 0.345226347 |
| TCGA-KR-A7K8 | TCGA | A | A | 0.677885889 | 0.81514771 | 0.308754686 | 0.733440244 | 0.634043802 |
| TCGA-2Y-A9GS | TCGA | B | A | 0.687538828 | 0.760468787 | 0.353291746 | 0.62382877 | 0.641232902 |
| TCGA-CC-5264 | TCGA | B | A | 0.576250105 | 0.65805237 | 0.138926972 | 0.732779937 | 0.671396496 |
| TCGA-ED-A8O6 | TCGA | B | A | 0.767657045 | 0.768862498 | 0.591967761 | 0.821455747 | 0.447957225 |
| TCGA-DD-A4NE | TCGA | C | A | 0.506563359 | 0.613929433 | 0.083148294 | 0.772144023 | 0.614988038 |
| TCGA-ZS-A9CD | TCGA | C | B | 0.770541529 | 0.867385043 | 0.459945538 | 0.820670707 | 0.317418074 |
| TCGA-DD-AACI | TCGA | A | B | 0.769390331 | 0.801252378 | 0.562835717 | 0.55044547 | 0.299420455 |
| TCGA-ED-A97K | TCGA | C | C | 0.491281341 | 0.54368359 | 0.271327361 | 0.22952112 | 0.514390106 |
| TCGA-DD-A114 | TCGA | C | C | 0.597661465 | 0.642001705 | 0.229083454 | 0.507709317 | 0.637851427 |
| TCGA-MI-A75E | TCGA | C | A | 0.710797598 | 0.829684201 | 0.355797799 | 0.806316213 | 0.408473996 |
| TCGA-G3-AAV3 | TCGA | C | B | 0.705345424 | 0.741162417 | 0.435625088 | 0.6256564 | 0.312491697 |
| TCGA-DD-A73B | TCGA | B | A | 0.758105372 | 0.833279706 | 0.51629625 | 0.830182864 | 0.768833685 |
| TCGA-DD-AAEE | TCGA | B | A | 0.82083082 | 0.88010567 | 0.609971844 | 0.740939479 | 0.271192291 |
| TCGA-DD-AACV | TCGA | A | A | 0.632211421 | 0.796081611 | 0.299215025 | 0.880623224 | 0.665561833 |
| TCGA-DD-AACA | TCGA | B | A | 0.713938683 | 0.8580816 | 0.387877527 | 0.769173601 | 0.352350107 |
| TCGA-G3-A6UC | TCGA | A | A | 0.59150785 | 0.723586957 | 0.204056917 | 0.885706206 | 0.342677391 |
| TCGA-FV-A3I0 | TCGA | B | C | 0.811901316 | 0.757675313 | 0.654629318 | 0.04252049 | 0.241683179 |
| TCGA-ED-A7PZ | TCGA | B | A | 0.87686824 | 0.933828216 | 0.701084266 | 0.803664729 | 0.602024699 |
| TCGA-CC-A3MC | TCGA | B | A | 0.670662399 | 0.755298236 | 0.305241044 | 0.644009773 | 0.597715511 |
| TCGA-DD-AAEB | TCGA | A | B | 0.686215461 | 0.79698066 | 0.471240381 | 0.872731391 | 0.160006933 |
| TCGA-2V-A95S | TCGA | B | C | 0.76237818 | 0.806769038 | 0.529384318 | 0.651796758 | 0.279295845 |
| TCGA-G3-AAV0 | TCGA | A | B | 0.694872341 | 0.841445127 | 0.390865385 | 0.588321015 | 0.359472132 |
| TCGA-CC-A3MA | TCGA | B | C | 0.691594655 | 0.740511748 | 0.432177454 | 0.340062443 | 0.437946983 |
| TCGA-HP-A5MZ | TCGA | C | A | 0.705110886 | 0.782867316 | 0.413632172 | 0.75215065 | 0.337394218 |
| TCGA-WX-AA44 | TCGA | C | A | 0.679611717 | 0.688966867 | 0.508184295 | 0.769943035 | 0.455684928 |
| TCGA-DD-A4NQ | TCGA | C | C | 0.754204687 | 0.864983699 | 0.331067114 | 0.772438529 | 0.831570303 |
| TCGA-DD-A113 | TCGA | B | A | 0.570057569 | 0.621514019 | 0.20279474 | 0.705614523 | 0.47952055 |
| TCGA-MI-A75G | TCGA | A | A | 0.705637173 | 0.88005495 | 0.325366691 | 0.830725753 | 0.29841843 |
| TCGA-DD-AACS | TCGA | A | A | 0.601170861 | 0.749796665 | 0.196824435 | 0.691965734 | 0.478997585 |
| TCGA-DD-AAE1 | TCGA | A | B | 0.698283505 | 0.727954072 | 0.387624598 | 0.913680505 | 0.425044502 |
| TCGA-5C-A9VG | TCGA | B | A | 0.822742832 | 0.869909379 | 0.561924056 | 0.764688084 | 0.669191186 |
| TCGA-2Y-A9H2 | TCGA | C | C | 0.635956616 | 0.650776124 | 0.341288288 | 0.23981603 | 0.453644597 |
| TCGA-2Y-A9H0 | TCGA | B | A | 0.639648363 | 0.63614604 | 0.404159211 | 0.641959794 | 0.632756261 |
| TCGA-G3-A7M6 | TCGA | C | C | 0.606965259 | 0.608011224 | 0.369047182 | 0.299782301 | 0.318612925 |
| TCGA-RG-A7D4 | TCGA | B | A | 0.813487553 | 0.821093704 | 0.543397465 | 0.775351285 | 0.583559803 |
| TCGA-CC-5261 | TCGA | C | C | 0.529051747 | 0.539242163 | 0.289011899 | 0.615313283 | 0.41129406 |
| TCGA-CC-A7IF | TCGA | A | A | 0.589912601 | 0.718436344 | 0.191902276 | 0.745407264 | 0.250956616 |
| TCGA-QA-A7B7 | TCGA | B | A | 0.705574093 | 0.757039084 | 0.46983461 | 0.836142166 | 0.812546144 |
| TCGA-DD-AACD | TCGA | A | B | 0.775730755 | 0.865501394 | 0.467684739 | 0.765793501 | 0.24329225 |
| TCGA-BC-A217 | TCGA | B | A | 0.616047391 | 0.755129826 | 0.293145374 | 0.581484529 | 0.406831112 |
| TCGA-DD-AAD2 | TCGA | A | B | 0.712377305 | 0.843456559 | 0.307502853 | 0.748449908 | 0.443766273 |
| TCGA-DD-AACN | TCGA | C | A | 0.692955588 | 0.783630131 | 0.391221201 | 0.587269673 | 0.367431469 |
| TCGA-DD-A3A1 | TCGA | B | B | 0.794965838 | 0.838421528 | 0.589165296 | 0.879605932 | 0.436698124 |
| TCGA-DD-A3A2 | TCGA | A | A | 0.771809215 | 0.929819447 | 0.433125044 | 0.836622651 | 0.565811751 |
| TCGA-ED-A66X | TCGA | B | A | 0.635399845 | 0.671336787 | 0.358893134 | 0.835775939 | 0.514174414 |
| TCGA-WQ-AB4B | TCGA | A | B | 0.772097074 | 0.858364603 | 0.586212108 | 0.818566371 | 0.297044268 |
| TCGA-DD-AACY | TCGA | C | B | 0.731339727 | 0.83095522 | 0.367266921 | 0.775243195 | 0.39397863 |
| TCGA-BC-A10X | TCGA | C | A | 0.677275953 | 0.817466121 | 0.340716013 | 0.579687534 | 0.526272701 |
| TCGA-DD-AACU | TCGA | C | A | 0.687943594 | 0.728892029 | 0.315909337 | 0.765220846 | 0.414037052 |
| TCGA-RC-A6M3 | TCGA | B | C | 0.726990465 | 0.776668504 | 0.452919975 | 0.255250066 | 0.754590762 |
| TCGA-5C-AAPD | TCGA | C | B | 0.797974051 | 0.827912693 | 0.589107781 | 0.443636062 | 0.478345738 |
| TCGA-CC-A7II | TCGA | B | C | 0.828297828 | 0.846235669 | 0.677665001 | 0.462572287 | 0.616728778 |
| TCGA-BC-A3KF | TCGA | B | A | 0.700678751 | 0.780234663 | 0.409673078 | 0.831987721 | 0.479443601 |
| TCGA-DD-A4NF | TCGA | C | A | 0.750901567 | 0.875358735 | 0.406330534 | 0.824133993 | 0.244751231 |
| TCGA-DD-A11D | TCGA | A | B | 0.647094838 | 0.810152394 | 0.288708459 | 0.812949257 | 0.595602037 |
| TCGA-DD-A116 | TCGA | B | B | 0.737478485 | 0.828187424 | 0.463596631 | 0.730143193 | 0.33673209 |
| TCGA-DD-A11C | TCGA | C | A | 0.54662408 | 0.649743886 | 0.213550258 | 0.477640902 | 0.409536571 |
| TCGA-DD-AACC | TCGA | A | B | 0.681182603 | 0.745819541 | 0.494904544 | 0.891126238 | 0.563564256 |
| TCGA-ES-A2HS | TCGA | A | B | 0.633021117 | 0.768275984 | 0.271899613 | 0.60647467 | 0.067058452 |
| TCGA-2Y-A9H3 | TCGA | A | B | 0.759674307 | 0.803318526 | 0.612720105 | 0.417499308 | 0.277434523 |
| TCGA-DD-AAE0 | TCGA | A | B | 0.738995448 | 0.738085198 | 0.599086956 | 0.661161628 | 0.452302728 |
| TCGA-G3-A25U | TCGA | C | A | 0.700322064 | 0.830561931 | 0.275406311 | 0.760817236 | 0.506606933 |
| TCGA-G3-AAV4 | TCGA | C | B | 0.76416206 | 0.846712103 | 0.503232354 | 0.820850234 | 0.473449413 |
| TCGA-RC-A6M6 | TCGA | B | C | 0.747788529 | 0.765805171 | 0.541081862 | 0.48692596 | 0.642038641 |
| TCGA-G3-A7M7 | TCGA | B | B | 0.840152319 | 0.897687479 | 0.530766906 | 0.600359068 | 0.318602762 |
| TCGA-BC-A10Y | TCGA | B | A | 0.54362703 | 0.639790267 | 0.209294689 | 0.726119305 | 0.460714812 |
| TCGA-2Y-A9H5 | TCGA | C | A | 0.585461405 | 0.640208452 | 0.286738675 | 0.765450522 | 0.329551587 |
| TCGA-DD-A73E | TCGA | A | A | 0.60192691 | 0.731933978 | 0.132305791 | 0.760140726 | 0.396592031 |
| TCGA-ZP-A9D1 | TCGA | C | A | 0.653337223 | 0.786833403 | 0.324708147 | 0.631060611 | 0.462979387 |
| TCGA-G3-A7M9 | TCGA | B | C | 0.757522651 | 0.702737355 | 0.552141218 | 0.466240887 | 1 |
| TCGA-DD-AADU | TCGA | A | B | 0.80997507 | 0.91009423 | 0.541212836 | 0.759840897 | 0.233533084 |
| TCGA-WJ-A86L | TCGA | C | A | 0.580597444 | 0.63159596 | 0.343919862 | 0.873810027 | 0.280268272 |
| TCGA-CC-A9FW | TCGA | B | A | 0.685535015 | 0.643221956 | 0.472994908 | 0.947826417 | 0.547264595 |
| TCGA-EP-A3RK | TCGA | B | A | 0.742237203 | 0.781563343 | 0.443757539 | 0.80917023 | 0.486033722 |
| TCGA-3K-AAZ8 | TCGA | A | A | 0.716446445 | 0.890186405 | 0.317153922 | 0.954335289 | 0.341795957 |
| TCGA-DD-A3A4 | TCGA | A | B | 0.728541276 | 0.882350947 | 0.340946482 | 0.511235993 | 0.353035153 |
| TCGA-MR-A8JO | TCGA | C | C | 0.734086682 | 0.792673418 | 0.482655417 | 0.159516958 | 0.340654851 |
| TCGA-ED-A7PY | TCGA | A | B | 0.646507826 | 0.703576866 | 0.433644561 | 0.733434234 | 0.162745428 |
| TCGA-DD-AAVW | TCGA | C | A | 0.544764141 | 0.599576378 | 0.240671806 | 0.701144547 | 0.309171727 |
| TCGA-DD-AADR | TCGA | A | B | 0.610848593 | 0.690814484 | 0.148834822 | 0.71946775 | 0.355247362 |
| TCGA-DD-AAEH | TCGA | A | B | 0.703587879 | 0.789420288 | 0.39565279 | 0.685066572 | 0.312400079 |
| TCGA-2Y-A9HB | TCGA | C | A | 0.687131952 | 0.84703767 | 0.304398849 | 0.789973306 | 0.458300144 |
| TCGA-DD-AADG | TCGA | C | A | 0.712205194 | 0.782284492 | 0.309759914 | 0.82110019 | 0.468104406 |
| TCGA-DD-A3A8 | TCGA | C | B | 0.717725191 | 0.795322778 | 0.247159629 | 0.816183448 | 0.300676599 |
| TCGA-G3-AAV7 | TCGA | B | C | 0.766538216 | 0.796101777 | 0.422377568 | 0.500656502 | 0.742563623 |
| TCGA-CC-5263 | TCGA | B | C | 0.521441775 | 0.531679062 | 0.173215588 | 0.561683977 | 0.851500681 |
| TCGA-2Y-A9H8 | TCGA | C | A | 0.56163903 | 0.556452468 | 0.166819846 | 0.895099434 | 0.592121554 |
| TCGA-LG-A9QC | TCGA | C | A | 0.828798413 | 0.887999072 | 0.568104832 | 0.843753967 | 0.508914775 |
| TCGA-CC-5258 | TCGA | B | A | 0.697855727 | 0.694602979 | 0.387740796 | 0.855420651 | 0.821746315 |
| TCGA-DD-AAD5 | TCGA | B | C | 0.821289491 | 0.822386683 | 0.650296839 | 0.401919948 | 0.533073296 |
| TCGA-BD-A3EP | TCGA | C | A | 0.709962789 | 0.757927618 | 0.453714191 | 0.669694966 | 0.496885388 |
| TCGA-DD-AAW0 | TCGA | C | A | 0.605651301 | 0.741633249 | 0.271511563 | 0.755929016 | 0.566924022 |
| TCGA-EP-A12J | TCGA | C | A | 0.626458486 | 0.704181802 | 0.24406658 | 0.805155368 | 0.251742801 |
| TCGA-DD-AAE7 | TCGA | A | B | 0.703731577 | 0.830586344 | 0.323946086 | 0.702629673 | 0.431877565 |
| TCGA-BC-4073 | TCGA | B | C | 0.619477801 | 0.630712187 | 0.419548066 | 0.545608637 | 0.730210741 |
| TCGA-DD-A4NL | TCGA | A | B | 0.595510426 | 0.714913906 | 0.280278134 | 0.856434421 | 0.373548504 |
| TCGA-BC-A3KG | TCGA | B | A | 0.652694229 | 0.66894799 | 0.451683149 | 0.832452386 | 0.75615163 |
| TCGA-DD-A1EB | TCGA | C | A | 0.760122166 | 0.888927141 | 0.439134693 | 0.728148537 | 0.60492283 |
| DO23508 | ICGC | B | NA | 0.738699652 | 0.871499621 | 0.315371746 | NA | NA |
| DO23509 | ICGC | C | NA | 0.728477051 | 0.835835323 | 0.430276887 | NA | NA |
| DO23510 | ICGC | C | NA | 0.707838859 | 0.863833608 | 0.306814307 | NA | NA |
| DO23511 | ICGC | B | NA | 0.771339758 | 0.845251363 | 0.485625147 | NA | NA |
| DO23512 | ICGC | C | NA | 0.634134669 | 0.793231939 | 0.236718596 | NA | NA |
| DO23513 | ICGC | C | NA | 0.710725383 | 0.896109086 | 0.278483742 | NA | NA |
| DO23514 | ICGC | C | NA | 0.684093651 | 0.859446895 | 0.256205439 | NA | NA |
| DO23515 | ICGC | B | NA | 0.692862563 | 0.780897212 | 0.312735026 | NA | NA |
| DO23516 | ICGC | C | NA | 0.646223368 | 0.818563063 | 0.158353943 | NA | NA |
| DO23517 | ICGC | A | NA | 0.68705224 | 0.872382137 | 0.283629466 | NA | NA |
| DO23518 | ICGC | C | NA | 0.70478857 | 0.874354783 | 0.267414843 | NA | NA |
| DO23519 | ICGC | B | NA | 0.755654495 | 0.842308917 | 0.404183665 | NA | NA |
| DO23521 | ICGC | B | NA | 0.675929164 | 0.775423844 | 0.230618149 | NA | NA |
| DO23523 | ICGC | B | NA | 0.76857822 | 0.89816218 | 0.439640982 | NA | NA |
| DO23524 | ICGC | C | NA | 0.64423973 | 0.808125563 | 0.216394642 | NA | NA |
| DO23525 | ICGC | C | NA | 0.63947973 | 0.805582698 | 0.125935063 | NA | NA |
| DO23526 | ICGC | B | NA | 0.71919274 | 0.833761996 | 0.350345775 | NA | NA |
| DO23527 | ICGC | A | NA | 0.635390745 | 0.837843907 | 0.16219237 | NA | NA |
| DO23528 | ICGC | C | NA | 0.634125104 | 0.774260809 | 0.120330405 | NA | NA |
| DO23529 | ICGC | A | NA | 0.637275801 | 0.806846832 | 0.19100378 | NA | NA |
| DO23530 | ICGC | C | NA | 0.667522965 | 0.849540345 | 0.261033848 | NA | NA |
| DO23531 | ICGC | A | NA | 0.610790709 | 0.815913152 | 0.148711127 | NA | NA |
| DO23532 | ICGC | A | NA | 0.572581209 | 0.732195484 | 0.127430896 | NA | NA |
| DO23533 | ICGC | C | NA | 0.704086193 | 0.887644524 | 0.298893425 | NA | NA |
| DO23534 | ICGC | A | NA | 0.639936124 | 0.82207316 | 0.220517035 | NA | NA |
| DO23535 | ICGC | C | NA | 0.674113989 | 0.817336231 | 0.325898135 | NA | NA |
| DO23536 | ICGC | C | NA | 0.589008002 | 0.804005119 | 0.105584359 | NA | NA |
| DO23537 | ICGC | C | NA | 0.633595927 | 0.720723114 | 0.241507166 | NA | NA |
| DO23538 | ICGC | A | NA | 0.613293245 | 0.867472321 | 0.106046632 | NA | NA |
| DO23539 | ICGC | C | NA | 0.654398733 | 0.819146868 | 0.234954519 | NA | NA |
| DO23540 | ICGC | C | NA | 0.672040188 | 0.837620871 | 0.180630196 | NA | NA |
| DO23541 | ICGC | C | NA | 0.696428986 | 0.883583574 | 0.295992905 | NA | NA |
| DO23542 | ICGC | C | NA | 0.696024385 | 0.872689486 | 0.284489049 | NA | NA |
| DO23543 | ICGC | C | NA | 0.603933571 | 0.76880492 | 0.139184814 | NA | NA |
| DO23544 | ICGC | A | NA | 0.609659821 | 0.830362844 | 0.162051536 | NA | NA |
| DO23545 | ICGC | C | NA | 0.740848799 | 0.904673818 | 0.290072724 | NA | NA |
| DO23546 | ICGC | C | NA | 0.597247812 | 0.778467526 | 0.129911082 | NA | NA |
| DO23547 | ICGC | B | NA | 0.670052439 | 0.803966298 | 0.366591068 | NA | NA |
| DO23548 | ICGC | C | NA | 0.596175925 | 0.771235971 | 0.14033996 | NA | NA |
| DO23549 | ICGC | C | NA | 0.645621227 | 0.802826902 | 0.29136313 | NA | NA |
| DO23550 | ICGC | A | NA | 0.676505815 | 0.853872333 | 0.223426699 | NA | NA |
| DO23551 | ICGC | A | NA | 0.641824707 | 0.840360619 | 0.228428767 | NA | NA |
| DO23552 | ICGC | A | NA | 0.709940325 | 0.7938435 | 0.360114614 | NA | NA |
| DO45091 | ICGC | C | NA | 0.693890438 | 0.891740334 | 0.184336408 | NA | NA |
| DO45092 | ICGC | C | NA | 0.704945669 | 0.874488148 | 0.264679978 | NA | NA |
| DO45093 | ICGC | B | NA | 0.783593734 | 0.884932435 | 0.446207446 | NA | NA |
| DO45094 | ICGC | A | NA | 0.617223792 | 0.811815308 | 0.153190635 | NA | NA |
| DO45095 | ICGC | C | NA | 0.655038015 | 0.84198347 | 0.219477334 | NA | NA |
| DO45096 | ICGC | A | NA | 0.658209544 | 0.865456991 | 0.191939208 | NA | NA |
| DO45097 | ICGC | C | NA | 0.641281943 | 0.814817826 | 0.1573728 | NA | NA |
| DO45099 | ICGC | A | NA | 0.682263843 | 0.878834294 | 0.271474287 | NA | NA |
| DO45101 | ICGC | A | NA | 0.612175349 | 0.822599689 | 0.166055393 | NA | NA |
| DO45103 | ICGC | C | NA | 0.638595574 | 0.825875511 | 0.221713634 | NA | NA |
| DO45105 | ICGC | A | NA | 0.628115118 | 0.748946992 | 0.289594108 | NA | NA |
| DO45107 | ICGC | A | NA | 0.612564038 | 0.789389771 | 0.201872535 | NA | NA |
| DO45109 | ICGC | A | NA | 0.656659206 | 0.836792261 | 0.276792727 | NA | NA |
| DO45111 | ICGC | A | NA | 0.639316635 | 0.865353052 | 0.186172405 | NA | NA |
| DO45113 | ICGC | A | NA | 0.64906994 | 0.802515708 | 0.148756771 | NA | NA |
| DO45115 | ICGC | C | NA | 0.69622183 | 0.847859944 | 0.333621934 | NA | NA |
| DO45117 | ICGC | C | NA | 0.630214949 | 0.820720529 | 0.067525708 | NA | NA |
| DO45119 | ICGC | B | NA | 0.70890247 | 0.857609497 | 0.273602618 | NA | NA |
| DO45121 | ICGC | C | NA | 0.584020303 | 0.701535188 | 0.064276501 | NA | NA |
| DO45123 | ICGC | C | NA | 0.656913995 | 0.805389461 | 0.169588632 | NA | NA |
| DO45125 | ICGC | A | NA | 0.645564076 | 0.829061314 | 0.21980944 | NA | NA |
| DO45127 | ICGC | B | NA | 0.765182009 | 0.804938741 | 0.440075961 | NA | NA |
| DO45129 | ICGC | C | NA | 0.667154679 | 0.8185457 | 0.336914809 | NA | NA |
| DO45131 | ICGC | B | NA | 0.739777225 | 0.89829849 | 0.34079748 | NA | NA |
| DO45133 | ICGC | C | NA | 0.639344486 | 0.859609477 | 0.162677751 | NA | NA |
| DO45135 | ICGC | C | NA | 0.716689318 | 0.872160477 | 0.341839883 | NA | NA |
| DO45137 | ICGC | C | NA | 0.615269224 | 0.799178058 | 0.159562383 | NA | NA |
| DO45139 | ICGC | C | NA | 0.693231129 | 0.850609092 | 0.266799647 | NA | NA |
| DO45141 | ICGC | C | NA | 0.633128577 | 0.824292544 | 0.191311126 | NA | NA |
| DO45143 | ICGC | C | NA | 0.692471111 | 0.874421575 | 0.276210285 | NA | NA |
| DO45145 | ICGC | B | NA | 0.713877308 | 0.790944822 | 0.39377973 | NA | NA |
| DO45147 | ICGC | B | NA | 0.739566046 | 0.822673323 | 0.447805455 | NA | NA |
| DO45149 | ICGC | C | NA | 0.684528981 | 0.817503387 | 0.343716089 | NA | NA |
| DO45153 | ICGC | C | NA | 0.664020957 | 0.837757763 | 0.226963705 | NA | NA |
| DO45155 | ICGC | A | NA | 0.642254987 | 0.825904341 | 0.198939841 | NA | NA |
| DO45157 | ICGC | C | NA | 0.696465643 | 0.861068214 | 0.27925247 | NA | NA |
| DO45159 | ICGC | A | NA | 0.607740799 | 0.779892686 | 0.14403816 | NA | NA |
| DO45161 | ICGC | C | NA | 0.635438692 | 0.799106727 | 0.08553767 | NA | NA |
| DO45163 | ICGC | C | NA | 0.72197369 | 0.912263131 | 0.313347044 | NA | NA |
| DO45165 | ICGC | C | NA | 0.673121989 | 0.848496404 | 0.205847798 | NA | NA |
| DO45167 | ICGC | A | NA | 0.563195496 | 0.742793616 | 0.129910139 | NA | NA |
| DO45169 | ICGC | C | NA | 0.691329905 | 0.859888049 | 0.198976558 | NA | NA |
| DO45171 | ICGC | C | NA | 0.729378433 | 0.877152845 | 0.340172076 | NA | NA |
| DO45173 | ICGC | C | NA | 0.663370626 | 0.821076775 | 0.25804321 | NA | NA |
| DO45175 | ICGC | C | NA | 0.643583806 | 0.81147255 | 0.183643289 | NA | NA |
| DO45177 | ICGC | A | NA | 0.615737924 | 0.837545022 | 0.138895463 | NA | NA |
| DO45179 | ICGC | C | NA | 0.698119974 | 0.846350486 | 0.323468557 | NA | NA |
| DO45181 | ICGC | A | NA | 0.63227844 | 0.822022966 | 0.186389506 | NA | NA |
| DO45183 | ICGC | C | NA | 0.719967393 | 0.837048484 | 0.393330988 | NA | NA |
| DO45185 | ICGC | B | NA | 0.78733255 | 0.90211086 | 0.47546828 | NA | NA |
| DO45187 | ICGC | C | NA | 0.609923485 | 0.70422919 | 0.116492232 | NA | NA |
| DO45189 | ICGC | C | NA | 0.623336732 | 0.750737588 | 0.157355917 | NA | NA |
| DO45191 | ICGC | A | NA | 0.662997993 | 0.782774089 | 0.301376912 | NA | NA |
| DO45193 | ICGC | B | NA | 0.753927334 | 0.874906897 | 0.417331449 | NA | NA |
| DO45195 | ICGC | A | NA | 0.653620067 | 0.848484939 | 0.239927402 | NA | NA |
| DO45197 | ICGC | A | NA | 0.614939942 | 0.834068116 | 0.065699383 | NA | NA |
| DO45199 | ICGC | C | NA | 0.67514238 | 0.869019468 | 0.183883807 | NA | NA |
| DO45201 | ICGC | C | NA | 0.631605397 | 0.847453263 | 0.184785733 | NA | NA |
| DO45203 | ICGC | C | NA | 0.697514884 | 0.864555448 | 0.335547959 | NA | NA |
| DO45205 | ICGC | B | NA | 0.733503569 | 0.840777002 | 0.373809171 | NA | NA |
| DO45207 | ICGC | A | NA | 0.626009055 | 0.806100422 | 0.186551299 | NA | NA |
| DO45209 | ICGC | B | NA | 0.747115518 | 0.845720522 | 0.356258012 | NA | NA |
| DO45211 | ICGC | B | NA | 0.812342532 | 0.886229175 | 0.520927511 | NA | NA |
| DO45213 | ICGC | C | NA | 0.683261657 | 0.818715162 | 0.331532918 | NA | NA |
| DO45215 | ICGC | A | NA | 0.627023431 | 0.80677753 | 0.238206263 | NA | NA |
| DO45217 | ICGC | B | NA | 0.640138347 | 0.720928636 | 0.211874567 | NA | NA |
| DO45219 | ICGC | C | NA | 0.708021001 | 0.848755826 | 0.291240599 | NA | NA |
| DO45221 | ICGC | B | NA | 0.749988863 | 0.887009538 | 0.368106673 | NA | NA |
| DO45223 | ICGC | C | NA | 0.680271233 | 0.843453266 | 0.325410269 | NA | NA |
| DO45225 | ICGC | C | NA | 0.618447079 | 0.773654512 | 0.1557223 | NA | NA |
| DO45227 | ICGC | C | NA | 0.585113054 | 0.768461106 | 0 | NA | NA |
| DO45229 | ICGC | A | NA | 0.64085034 | 0.853288989 | 0.153826285 | NA | NA |
| DO45231 | ICGC | A | NA | 0.603624314 | 0.773462322 | 0.098013985 | NA | NA |
| DO45233 | ICGC | A | NA | 0.641206706 | 0.836182675 | 0.180318851 | NA | NA |
| DO45235 | ICGC | A | NA | 0.709736704 | 0.855775903 | 0.314292723 | NA | NA |
| DO45237 | ICGC | A | NA | 0.682303646 | 0.863588621 | 0.225017575 | NA | NA |
| DO45239 | ICGC | C | NA | 0.629383078 | 0.766600944 | 0.174967115 | NA | NA |
| DO45241 | ICGC | C | NA | 0.599439579 | 0.793839299 | 0.088185416 | NA | NA |
| DO45243 | ICGC | C | NA | 0.639281039 | 0.812631934 | 0.232234913 | NA | NA |
| DO45245 | ICGC | B | NA | 0.725342042 | 0.80847287 | 0.36546501 | NA | NA |
| DO45247 | ICGC | C | NA | 0.586207833 | 0.723845403 | 0.082210373 | NA | NA |
| DO45249 | ICGC | B | NA | 0.71858349 | 0.829857425 | 0.381590028 | NA | NA |
| DO45251 | ICGC | C | NA | 0.646540715 | 0.744107491 | 0.275042836 | NA | NA |
| DO45253 | ICGC | C | NA | 0.613650123 | 0.77123033 | 0.110031363 | NA | NA |
| DO45255 | ICGC | A | NA | 0.670305839 | 0.87341144 | 0.22773286 | NA | NA |
| DO45257 | ICGC | C | NA | 0.628356442 | 0.728082334 | 0.189743171 | NA | NA |
| DO45259 | ICGC | B | NA | 0.708811282 | 0.806683684 | 0.35524241 | NA | NA |
| DO45261 | ICGC | C | NA | 0.688101448 | 0.893687087 | 0.294378191 | NA | NA |
| DO45263 | ICGC | C | NA | 0.685452387 | 0.783850923 | 0.316973407 | NA | NA |
| DO45265 | ICGC | C | NA | 0.70137333 | 0.825697059 | 0.308631201 | NA | NA |
| DO45267 | ICGC | C | NA | 0.683072256 | 0.87096254 | 0.192914598 | NA | NA |
| DO45269 | ICGC | B | NA | 0.692165293 | 0.779576495 | 0.211185732 | NA | NA |
| DO45273 | ICGC | B | NA | 0.691880882 | 0.831405673 | 0.318417874 | NA | NA |
| DO45275 | ICGC | B | NA | 0.770254634 | 0.885076848 | 0.417958827 | NA | NA |
| DO45277 | ICGC | B | NA | 0.714596239 | 0.850627587 | 0.394640599 | NA | NA |
| DO45279 | ICGC | C | NA | 0.647312432 | 0.742215511 | 0.341477112 | NA | NA |
| DO45281 | ICGC | A | NA | 0.636805698 | 0.800212988 | 0.199586426 | NA | NA |
| DO45283 | ICGC | B | NA | 0.752396542 | 0.885059079 | 0.315399077 | NA | NA |
| DO45285 | ICGC | B | NA | 0.745516168 | 0.89062002 | 0.444636259 | NA | NA |
| DO45287 | ICGC | B | NA | 0.744572288 | 0.869363321 | 0.394830527 | NA | NA |
| DO45289 | ICGC | B | NA | 0.725748313 | 0.862326376 | 0.415785186 | NA | NA |
| DO45297 | ICGC | B | NA | 0.746697877 | 0.83000625 | 0.485334352 | NA | NA |
| DO45299 | ICGC | B | NA | 0.871071514 | 1 | 0.612010705 | NA | NA |
| DO45301 | ICGC | B | NA | 0.649146983 | 0.805192795 | 0.182992963 | NA | NA |
| DO45303 | ICGC | B | NA | 0.780221424 | 0.865828501 | 0.437177988 | NA | NA |
| DO45305 | ICGC | B | NA | 0.735334023 | 0.802742691 | 0.3941764 | NA | NA |
| DO45307 | ICGC | B | NA | 0.723149527 | 0.832085824 | 0.400023536 | NA | NA |
| DO48672 | ICGC | C | NA | 0.671977905 | 0.833702594 | 0.23434856 | NA | NA |
| DO48674 | ICGC | A | NA | 0.677346667 | 0.854293073 | 0.273491929 | NA | NA |
| DO48677 | ICGC | C | NA | 0.639274866 | 0.80605859 | 0.159465556 | NA | NA |
| DO48679 | ICGC | C | NA | 0.732000903 | 0.885574122 | 0.390855557 | NA | NA |
| DO48681 | ICGC | C | NA | 0.626540041 | 0.733205877 | 0.244760364 | NA | NA |
| DO48682 | ICGC | C | NA | 0.766938605 | 0.889468495 | 0.411662331 | NA | NA |
| DO48693 | ICGC | C | NA | 0.67998958 | 0.849476564 | 0.26983347 | NA | NA |
| DO48695 | ICGC | C | NA | 0.700576158 | 0.884093668 | 0.290576661 | NA | NA |
| DO48697 | ICGC | B | NA | 0.717861367 | 0.82403596 | 0.408389821 | NA | NA |
| DO48700 | ICGC | B | NA | 0.685544428 | 0.810781446 | 0.322571589 | NA | NA |
| DO48701 | ICGC | C | NA | 0.611528365 | 0.797354297 | 0.08862318 | NA | NA |
| DO48704 | ICGC | C | NA | 0.726051348 | 0.855136337 | 0.362080008 | NA | NA |
| DO48706 | ICGC | C | NA | 0.67825933 | 0.838888028 | 0.207768182 | NA | NA |
| DO48712 | ICGC | A | NA | 0.662528177 | 0.806733995 | 0.264262433 | NA | NA |
| DO48715 | ICGC | C | NA | 0.639469091 | 0.772425918 | 0.196571232 | NA | NA |
| DO48716 | ICGC | A | NA | 0.679830556 | 0.843080634 | 0.254657063 | NA | NA |
| DO48717 | ICGC | B | NA | 0.707349697 | 0.855562725 | 0.308610689 | NA | NA |
| DO48719 | ICGC | C | NA | 0.695437294 | 0.892226254 | 0.293993188 | NA | NA |
| DO48720 | ICGC | C | NA | 0.636272073 | 0.790485651 | 0.205908657 | NA | NA |
| DO48721 | ICGC | C | NA | 0.687877995 | 0.901025202 | 0.229051348 | NA | NA |
| DO48723 | ICGC | B | NA | 0.721532834 | 0.865688406 | 0.313514035 | NA | NA |
| DO48725 | ICGC | B | NA | 0.693429283 | 0.861388897 | 0.285676207 | NA | NA |
| DO48727 | ICGC | A | NA | 0.615927925 | 0.798845717 | 0.133916035 | NA | NA |
| DO48728 | ICGC | C | NA | 0.698309416 | 0.819433367 | 0.345217486 | NA | NA |
| DO48730 | ICGC | B | NA | 0.73652607 | 0.839520576 | 0.356062679 | NA | NA |
| DO48732 | ICGC | C | NA | 0.688185983 | 0.822347675 | 0.283966461 | NA | NA |
| DO48733 | ICGC | C | NA | 0.654102012 | 0.841929595 | 0.236587858 | NA | NA |
| DO48736 | ICGC | C | NA | 0.626703318 | 0.723922083 | 0.208839081 | NA | NA |
| DO48737 | ICGC | B | NA | 0.717667074 | 0.831253461 | 0.410138757 | NA | NA |
| DO48741 | ICGC | B | NA | 0.740898257 | 0.91339477 | 0.348988986 | NA | NA |
| DO48742 | ICGC | A | NA | 0.689131692 | 0.876569608 | 0.272196875 | NA | NA |
| DO48743 | ICGC | A | NA | 0.633043033 | 0.783051315 | 0.165867268 | NA | NA |
| DO48746 | ICGC | C | NA | 0.634986232 | 0.806975423 | 0.144583328 | NA | NA |
| DO48747 | ICGC | B | NA | 0.681465024 | 0.83150186 | 0.279334156 | NA | NA |
| DO48751 | ICGC | C | NA | 0.743853553 | 0.922221238 | 0.308110555 | NA | NA |
| DO48757 | ICGC | B | NA | 0.732150505 | 0.81782052 | 0.334586991 | NA | NA |
| DO48759 | ICGC | C | NA | 0.683855144 | 0.888638877 | 0.244113529 | NA | NA |
| DO48760 | ICGC | A | NA | 0.675193169 | 0.863224526 | 0.262125003 | NA | NA |
| DO48761 | ICGC | A | NA | 0.657761233 | 0.807989991 | 0.281324275 | NA | NA |
| DO50774 | ICGC | A | NA | 0.654751603 | 0.867552349 | 0.194876117 | NA | NA |
| DO50776 | ICGC | C | NA | 0.653967953 | 0.821962872 | 0.246557835 | NA | NA |
| DO50778 | ICGC | B | NA | 0.777061267 | 0.926571106 | 0.444213405 | NA | NA |
| DO50783 | ICGC | C | NA | 0.687498779 | 0.845068533 | 0.275728759 | NA | NA |
| DO50785 | ICGC | B | NA | 0.702498396 | 0.77700947 | 0.2626625 | NA | NA |
| DO50787 | ICGC | A | NA | 0.681771098 | 0.858655093 | 0.26533954 | NA | NA |
| DO50789 | ICGC | A | NA | 0.659224288 | 0.85630994 | 0.196650835 | NA | NA |
| DO50791 | ICGC | B | NA | 0.732970749 | 0.877237545 | 0.328678223 | NA | NA |
| DO50793 | ICGC | C | NA | 0.69664468 | 0.868656535 | 0.27621519 | NA | NA |
| DO50798 | ICGC | C | NA | 0.609139556 | 0.849537841 | 0.049854032 | NA | NA |
| DO50799 | ICGC | B | NA | 0.7606606 | 0.835142665 | 0.368523 | NA | NA |
| DO50800 | ICGC | A | NA | 0.625829868 | 0.793906465 | 0.237462293 | NA | NA |
| DO50802 | ICGC | C | NA | 0.700529914 | 0.89093191 | 0.246212899 | NA | NA |
| DO50803 | ICGC | A | NA | 0.661241916 | 0.851561078 | 0.207590529 | NA | NA |
| DO50804 | ICGC | C | NA | 0.669835962 | 0.848178965 | 0.20054492 | NA | NA |
| DO50805 | ICGC | C | NA | 0.699924434 | 0.840411025 | 0.321110742 | NA | NA |
| DO50806 | ICGC | B | NA | 0.723365824 | 0.847127484 | 0.310893547 | NA | NA |
| DO50807 | ICGC | A | NA | 0.560920407 | 0.75210287 | 0.039170985 | NA | NA |
| DO50808 | ICGC | C | NA | 0.653396918 | 0.834015197 | 0.217385279 | NA | NA |
| DO50809 | ICGC | C | NA | 0.722569647 | 0.87152616 | 0.307707784 | NA | NA |
| DO50811 | ICGC | C | NA | 0.626582034 | 0.764537188 | 0.159621998 | NA | NA |
| DO50813 | ICGC | C | NA | 0.647919695 | 0.850557887 | 0.205691989 | NA | NA |
| DO50814 | ICGC | C | NA | 0.654001702 | 0.838768581 | 0.192092868 | NA | NA |
| DO50815 | ICGC | B | NA | 0.712245856 | 0.78930461 | 0.384854147 | NA | NA |
| DO50816 | ICGC | C | NA | 0.587055613 | 0.80172819 | 0.070748941 | NA | NA |
| DO50817 | ICGC | A | NA | 0.618912618 | 0.79429773 | 0.144886244 | NA | NA |
| DO50818 | ICGC | A | NA | 0.631391919 | 0.820740416 | 0.170495894 | NA | NA |
| DO50819 | ICGC | A | NA | 0.685877768 | 0.856438804 | 0.273468611 | NA | NA |
| DO50820 | ICGC | A | NA | 0.634433639 | 0.736407032 | 0.232228497 | NA | NA |
| DO50822 | ICGC | C | NA | 0.675252462 | 0.845938323 | 0.209237643 | NA | NA |
| DO50825 | ICGC | C | NA | 0.675728638 | 0.877112536 | 0.26473218 | NA | NA |
| DO50829 | ICGC | B | NA | 0.717083536 | 0.85447566 | 0.261917548 | NA | NA |
| DO50832 | ICGC | A | NA | 0.670175139 | 0.808697883 | 0.285450594 | NA | NA |
| DO50839 | ICGC | C | NA | 0.629702929 | 0.782445543 | 0.176139337 | NA | NA |
| DO50840 | ICGC | B | NA | 0.79208519 | 0.913311107 | 0.501109087 | NA | NA |
| DO50844 | ICGC | C | NA | 0.704106375 | 0.827922609 | 0.288160958 | NA | NA |
| DO50845 | ICGC | C | NA | 0.614253974 | 0.718691337 | 0.24406361 | NA | NA |
| DO50850 | ICGC | C | NA | 0.640103462 | 0.856919052 | 0.132423855 | NA | NA |
| DO50855 | ICGC | B | NA | 0.652871342 | 0.774056157 | 0.260513919 | NA | NA |
| DO50857 | ICGC | C | NA | 0.719039996 | 0.895185983 | 0.332229694 | NA | NA |
| DO50859 | ICGC | C | NA | 0.755475338 | 0.900262317 | 0.394433235 | NA | NA |
| DO227643 | ICGC | A | NA | 0.641662043 | 0.855474245 | 0.182874441 | NA | NA |
| DO227801 | ICGC | C | NA | 0.663006361 | 0.851970696 | 0.234701707 | NA | NA |
