# Supplementary material for: Identification of HCC Subtypes With Different Prognosis and Metabolic Patterns Based on Mitophagy
Source: Front Cell Dev Biol. 2021 Dec 16;9:799507. doi: 10.3389/fcell.2021.799507 (PMC8716756; doi:10.3389/fcell.2021.799507)
Supplement: Supplementary file 8 [file Table3.DOCX]

| **Data**  **base** | **ID** | **Description** | **geneID** | **count** | **Pvalue-clusterProfiler** | **p.adjust-clusterProfiler** | **Qvalue-clusterProfiler** | **LogP-Metascape** | **Log qvalue-Metascape** |
| --- | --- | --- | --- | --- | --- | --- | --- | --- | --- |
| KEGG | hsa00830 | Retinol metabolism | ADH1C/CYP2A6/CYP4A11/CYP2C8/CYP4A22/RDH16/CYP2C9/CYP3A4/ADH4/CYP1A2 | 10 | 3.39E-07 | 4.89E-05 | 4.04E-05 | -9.5 | -7.1 |
|  | hsa04512 | ECM-receptor interaction | LAMC1/ITGAV/LAMB1/COL4A1/COL6A3/ITGB4/SPP1/ITGA3/THBS1/COL1A2/COL1A1 | 11 | 5.47E-07 | 4.89E-05 | 4.04E-05 | -9.7 | -7.2 |
|  | hsa04976 | Bile secretion | SLC22A1/SLC27A5/SLC10A1/CYP3A4/AQP9/CYP7A1/ATP1A1/ATP1B3/SLC2A1/FXYD2 | 10 | 6.77E-07 | 4.89E-05 | 4.04E-05 | -9 | -6.6 |
|  | hsa04974 | Protein digestion and absorption | ATP1A1/COL5A2/ATP1B3/COL4A1/COL6A3/SLC1A5/COL1A2/COL5A1/COL3A1/COL1A1/FXYD2 | 11 | 1.20E-06 | 6.49E-05 | 5.35E-05 | -10 | -7.8 |
|  | hsa03320 | PPAR signaling pathway | SLC27A5/APOC3/CYP4A11/PCK1/CYP4A22/CYP8B1/APOA1/FABP1/CYP7A1 | 9 | 9.63E-06 | 0.000417805 | 0.00034454 | -7.2 | -5.1 |
|  | hsa04110 | Cell cycle | BUB1B/MAD2L1/MCM2/MCM4/PLK1/CDC6/CDK1/CCNB1/CCNB2/CDC20/PTTG1 | 11 | 1.64E-05 | 0.000593026 | 0.000489035 | -8.7 | -6.4 |
|  | hsa00982 | Drug metabolism - cytochrome P450 | ADH1C/CYP2A6/CYP2C8/CYP2C9/CYP3A4/ADH4/CYP2E1/CYP1A2 | 8 | 4.89E-05 | 0.001515482 | 0.00124973 | -6.8 | -4.7 |
|  | hsa00980 | Metabolism of xenobiotics by cytochrome P450 | ADH1C/CYP2A6/AKR7A3/CYP2C9/CYP3A4/ADH4/CYP2E1/CYP1A2 | 8 | 7.24E-05 | 0.001763728 | 0.001454445 | -6.5 | -4.5 |
|  | hsa04510 | Focal adhesion | SRC/LAMC1/ITGAV/LAMB1/FLNA/COL4A1/COL6A3/ITGB4/SPP1/ITGA3/THBS1/COL1A2/COL1A1 | 13 | 7.32E-05 | 0.001763728 | 0.001454445 | -8.2 | -5.9 |
|  | hsa05204 | Chemical carcinogenesis | ADH1C/CYP2A6/CYP2C8/CYP2C9/CYP3A4/ADH4/CYP2E1/CYP1A2 | 8 | 0.000124643 | 0.002704743 | 0.002230445 | -7.1 | -5 |
|  | hsa00590 | Arachidonic acid metabolism | CYP4A11/CYP2C8/CYP4A22/CYP2C9/CYP2E1/ALOX5/GPX7 | 7 | 0.000146874 | 0.002897414 | 0.00238933 | -5.9 | -3.9 |
|  | hsa00591 | Linoleic acid metabolism | CYP2C8/CYP2C9/CYP3A4/CYP2E1/CYP1A2 | 5 | 0.000165775 | 0.002997773 | 0.00247209 | -5.3 | -3.4 |
|  | hsa00010 | Glycolysis / Gluconeogenesis | ADH1C/PCK1/ALDOB/ADH4/ENO2/PFKP/HK2 | 7 | 0.000238332 | 0.003978306 | 0.00328068 | -5.1 | -3.2 |
|  | hsa01230 | Biosynthesis of amino acids | TAT/SDS/ALDOB/ENO2/PFKP/ASNS/PYCR1 | 7 | 0.000438279 | 0.006793327 | 0.005602065 | -4.3 | -2.6 |
|  | hsa04964 | Proximal tubule bicarbonate reclamation | PCK1/ATP1A1/ATP1B3/FXYD2 | 4 | 0.000749109 | 0.010837109 | 0.008936738 | -4.3 | -2.6 |
|  | hsa04668 | TNF signaling pathway | JAG1/MMP14/MAPK13/CXCL5/LIF/MMP9/CCL20/CXCL1 | 8 | 0.001045097 | 0.014174134 | 0.01168859 | -6.1 | -4.1 |
|  | hsa05230 | Central carbon metabolism in cancer | G6PD/SLC1A5/PFKP/SLC2A1/HK2/SLC16A3 | 6 | 0.001639835 | 0.02093201 | 0.01726142 | -5.3 | -3.4 |
|  | hsa04114 | Oocyte meiosis | MAD2L1/PLK1/CDK1/CCNB1/CCNB2/CDC20/MAPK13/PTTG1 | 8 | 0.002337404 | 0.028178705 | 0.023237351 | -3.9 | -2.2 |
|  | hsa05146 | Amoebiasis | LAMC1/LAMB1/COL4A1/COL1A2/COL3A1/COL1A1/CXCL1 | 7 | 0.002698893 | 0.030824198 | 0.025418936 | -5.4 | -3.4 |
|  | hsa00051 | Fructose and mannose metabolism | ALDOB/PFKFB3/PFKP/HK2 | 4 | 0.003000909 | 0.032559868 | 0.026850243 | -3.3 | -1.7 |
| GOBP | GO:0043062 | extracellular structure organization | TTR/APOC3/LPA/APOA1/LAMC1/ITGAV/LAMB1/COL5A2/MMP14/DDR1/COL4A1/COL6A3/ITGB4/SPP1/ITGA3/MMP11/SOX9/THBS1/MMP12/COL1A2/COL5A1/COL3A1/FBLN1/COL16A1/MFAP2/VCAN/SULF1/COL1A1/MMP9/SPINT1/MMP7 | 31 | 2.62E-15 | 8.99E-12 | 7.37E-12 | -13 | -10 |
|  | GO:0030198 | extracellular matrix organization | TTR/LAMC1/ITGAV/LAMB1/COL5A2/MMP14/DDR1/COL4A1/COL6A3/ITGB4/SPP1/ITGA3/MMP11/SOX9/THBS1/MMP12/COL1A2/COL5A1/COL3A1/FBLN1/COL16A1/MFAP2/VCAN/SULF1/COL1A1/MMP9/SPINT1/MMP7 | 28 | 2.04E-14 | 3.51E-11 | 2.88E-11 | -13 | -10 |
|  | GO:0140014 | mitotic nuclear division | RAN/ANLN/BUB1B/KIF23/MAD2L1/PRC1/TRIP13/PLK1/TPX2/CDC6/ZWINT/CCNB1/KIF2C/CDCA5/MYBL2/FLNA/CDC20/UBE2C/AURKB/SPHK1 | 20 | 1.13E-10 | 1.30E-07 | 1.06E-07 | -15 | -11 |
|  | GO:0031589 | cell-substrate adhesion | HRG/APOA1/SRC/ADAM9/LAMC1/ITGAV/LAMB1/TYRO3/JAG1/MMP14/FLNA/DDR1/ITGB4/ITGA3/THBS1/MMP12/COL3A1/FBLN1/COL16A1/COL1A1/POSTN/AGR2 | 22 | 7.53E-10 | 6.47E-07 | 5.30E-07 | -12 | -8.9 |
|  | GO:0000070 | mitotic sister chromatid segregation | RAN/BUB1B/KIF23/MAD2L1/PRC1/TRIP13/PLK1/CDC6/ZWINT/CCNB1/KIF2C/CDCA5/CDC20/AURKB | 14 | 4.02E-09 | 2.76E-06 | 2.26E-06 | -14 | -11 |
|  | GO:0000280 | nuclear division | RAN/ANLN/BUB1B/KIF23/MAD2L1/TOP2A/PRC1/TRIP13/PLK1/TPX2/CDC6/ZWINT/CCNB1/KIF2C/CDCA5/MYBL2/FLNA/CDC20/UBE2C/AURKB/SPHK1/LIF | 22 | 5.12E-09 | 2.93E-06 | 2.40E-06 | -14 | -10 |
|  | GO:0048285 | organelle fission | RAN/ANLN/BUB1B/KIF23/MAD2L1/TOP2A/PRC1/TRIP13/PLK1/TPX2/CDC6/ZWINT/CCNB1/KIF2C/CDCA5/MYBL2/FLNA/CDC20/UBE2C/AURKB/SPHK1/LIF | 22 | 3.18E-08 | 1.44E-05 | 1.18E-05 | -13 | -9.5 |
|  | GO:0010951 | negative regulation of endopeptidase activity | LPA/C3P1/FETUB/SPP2/FABP1/SRC/BIRC5/COL6A3/PLAUR/PAPLN/PTTG1/THBS1/VIL1/MMP9/SPINT1/PCSK1N | 16 | 3.71E-08 | 1.44E-05 | 1.18E-05 | -12 | -9 |
|  | GO:0000819 | sister chromatid segregation | RAN/BUB1B/KIF23/MAD2L1/PRC1/TRIP13/PLK1/CDC6/ZWINT/CCNB1/KIF2C/CDCA5/CDC20/AURKB | 14 | 3.77E-08 | 1.44E-05 | 1.18E-05 | -14 | -10 |
|  | GO:0007059 | chromosome segregation | RAN/ECT2/BUB1B/KIF23/MAD2L1/TOP2A/PRC1/TRIP13/PLK1/CDC6/NUF2/ZWINT/CCNB1/KIF2C/CDCA5/CDC20/BIRC5/AURKB | 18 | 5.37E-08 | 1.85E-05 | 1.51E-05 | -11 | -8.5 |
|  | GO:0051983 | regulation of chromosome segregation | ECT2/BUB1B/MAD2L1/TRIP13/PLK1/CDC6/CCNB1/KIF2C/CDCA5/CDC20/AURKB | 11 | 6.18E-08 | 1.93E-05 | 1.58E-05 | -11 | -7.9 |
|  | GO:1902850 | microtubule cytoskeleton organization involved in mitosis | RAN/KIF23/MAD2L1/PRC1/ARHGEF2/PLK1/TPX2/CCNB1/MYBL2/FLNA/CDC20/AURKB | 12 | 7.01E-08 | 2.01E-05 | 1.65E-05 | -8.1 | -5.9 |
|  | GO:0010466 | negative regulation of peptidase activity | LPA/C3P1/FETUB/SPP2/FABP1/SRC/BIRC5/COL6A3/PLAUR/PAPLN/PTTG1/THBS1/VIL1/MMP9/SPINT1/PCSK1N | 16 | 8.58E-08 | 2.27E-05 | 1.86E-05 | -12 | -8.8 |
|  | GO:0007091 | metaphase/anaphase transition of mitotic cell cycle | BUB1B/MAD2L1/TRIP13/PLK1/CDC6/CCNB1/CDC20/AURKB | 8 | 2.31E-07 | 5.28E-05 | 4.33E-05 | -9.9 | -7.3 |
|  | GO:0010965 | regulation of mitotic sister chromatid separation | BUB1B/MAD2L1/TRIP13/PLK1/CDC6/CCNB1/CDC20/AURKB | 8 | 2.31E-07 | 5.28E-05 | 4.33E-05 | -11 | -8.2 |
|  | GO:0044784 | metaphase/anaphase transition of cell cycle | BUB1B/MAD2L1/TRIP13/PLK1/CDC6/CCNB1/CDC20/AURKB | 8 | 3.23E-07 | 6.53E-05 | 5.35E-05 | -9.6 | -7.2 |
|  | GO:0051306 | mitotic sister chromatid separation | BUB1B/MAD2L1/TRIP13/PLK1/CDC6/CCNB1/CDC20/AURKB | 8 | 3.23E-07 | 6.53E-05 | 5.35E-05 | -11 | -8.1 |
|  | GO:0042738 | exogenous drug catabolic process | CYP2A6/CYP2C8/CYP2C9/CYP3A4/CYP1A2 | 5 | 3.50E-07 | 6.69E-05 | 5.48E-05 | -9 | -7.3 |
|  | GO:0098813 | nuclear chromosome segregation | RAN/ECT2/BUB1B/KIF23/MAD2L1/PRC1/TRIP13/PLK1/CDC6/ZWINT/CCNB1/KIF2C/CDCA5/CDC20/AURKB | 15 | 4.23E-07 | 7.65E-05 | 6.28E-05 | -12 | -9.1 |
|  | GO:0072330 | monocarboxylic acid biosynthetic process | SLC27A5/APOC3/CYP2C8/CYP8B1/CYP2C9/SDS/ALDOB/CYP3A4/CYP2E1/CYP7A1/CYP1A2/PFKFB3/ENO2/PFKP/ALOX5/HK2/ASNS/VCAN | 18 | 4.69E-07 | 7.97E-05 | 6.54E-05 | -7.3 | -5.1 |
| GOCC | GO:0031012 | extracellular matrix | HPX/HRG/SERPINC1/APOC3/F9/SPP2/APOA1/LAMC1/LAMB1/ANXA5/COL5A2/MMP14/COL4A1/COL6A3/CTSC/MMP11/SERPINE2/THBS1/MMP12/COL1A2/CTHRC1/COL5A1/COL3A1/FBLN1/COL16A1/MFAP2/VCAN/SULF1/COL1A1/MMP9/S100A9/POSTN/MMP7 | 33 | 5.53E-15 | 1.86E-12 | 1.48E-12 | -19 | -15 |
|  | GO:0005788 | endoplasmic reticulum lumen | SERPINC1/F9/SPP2/APOA1/LAMC1/CKAP4/LAMB1/COL5A2/COL4A1/COL6A3/TMEM132A/PLAUR/CTSC/PRSS23/SPP1/GPX7/THBS1/GOLM1/COL1A2/COL5A1/COL3A1/FKBP10/COL16A1/VCAN/COL1A1/AFP | 26 | 6.95E-14 | 1.17E-11 | 9.32E-12 | -17 | -14 |
|  | GO:0062023 | collagen-containing extracellular matrix | HPX/HRG/SERPINC1/APOC3/F9/SPP2/APOA1/LAMC1/LAMB1/ANXA5/COL5A2/COL4A1/COL6A3/CTSC/SERPINE2/THBS1/COL1A2/CTHRC1/COL5A1/COL3A1/FBLN1/COL16A1/MFAP2/VCAN/SULF1/COL1A1/MMP9/S100A9/POSTN | 29 | 1.29E-13 | 1.45E-11 | 1.16E-11 | -18 | -14 |
|  | GO:0044420 | extracellular matrix component | LAMC1/LAMB1/COL5A2/COL4A1/COL1A2/COL5A1/COL3A1/FBLN1/MFAP2/COL1A1 | 10 | 5.17E-10 | 4.34E-08 | 3.47E-08 | -10.1 | -9.8 |
|  | GO:0098644 | complex of collagen trimers | COL5A2/COL4A1/COL1A2/COL5A1/COL3A1/COL1A1 | 6 | 8.79E-08 | 5.90E-06 | 4.72E-06 | -9.5 | -7 |
|  | GO:0005583 | fibrillar collagen trimer | COL5A2/COL1A2/COL5A1/COL3A1/COL1A1 | 5 | 1.79E-07 | 8.57E-06 | 6.85E-06 | -9.5 | -7 |
|  | GO:0098643 | banded collagen fibril | COL5A2/COL1A2/COL5A1/COL3A1/COL1A1 | 5 | 1.79E-07 | 8.57E-06 | 6.85E-06 | -9.5 | -7 |
|  | GO:0034358 | plasma lipoprotein particle | HPR/PON1/APOF/APOC3/LPA/APOA1/SAA1 | 7 | 5.17E-07 | 1.93E-05 | 1.54E-05 | -7.7 | -5.5 |
|  | GO:1990777 | lipoprotein particle | HPR/PON1/APOF/APOC3/LPA/APOA1/SAA1 | 7 | 5.17E-07 | 1.93E-05 | 1.54E-05 | -7.7 | -5.5 |
|  | GO:0032994 | protein-lipid complex | HPR/PON1/APOF/APOC3/LPA/APOA1/SAA1 | 7 | 7.55E-07 | 2.54E-05 | 2.03E-05 | -7.4 | -5.2 |
|  | GO:0034364 | high-density lipoprotein particle | HPR/PON1/APOF/APOC3/APOA1/SAA1 | 6 | 9.96E-07 | 3.04E-05 | 2.43E-05 | -7 | -4.9 |
|  | GO:0005581 | collagen trimer | COL5A2/COL4A1/COL6A3/COL1A2/CTHRC1/COL5A1/COL3A1/COL16A1/COL1A1 | 9 | 2.24E-06 | 6.27E-05 | 5.01E-05 | -7.2 | -5 |
|  | GO:0072562 | blood microparticle | CFHR3/HPR/HP/PON1/HPX/HRG/SERPINC1/AFM/APOA1/ANXA5/SLC2A1 | 11 | 4.73E-06 | 0.000122159 | 9.76E-05 | -6.2 | -4.2 |
|  | GO:0005890 | sodium:potassium-exchanging ATPase complex | FXYD1/ATP1A1/ATP1B3/FXYD2 | 4 | 9.60E-06 | 0.000230495 | 0.000184136 | -5.6 | -3.7 |
|  | GO:0000776 | kinetochore | BUB1B/MAD2L1/PLK1/NUF2/ZWINT/CCNB1/KIF2C/BIRC5/AURKB/CENPM | 10 | 1.08E-05 | 0.00024131 | 0.000192776 | -6.6 | -4.5 |
|  | GO:0000779 | condensed chromosome, centromeric region | BUB1B/MAD2L1/PLK1/ZWINT/CCNB1/KIF2C/BIRC5/AURKB/CENPM | 9 | 1.71E-05 | 0.00035936 | 0.000287083 | -6.3 | -4.3 |
|  | GO:0090533 | cation-transporting ATPase complex | FXYD1/ATP1A1/ATP1B3/FXYD2 | 4 | 2.82E-05 | 0.00055783 | 0.000445635 | -4.8 | -3 |
|  | GO:0000775 | chromosome, centromeric region | BUB1B/MAD2L1/PLK1/NUF2/ZWINT/CCNB1/KIF2C/CDCA5/BIRC5/AURKB/CENPM | 11 | 4.12E-05 | 0.000768137 | 0.000613643 | -6.8 | -4.7 |
|  | GO:0000777 | condensed chromosome kinetochore | BUB1B/MAD2L1/PLK1/ZWINT/CCNB1/KIF2C/BIRC5/CENPM | 8 | 5.93E-05 | 0.001049403 | 0.000838339 | -6.6 | -3.6 |
|  | GO:0000793 | condensed chromosome | BUB1B/MAD2L1/TOP2A/PLK1/ZWINT/CCNB1/KIF2C/CDCA5/BIRC5/AURKB/CENPM | 11 | 7.96E-05 | 0.001338006 | 0.001068896 | -5 | -3.1 |
| GOMF | GO:0005201 | extracellular matrix structural constituent | LAMC1/LAMB1/COL5A2/COL4A1/COL6A3/THBS1/COL1A2/CTHRC1/COL5A1/COL3A1/FBLN1/COL16A1/MFAP2/VCAN/COL1A1/POSTN | 16 | 1.03E-09 | 4.83E-07 | 3.86E-07 | -12 | -8.7 |
|  | GO:0061134 | peptidase regulator activity | HRG/SERPINC1/LPA/C3P1/FETUB/SPP2/BIRC5/COL6A3/CTSC/PAPLN/PTTG1/SERPINE2/FBLN1/VIL1/SPINT1/PCSK1N | 16 | 1.75E-08 | 4.10E-06 | 3.27E-06 | -9.7 | -7.2 |
|  | GO:0004866 | endopeptidase inhibitor activity | HRG/SERPINC1/LPA/C3P1/FETUB/SPP2/BIRC5/COL6A3/PAPLN/PTTG1/SERPINE2/VIL1/SPINT1/PCSK1N | 14 | 3.07E-08 | 4.80E-06 | 3.83E-06 | -9.2 | -6.8 |
|  | GO:0061135 | endopeptidase regulator activity | HRG/SERPINC1/LPA/C3P1/FETUB/SPP2/BIRC5/COL6A3/PAPLN/PTTG1/SERPINE2/VIL1/SPINT1/PCSK1N | 14 | 4.67E-08 | 5.48E-06 | 4.38E-06 | -8.8 | -6.4 |
|  | GO:0030414 | peptidase inhibitor activity | HRG/SERPINC1/LPA/C3P1/FETUB/SPP2/BIRC5/COL6A3/PAPLN/PTTG1/SERPINE2/VIL1/SPINT1/PCSK1N | 14 | 5.96E-08 | 5.59E-06 | 4.47E-06 | -9 | -6.6 |
|  | GO:0030020 | extracellular matrix structural constituent conferring tensile strength | COL5A2/COL4A1/COL6A3/COL1A2/COL5A1/COL3A1/COL16A1/COL1A1 | 8 | 1.00E-07 | 7.83E-06 | 6.26E-06 | -8.7 | -6.4 |
|  | GO:0005506 | iron ion binding | CYP2A6/CYP4A11/CYP2C8/CYP4A22/CYP8B1/CYP2C9/CYP3A4/CYP2A7/CYP2E1/CYP7A1/CYP1A2/ALOX5/EGLN3 | 13 | 1.55E-07 | 1.04E-05 | 8.28E-06 | -9.1 | -6.7 |
|  | GO:0048407 | platelet-derived growth factor binding | COL4A1/COL1A2/COL5A1/COL3A1/COL1A1 | 5 | 2.49E-07 | 1.46E-05 | 1.17E-05 | -7.7 | -5.5 |
|  | GO:0004497 | monooxygenase activity | CYP2A6/CYP4A11/CYP2C8/CYP4A22/CYP2C9/CYP3A4/CYP2A7/CYP2E1/CYP7A1/CYP1A2 | 10 | 4.11E-07 | 2.14E-05 | 1.71E-05 | -8.8 | -6.5 |
|  | GO:0005178 | integrin binding | SRC/ADAM9/ITGAV/MMP14/SPP1/ITGA3/THBS1/COL5A1/COL3A1/COL16A1 | 10 | 5.15E-06 | 0.00022024 | 0.000175975 | -9.4 | -7 |
|  | GO:0008514 | organic anion transmembrane transporter activity | SLC10A1/SLC13A5/AQP9/MFSD2A/SLC25A24/SLC6A6/SLC38A1/SLC1A5/SLC2A1/SLC16A3/SLC6A8 | 11 | 5.17E-06 | 0.00022024 | 0.000175975 | -4.4 | -2.7 |
|  | GO:0004857 | enzyme inhibitor activity | HRG/SERPINC1/APOC3/LPA/C3P1/FETUB/SPP2/APOA1/ANXA5/BIRC5/COL6A3/PAPLN/PTTG1/SERPINE2/VIL1/SPINT1/PCSK1N | 17 | 6.27E-06 | 0.000235665 | 0.000188299 | -4.8 | -3.1 |
|  | GO:0050839 | cell adhesion molecule binding | RAN/ASAP1/SRC/ANLN/ADAM9/ITGAV/DSG2/CCNB2/MMP14/FLNA/DBN1/NDRG1/PFKP/SPP1/ITGA3/THBS1/COL5A1/COL3A1/COL16A1/S100P | 20 | 6.53E-06 | 0.000235665 | 0.000188299 | -11 | -8.1 |
|  | GO:0008509 | anion transmembrane transporter activity | SLC22A1/SLC10A1/FXYD1/SLC13A5/AQP9/MFSD2A/SLC25A24/SLC6A6/SLC38A1/SLC1A5/SLC2A1/SLC16A3/SLC6A8/CLDN4 | 14 | 9.79E-06 | 0.000327968 | 0.000262051 | -4.7 | -2.8 |
|  | GO:0046943 | carboxylic acid transmembrane transporter activity | SLC10A1/SLC13A5/AQP9/MFSD2A/SLC6A6/SLC38A1/SLC1A5/SLC16A3/SLC6A8 | 9 | 1.14E-05 | 0.000354929 | 0.000283593 | -4.9 | -3 |
|  | GO:0005342 | organic acid transmembrane transporter activity | SLC10A1/SLC13A5/AQP9/MFSD2A/SLC6A6/SLC38A1/SLC1A5/SLC16A3/SLC6A8 | 9 | 1.23E-05 | 0.00036151 | 0.000288851 | -5.7 | -3.8 |
|  | GO:0001968 | fibronectin binding | LPA/ITGAV/ITGA3/THBS1/FBLN1 | 5 | 1.97E-05 | 0.00054249 | 0.000433457 | -5.4 | -3.5 |
|  | GO:0020037 | heme binding | CYP2A6/HRG/CYP4A11/CYP4A22/CYP3A4/CYP2E1/CYP7A1/CYP1A2/SRC | 9 | 2.15E-05 | 0.000558948 | 0.000446606 | -9.6 | -7.1 |
|  | GO:0016705 | oxidoreductase activity, acting on paired donors, with incorporation or reduction of molecular oxygen | CYP2A6/CYP4A11/CYP2C8/CYP4A22/CYP3A4/CYP2A7/CYP2E1/CYP7A1/CYP1A2/EGLN3 | 10 | 2.82E-05 | 0.00069566 | 0.000555841 | -7.4 | -5.3 |
|  | GO:0046906 | tetrapyrrole binding | CYP2A6/HRG/CYP4A11/CYP4A22/CYP3A4/CYP2E1/CYP7A1/CYP1A2/SRC | 9 | 4.40E-05 | 0.001031836 | 0.00082445 | -9.2 | -6.8 |
