# Supplementary material for: Identification of HCC Subtypes With Different Prognosis and Metabolic Patterns Based on Mitophagy
Source: Front Cell Dev Biol. 2021 Dec 16;9:799507. doi: 10.3389/fcell.2021.799507 (PMC8716756; doi:10.3389/fcell.2021.799507)
Supplement: Supplementary file 9 [file Table4.DOCX]

Supplementary Table 4： 21 drugs with reported and experimentally verified the therapeutic potential from previous studies.

| Name | biological function | source |
| --- | --- | --- |
| Obatoclax.Mesylate | pan-Bcl-2 inhibitor | doi: 10.1016/j.tranon.2021.101116  doi:10.1016/j.canlet.2012.03.023 |
| Doxorubicin | interacts with the DNA and generate free radicals that induce DNA and cell membrane damage | doi:10.4103/0973-1482.139267  doi:10.1021/acsami.1c09610 |
| ABT.888(Veliparib) | inhibitors of PARP and potentiate DNA-damaging agents by blocking BER and DNA restoration | doi:10.1016/j.clinre.2020.09.014  doi:10.1007/s00280-015-2852-2 |
| Vorinostat | Histone Deacetylase inhibition | doi:10.3892/ijo.2014.2423  doi:10.1002/cam4.1278 |
| Camptothecin | inhibit topoisomerase I in tumor cells | doi:10.3389/fonc.2021.661157 |
| Shikonin | induces signaling pathways that regulate oxidative stress responses, mitochondrial function, and cytoskeleton formation | doi:10.2147/JHC.S237614  doi:10.1016/j.lfs.2020.118796  doi.org/10.1016/j.canlet.2019.04.033 |
| Epothilone.B | inhibit the depolymerization of tubulin, form stable microtubules and the division of cancer cells | doi:10.1016/j.ejmech.2018.08.055  doi:10.1159/000106450 |
| Gemcitabine | inhibits DNA synthesis through interfering with DNA chain elongation and depleting deoxynucleotide stores | doi:10.3390/pharmaceutics12100985  doi:10.1002/cam4.1138  doi:10.3389/fonc.2017.00035 |
| GDC.0449 | hedgehog (Hh) signaling pathway antagonist | doi:10.1371/journal.pone.0023943  doi:10.1177/2050640613496605  doi:10.1016/j.amjsurg.2015.03.001 |
| Vinblastine | microtubule-destabilizing agent | doi:10.14670/HH-22.285  doi:10.1016/j.bcp.2012.01.013 |
| Methotrexate | cytotoxic agent | doi:10.1080/01635581.2019.1614199 |
| ATRA | induce differentiation of several types of tumor cells | doi:10.1186/s13063-019-3349-9 |
| AZD.2281 (Olaparib) | PARP inhibitor | doi:10.1073/pnas.2002917117  doi:10.1096/fj.201903045RR  doi:10.1186/s12943-021-01315-9 |
| BI.2536 | PLK inhibitor | doi:10.2174/1871520616666160926111911 |
| MS.275 | histone deacetylase (HDAC) inhibitor | doi:10.3892/mmr.2012.858 |
| Bleomycin | lead to extended cell cycle arrest, apoptosis, and mitotic cell death | doi:10.3760/cma.j.issn.0376-2491.2018.39.008  doi:10.3390/ijms19051372 |
| IPA.3 | enhancement of apoptosis and blockage of activation of NF-κB | doi:10.1371/journal.pone.0068843 |
| Mitomycin.C | primarily alkylation and subsequent crosslinking of DNA strands, which results in cell death | doi:10.21037/jgo.2019.01.07  doi:10.21037/jgo.2019.01.01 |
| Vinorelbine | inhibition of microtubule dynamics leading to a mitotic arrest and cell death | doi:10.3390/cancers12040872  doi:10.1038/s12276-020-00524-4 |
| Nilotinib | tyrosine kinase inhibitors (TKIs) | doi:10.1074/jbc.M112.446385 |
| AZD8055 | MTOR inhibitor | doi:10.12659/MSM.907514 |
